# Myeloid‐Mas Signaling Modulates Pathogenic Crosstalk among MYC^+^CD63^+^ Endothelial Cells, MMP12^+^ Macrophages, and Monocytes in Acetaminophen‐Induced Liver Injury

**DOI:** 10.1002/advs.202306066

**Published:** 2024-02-13

**Authors:** Shuai Chen, Zhi Lu, Yudong Zhao, Lu Xia, Chun Liu, Siqing Zuo, Manchang Jin, Haoyu Jia, Shanshan Li, Shuo Zhang, Bo Yang, Zhijing Wang, Jing Li, Fei Wang, Changqing Yang

**Affiliations:** ^1^ Department of Gastroenterology and Hepatology Tongji Hospital, School of Medicine, Tongji University Shanghai 200092 China; ^2^ Department of Automation Tsinghua University Beijing 100084 China; ^3^ Institute for Brain and Cognitive Sciences Tsinghua University Beijing 100084 China; ^4^ Department of Liver Surgery, Renji Hospital, School of Medicine Shanghai Jiao Tong University Shanghai 200127 China; ^5^ School of Electrical and Information Engineering Tianjin University Tianjin 300072 China; ^6^ Division of Gastroenterology Seventh Affiliated Hospital of Sun Yat‐sen University Shenzhen 518107 China

**Keywords:** acetaminophen, drug‐induced liver injury, intravital imaging, Mas, microenvironment, sterile inflammation

## Abstract

Acetaminophen overdose is a leading cause of acute liver failure (ALF). Despite the pivotal role of the inflammatory microenvironment in the progression of advanced acetaminophen‐induced liver injury (AILI), a comprehensive understanding of the underlying cellular interactions and molecular mechanisms remains elusive. Mas is a G protein‐coupled receptor highly expressed by myeloid cells; however, its role in the AILI microenvironment remains to be elucidated. A multidimensional approach, including single‐cell RNA sequencing, spatial transcriptomics, and hour‐long intravital imaging, is employed to characterize the microenvironment in *Mas1* deficient mice at the systemic and cell‐specific levels. The characteristic landscape of mouse AILI models involves reciprocal cellular communication among MYC^+^CD63^+^ endothelial cells, MMP12^+^ macrophages, and monocytes, which is maintained by enhanced glycolysis and the NF‐κB/TNF‐α signaling pathway due to myeloid‐Mas deficiency. Importantly, the pathogenic microenvironment is delineated in samples obtained from patients with ALF, demonstrating its clinical relevance. In summary, these findings greatly enhance the understanding of the microenvironment in advanced AILI and offer potential avenues for patient stratification and identification of novel therapeutic targets.

## Graphical abstract

A multidimensional approach is applied to depict the microenvironment of mouse acetaminophen‐induced liver injury. Myeloid‐Mas deficiency drives the pathogenic, reciprocal cellular communication among MYC^+^CD63^+^ endothelial cells, MMP12^+^ macrophages, and monocytes. The landscape is maintained by enhanced glycolysis and NF‐κB/TNF‐α signaling. These findings show a significant clinical correlation with liver failure patients.

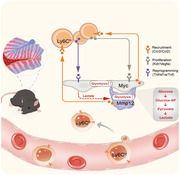

## 1 Introduction

Acetaminophen (APAP), a common antipyretic and analgesic, is the leading cause of drug‐induced liver injury (DILI) resulting from abuse or accidental overdose, necessitating heightened vigilance during the coronavirus disease 2019 pandemic.^[^ ^1^ ^]^ APAP‐induced liver injury (AILI) is also the primary cause of acute liver failure (ALF).^[^ ^2^ ^]^ Given its limited therapeutic alternatives, it is imperative to delve into the fundamental mechanisms underlying the progression of advanced AILI.^[^ ^3^ ^]^

AILI is an intrinsic form of DILI that exhibits predictability, reproducibility, and dose‐dependency.^[^ ^4^ ^]^ AILI mouse models are highly representative tools that have been extensively studied to understand the pathophysiology of human diseases. The toxicity of APAP is primarily due to its reactive metabolite N‐acetyl‐p‐benzoquinone imine, which rapidly depletes glutathione (GSH), disrupts mitochondrial function, causes nuclear DNA damage, and leads to extensive necrosis. N‐acetylcysteine (NAC), which replenishes GSH stores,^[^ ^5^ ^]^ is currently the only approved medication for APAP overdose. However, its efficacy diminishes if the time interval between overdose and treatment is prolonged, highlighting an unmet medical need to develop alternative or more efficacious therapeutic options in clinical practice. While previous research has predominantly focused on the intracellular mechanisms of hepatocyte death, it has been recognized over the past 1–2 decades that APAP overdose can induce a sterile inflammatory response characterized by the activation of Kupffer cells and the recruitment of peripheral immune cells. This cascade may involve the orchestrated removal of necrotic cells and facilitation of regeneration, or it may exacerbate the initial hepatocyte injury. There is a substantial knowledge gap regarding cellular crosstalk within the microenvironment in advanced AILI, which may constitute one of the fundamental mechanisms driving the progression from AILI to liver failure.^[^ ^2^ ^]^ Therefore, a comprehensive and high‐resolution cellular characterization of the microenvironment could contribute substantially to identifying novel therapeutic targets.

Mas, encoded by the oncogene *Mas1*, is a G protein‐coupled receptor widely expressed in human and murine tissues, with particularly elevated levels observed on myeloid cells.^[^ ^6^ ^]^ The beneficial effects of Mas activation have been documented in various diseases.^[^ ^7^, ^8^, ^9^, ^10^ ^]^ Our previous research revealed that the activation of Mas effectively attenuates hepatocyte death by enhancing fatty acid oxidation during the early stages of mouse AILI.^[^ ^11^ ^]^ However, the role of Mas in the AILI microenvironment remains to be elucidated. In this study, we employed a multidimensional approach, including single‐cell RNA sequencing (scRNA‐seq), spatial transcriptomics (ST), multiplex immunohistochemistry (mIHC), and intravital imaging to characterize the immunometabolic landscape of advanced AILI in *Mas1* deficient mice at the systemic and cell‐specific levels. The clinical significance of this characteristic microenvironment has been demonstrated in human liver samples. These findings could greatly enhance our understanding of the microenvironment in advanced AILI and offer potential avenues for patient stratification and identification of novel therapeutic targets.

## 2 Results

### 2.1 Single‐Cell Annotation of MYC^+^CD63^+^ Endothelial Cells (ECs) in the Mas‐Imprinted Mouse AILI Microenvironment

**Figure 1:**
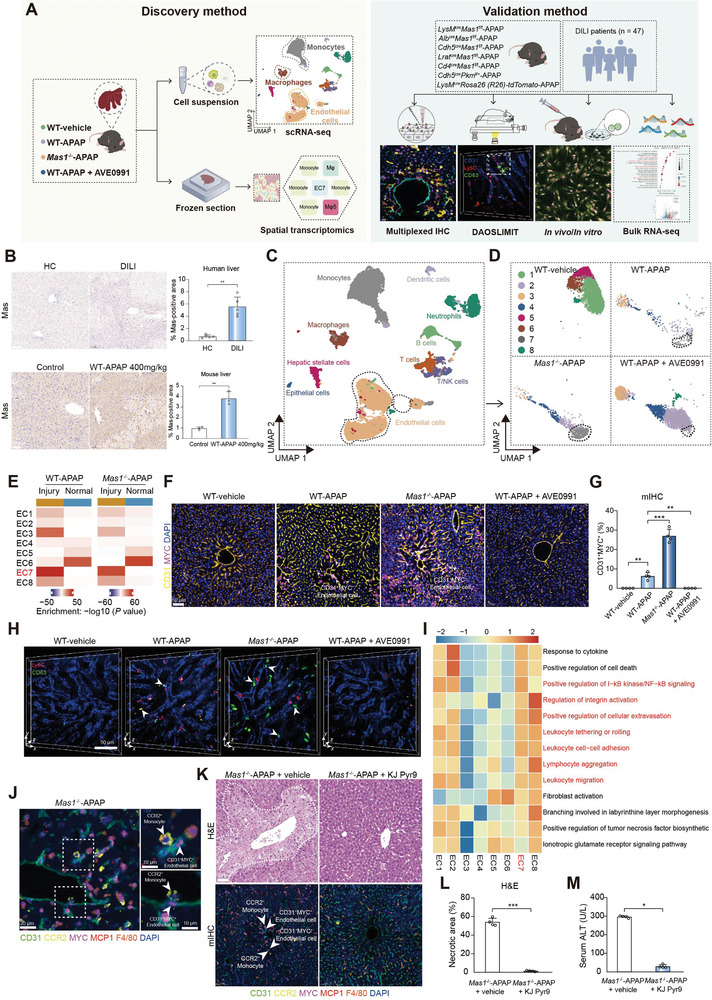
Single‐cell annotation of MYC^+^CD63^+^ ECs in Mas‐imprinted mouse AILI. A) Schematic of the experimental approach for discovering and validating functional immune cell subpopulations in AILI. B) Representative immunohistochemical staining and quantification of Mas in liver sections obtained from HC and DILI groups (upper panel; *n* = 5 samples per group; two‐sided Student's t‐test; *p* = 2.14 × 10^−3^). Scale bar: 100 µm. Representative histological staining and quantification of Mas in liver sections from WT mice treated with vehicle or APAP (lower panel; *n* = 3 samples per group; two‐sided Student's t‐test; *p* = 1.83 × 10^−3^). Scale bar: 50 µm. C) The uniform manifold approximation and projection (UMAP) plot of intrahepatic main cell types. D) The UMAP plot shows the subpopulations of ECs between different groups. E) The MIA map of EC clusters and ST‐defined regions. F) mIHC of CD31^+^MYC^+^ ECs. The white arrows represent the CD31^+^MYC^+^ ECs. Scale bar: 50 µm. G) Quantification of CD31^+^MYC^+^ ECs for mIHC as shown in F (*n* = 4 mice per group; One‐way ANOVA with Tukey's test, *p* = 3.91 × 10^−3^, 2.71 × 10^−8^ and 3.91 × 10^−3^ from left to right). H) Representative intravital images of CD63^+^ ECs by DAOSLIMIT. The white arrows represent the CD63^+^ ECs. Scale bar: 50 µm. I) Pathway enrichment analysis of EC clusters. GSVA was used to perform the pathway enrichment analysis (Methods). Only selected pathways were shown. J) mIHC of CD31^+^MYC^+^ ECs and CCR2^+^ monocytes. Scale bar: 20 µm (Left) and 10 µm (Right). In K–M, mice were administrated with or without KJ pyr9 2 h after APAP challenge (*n* = 4 mice per group). K) Representative hematoxylin‐eosin (H&E) staining and mIHC of CD31^+^MYC^+^ ECs and CCR2^+^ monocytes. Scale bar: 50 µm. L) Quantification of necrotic area for H&E as shown in K (*n* = 4 mice per group; two‐sided Student's t‐test, *p* = 2.62 × 10^−7^). M) Serum alaninetransaminase (ALT), a measure of hepatic injury (*n* = 4 mice per group; two‐sided Mann‐Whitney U test, *p* = 2.86 × 10^−2^). In all graphs data are presented as mean ± SD, *p < 0.05; **p < 0.01; ***p < 0.001.

The role of Mas signaling activation in hepatocytes has been elucidated using mouse AILI models in our previous study.^[^ ^11^ ^]^ Here, the multi‐omic analysis and intravital imaging approaches were applied to investigate the role of Mas in the microenvironment of mouse AILI (**Figure** **1**A). By evaluating the protein levels of cytochrome P450 isozyme (CYP2E1) and hepatic GSH, we initially determined that neither systemic Mas deficiency (*Mas1* ^−/‐^ mice) nor activation by AVE0991 (a specific Mas agonist) had a discernible impact on APAP metabolism (Figure S1A–D, Supporting Information).^[^ ^12^ ^]^ Intrahepatic Mas expression was significantly upregulated in human and mouse DILI (Figure 1B). The systemic deficiency of Mas was greatly exacerbated, while the activation of Mas substantially ameliorated the APAP‐induced infiltration of inflammatory cells in the liver (Figure S1E–H, Supporting Information). Hepatic neutrophils and monocytes in live mice were effectively monitored using digital adaptive optics scanning light‐field mutual iterative tomography (DAOSLIMIT) (Video S1, Supporting Information). Subsequently, scRNA‐seq enabled the identification of 26 distinct cell populations spanning ten major types, culminating in a comprehensive high‐resolution liver cell atlas (Figure 1C and Figure S1I–K, Supporting Information). Concurrently, the liver sections were partitioned into normal and injured regions for subsequent ST analysis (Figure S2A, Supporting Information). To integrate the scRNA‐seq and ST data, we used robust cell type decomposition (RCTD) to resolve the cellular composition of a single spot encompassing a heterogeneous mixture of distinct cell types.^[^ ^13^ ^]^ The *Mas1* ^−/−^‐APAP group exhibited significantly larger areas of injury with more pronounced inflammatory infiltration than the wild type‐APAP (WT‐APAP) group (Figure S2B, Supporting Information).

ECs, uniquely positioned within the vascular lumen, are one of the earliest cell types to perceive danger and play a pivotal role in initiating and advancing injury progression.^[^ ^14^ ^]^ Hence, we initially analyzed clusters of CD31^+^ ECs (Figure S2C, Supporting Information). Notably, cluster 7 of ECs (EC7) exhibited specific markers, including *Myc*, *Cd63*, and *Il17ra* (Figure S2D, Supporting Information), which were significantly enriched in the liver of *Mas1* ^−/−^‐APAP mice while almost completely diminished after systemic Mas activation (Figure 1D and Figure S2E, Supporting Information). Multimodal intersection analysis (MIA) was employed to integrate the scRNA‐seq and ST datasets.^[^ ^15^ ^]^ Notably, EC7 was exclusively observed in injured regions (Figure 1E). Besides, EC7 exhibited a prominent “pro‐inflammatory” profile, as evidenced by their highest scores for apoptosis, necroptosis, NF‐κB, and TNF signaling pathways among all clusters (Figure S2F, Supporting Information). For protein‐level validation, mIHC was conducted to identify MYC^+^ ECs that exhibited significant enrichment in the *Mas1* ^−/−^‐APAP group and were scarcely observed after systemic Mas activation (Figure 1F,G and Figure S3A,B, Supporting Information). The enrichment of MYC^+^ ECs was significantly associated with both the dosage of APAP administered and the extent of liver injury (Figure S3C, Supporting Information). Moreover, the localization pattern of CD63^+^ ECs was consistent with that of MYC^+^ ECs (Figure S3D, Supporting Information). Additionally, flow cytometry revealed the CD63^+^ ECs enrichment in the *Mas1* ^−/−^‐APAP group (Figure S3E, Supporting Information). Furthermore, DAOSLIMIT provides a visual representation of the hepatic distribution of CD63^+^ ECs in live mice (Figure 1H). The MYC^+^ ECs were further characterized as liver sinusoidal endothelial cells (LSECs) based on their co‐expression of CD105, CD146, and LYVE1, which are well‐established markers for LSECs (data not shown).^[^ ^16^, ^17^, ^18^ ^]^ The functional characteristics of ECs were delineated by employing gene set variation analysis (GSVA) in clusters. EC7 exhibited a prominent functional profile encompassing leukocyte migration, adhesion, and rolling (Figure 1I). During ALF, a common MYC‐dependent transcriptional program has been reported to activate ECs and recruit Ly6C^+^ monocytes into the liver.^[^ ^19^ ^]^ The study found a consistent association between CCR2^+^ monocytes and MYC^+^ ECs in close proximity (Figure 1J). Pharmacological inhibition of MYC using KJ‐Pyr‐9 significantly improved the disease phenotype of *Mas1* ^−/−^‐APAP mice by suppressing the intrahepatic infiltration of CCR2^+^ monocytes without affecting APAP metabolism (Figure 1K–M and Figure S3F,G, Supporting Information).^[^ ^19^, ^20^ ^]^ This suggests that MYC‐dependent monocyte recruitment is crucial for disease pathogenesis. Overall, MYC^+^CD63^+^ ECs with a pro‐inflammatory phenotype were annotated in the Mas‐imprinted mouse AILI microenvironment.

### 2.2 Lactate Derived from ECs Induces Glycolysis and Polarizes Macrophages (Mψ) toward a Mixed M1/M2‐like Phenotype

**Figure 2:**
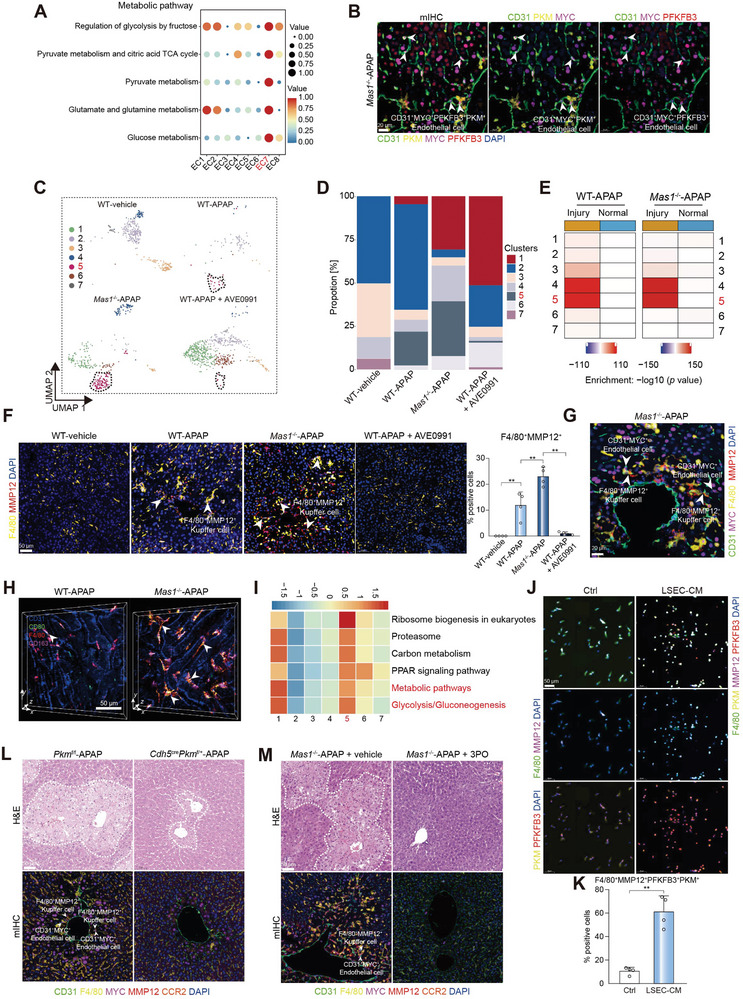
EC‐sourced lactate polarizes Mψ to the mixed M1/M2‐like phenotype by inducing glycolysis. A) The metabolic pathway activity analysis of EC clusters. The circle size and color darkness both represent the scaled metabolic score. B) mIHC of CD31^+^MYC^+^ ECs and key glycolytic enzymes (PKM and PFKFB3). Scale bar: 20 µm. C) The UMAP plot shows the subpopulation of Mψ between groups. D) Histogram showing the proportion of each cluster between groups. E) The MIA map of Mψ clusters and ST‐defined regions. F) mIHC of F4/80^+^MMP12^+^ Mψ. Scale bar: 50 µm. The white arrows represent the F4/80^+^MMP12^+^ Mψ, and the quantification of F4/80^+^MMP12^+^ Mψ is shown (*n* = 4 mice per group; One‐way ANOVA with Tukey's test, *p* = 8.41 × 10^−4^, 1.59 × 10^−3^ and 1.72 × 10^−3^ from left to right). G) mIHC of CD31^+^MYC^+^ ECs and F4/80^+^MMP12^+^ Mψ. Scale bar: 20 µm. H) Representative intravital images of Mψ polarization by DAOSLIMIT. The white arrows represent the CD80^+^CD163^+^ Mψ. Scale bar: 50 µm. I) Pathway enrichment analysis of Mψ clusters. GSVA was used to perform the pathway enrichment analysis (Methods). Only selected pathways were shown. J) Multiplex fluorescence of F4/80^+^MMP12^+^ kupffer cells and key glycolytic enzymes (PKM and PFKFB3). Scale bar: 50 µm. The mouse primary kupffer cells were treated with or without LSEC‐CM for 24 h. K) Quantification of F4/80^+^MMP12^+^PKM^+^PFKFB3^+^ kupffer cells for multiplex fluorescence as shown in J (*n* = 4 samples per group; two‐sided Student's t‐test, *p* = 3.63 × 10^−3^). L) Representative H&E staining and mIHC of CD31^+^MYC^+^ ECs and F4/80^+^MMP12^+^ Mψ. Scale bar: 50 µm. *Pkm* ^f/f^ and *Cdh5* ^cre^ *Pkm* ^f/+^ mice were administrated with APAP for 24 h (*n* = 4 mice per group). M) Representative H&E staining and mIHC of CD31^+^MYC^+^ ECs, F4/80^+^MMP12^+^ Mψ, and CCR2^+^ monocytes. Scale bar: 50 µm. *Mas1* ^−/−^ mice were administrated with or without 3PO under APAP challenge (*n* = 4 mice per group). In all graphs, data are presented as mean ± SD, **p < 0.01.

The overdose of APAP is linked to lactic acidosis, oxidative stress, and mitochondrial dysfunction.^[^ ^12^, ^21^ ^]^ Considering the documented influence of Mas on hepatocyte metabolism in mouse AILI and the significant contribution of metabolic status to cellular function, we analyzed the metabolic characteristics of ECs, which predominantly rely on glycolysis.^[^ ^22^ ^]^ The glycolytic activity of EC7 was the highest among all clusters (**Figure** **2**A), which exhibited a significant upregulation in the EC transition trajectory (Figure S4, Supporting Information). Subsequently, mIHC was utilized to evaluate the protein expression of PKM and PFKFB3, two pivotal rate‐limiting enzymes in glycolysis,^[^ ^22^, ^23^ ^]^ in MYC^+^ ECs in the *Mas1* ^−/−^‐APAP group (Figure 2B). Considering the EC's capacity to generate ample glycolytic metabolites, particularly lactate, which can induce glycolysis in local Mψ relying predominantly on glycolysis as their primary metabolic pathway,^[^ ^24^ ^]^ we propose that EC7 may play a crucial role in modulating the functional phenotype of Mψ. Hence, we initially used mIHC to ascertain if MYC^+^ ECs are spatially closer to Mψ than neutrophils or T cells (Figure S5A, Supporting Information). Subsequently, we identified seven distinct clusters of Mψ based on their unique marker‐gene expression profiles. Notably, the presence of Mψ cluster 5 (Mψ5), characterized by expressing *Mmp12* and *Mmp13* (Figure S5B, Supporting Information), was significantly enriched in the *Mas1* ^−/−^‐APAP group, but almost completely diminished following AVE0991 administration (Figure 2C,D). The presence of Mψ5 was exclusively observed in the injured regions in ST (Figure 2E), as confirmed by mIHC analysis (Figure 2F). The findings of Mψ5 are highly consistent with those of EC7. Mψ5 were further characterized as Kupffer cells (data not shown) based on the conventional Kupffer cell markers including *Adgre1*, *Clec4f*, *Timd4*, and *Mafb*.^[^ ^25^ ^]^ Additionally, Mψ5 exhibited a pro‐inflammatory phenotype (Figure S5C, Supporting Information). Mψ exhibits high plasticity and can adopt classically activated (M1, pro‐inflammatory) or alternatively activated (M2, anti‐inflammatory) phenotypes in response to diverse environmental stimuli.^[^ ^26^ ^]^ In the study, Mψ5 was identified as exhibiting a mixed M1/M2‐like phenotype, characterized by high expression levels of both M1 and M2 markers (Figure S6A,B, Supporting Information). Additionally, flow cytometry analysis revealed a significant increase in the population of Mψ (CD45^+^CD11b^+^F4/80^hi^Ly6C^lo^) that co‐expressed CD86 (M1 marker) and CD206 (M2 marker) in the *Mas1* ^−/−^‐APAP group (Figure S6C, Supporting Information). Moreover, Mψ5 expressed both pro‐inflammatory and pro‐angiogenic factors (Figure S6D, Supporting Information). Of note, DAOSLIMIT revealed the hepatic enrichment of CD86^+^CD206^+^ Mψ in the *Mas1* ^−/−^‐APAP group (Figure 2H and Video S2, Supporting Information). It has been previously reported that ECs can produce lactate, which triggers glycolysis in adjacent Mψ and polarizes them towards a mixed M1/M2‐like phenotype.^[^ ^22^ ^]^ The mIHC technique was initially utilized to demonstrate the high adjacency between MMP12^+^ Mψ and MYC^+^ ECs in necrotic regions (Figure 2G and Figure S7A, Supporting Information). Additionally, the scRNA‐seq metabolic analysis revealed that glycolysis‐related pathways were most significantly enriched in Mψ 5 among all clusters (Figure 2I). This was further confirmed through the mIHC evaluation of PFKFB3 protein expression levels in MMP12 + Mψ cells (Figure S7B, Supporting Information), and it was found to be significantly upregulated along Mψ transition trajectory (Figure S8A–E, Supporting Information). Furthermore, pharmacological inhibition of MYC exhibited a significant decrease in hepatic MMP12^+^ Mψ, underscoring the pivotal role of MYC signaling in maintaining this specific population and indicating potential intercellular communication between EC7 and Mψ5 (Figure S8F, Supporting Information). To investigate the impact of ECs on Mψ phenotype, primary Kupffer cells were exposed to a conditioned medium (CM) derived from activated LSECs. The CM induced a significant, pronounced upregulation of key enzymes for glycolysis and the emergence of CD86^+^CD206^+^ Mψ, indicating a correlation between glycolysis and the mixed M1/M2‐like phenotype (Figure 2J,K and Figure S9A–C, Supporting Information). Additionally, lactate elicited alterations in Kupffer cell function comparable to the CM (Figure S9D–H, Supporting Information). Moreover, α‐cyano‐4‐hydroxycinnamic acid, an inhibitor of monocarboxylate transporters effectively reversed the glycolysis induced by the CM in Kupffer cells (Figure S9I,M, Supporting Information), highlighting the crucial role of lactate cellular uptake in the CM. To elucidate the involvement of glycolysis in ECs in mouse AILI, we generated *Cdh5* ^cre^ *Pkm* ^f/+^ mice (Figure S10A, Supporting Information). Remarkably, these mice exhibited significantly ameliorated liver injury compared to *Pkm* ^f/f^‐APAP mice, without any discernible impact on APAP metabolism (Figure 2L and Figure S10B–F, Supporting Information). The glycolysis inhibitor 3PO effectively ameliorated the disease phenotype of *Mas1* ^−/−^‐APAP mice by antagonizing PFKFB3,^[^ ^28^ ^]^ resulting in a reduction in hepatic MMP12^+^ Mψ and MYC^+^ ECs (Figure 2M). This reveals the potential contribution of glycolysis‐dependent cellular crosstalk to AILI pathogenesis. However, it had a noticeable impact on APAP metabolism (Figure S10G–O, Supporting Information).

### 2.3 Monocyte‐derived TNF‐α Modulates MYC^+^CD63^+^ ECs Through TNFR1

**Figure 3:**
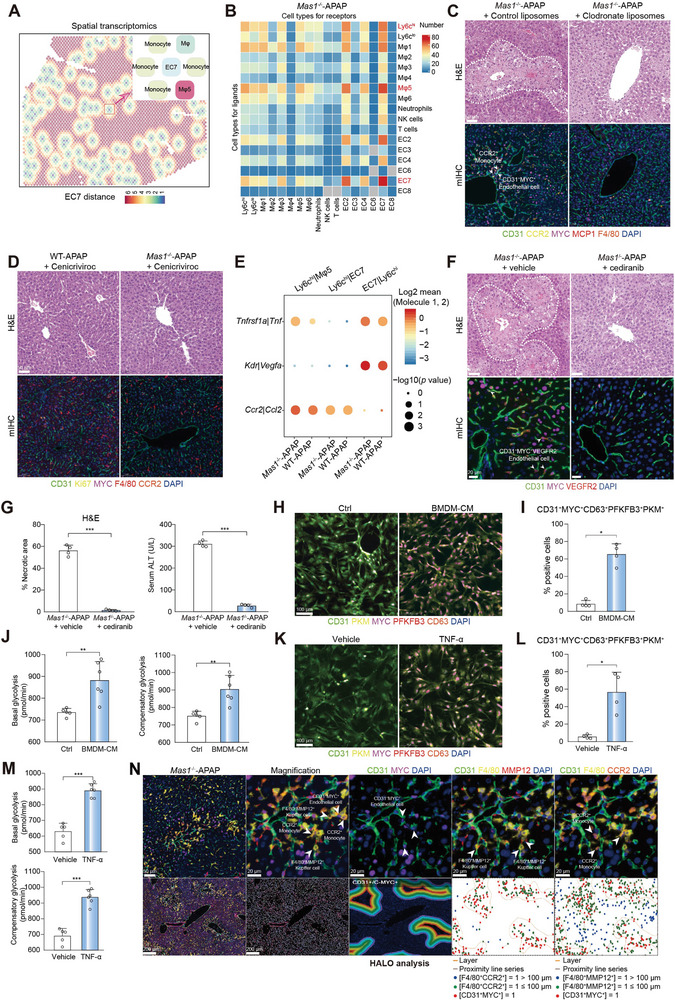
Monocyte‐derived TNF‐α acts on MYC^+^CD63^+^ EC through TNFR1. A) Spatial feature plots showing the cell types in EC7 expansion units for ST. B) CellPhoneDB analysis showing the cell–cell interactions for scRNA‐seq. C) *Mas1* ^−/−^ mice were pre‐administrated with clodronate liposomes or control liposomes for 24 h before APAP challenge (*n* = 4 per group). Representative stainings of H&E and mIHC are shown. Scale bar: 50 µm. D) WT and *Mas1* ^−/−^ mice were pre‐administrated with cenicriviroc for 2 h before APAP challenge (*n* = 4 per group). Representative stainings of H&E and mIHC are shown. Scale bar: 50 µm. E) CellPhoneDB analysis showing the major interaction pairs between Ly6c^hi^ monocyte‐Mψ5 or Ly6c^hi^ monocyte‐EC7 for scRNA‐seq. In F‐G, *Mas1* ^−/−^ mice were pre‐administrated with cediranib or control for 2 h before APAP challenge (*n* = 4 per group). F) Representative staining of H&E and mIHC are shown. Scale bar: 50 µm. G) Quantification of necrotic area for H&E as shown in F (*n* = 4 mice per group; two‐sided Student's t‐test; *p* = 1.55 × 10^−4^) and serum ALT (*n* = 4 mice per group; two‐sided Student's t‐test; *p* = 2.64 × 10^−8^). H) Multiplex fluorescence of CD31^+^MYC^+^CD63^+^ LSECs and key glycolytic enzymes (PKM and PFKFB3). Scale bar: 100 µm. The mouse primary LSECs were treated with or without BMDM‐CM for 24 h. I) Quantification of CD31^+^MYC^+^CD63^+^PFKFB3^+^PKM^+^ LSECs for multiplex fluorescence as shown in H (*n* = 4 samples per group; two‐sided Mann‐Whitney U test, *p* = 2.86 × 10^−2^). J) Glycolytic rate assay showing glycolytic function of LSECs treated with or without BMDM‐CM for 24 h. (*n* = 5 or 6 samples per group; two‐sided Student's t‐test, *p* = 7.50 × 10^−3^ and 3.77 × 10^−3^ from left to right). K) Multiplex fluorescence of CD31^+^MYC^+^CD63^+^ LSECs and key glycolytic enzymes (PKM and PFKFB3). Scale bar: 100 µm. The mouse primary LSECs were treated with or without TNF‐α (20 ng ml^−1^) for 24 h. L) Quantification of CD31^+^MYC^+^CD63^+^PFKFB3^+^PKM^+^ LSECs for multiplex fluorescence as shown in K (*n* = 4 samples per group; two‐sided Student's t‐test, *p* = 2.00 × 10^−2^). M) Glycolytic rate assay showing glycolytic function of LSECs treated with or without TNF‐α (20 ng ml^−1^) for 24 h. (*n* = 5 or 6 samples per group; two‐sided Student's t‐test, *p* = 8.00 × 10^−6^ and 1.30 × 10^−5^ from top to bottom). N) mIHC of CD31^+^MYC^+^ ECs, F4/80^+^MMP12^+^ Mψ and CCR2^+^ monocytes are shown. Scale bar: 200 µm, 50 µm and 20 µm. Spatial localizations of CD31^+^MYC^+^ ECs, CCR2^+^ monocytes, and F4/80^+^MMP12^+^ Mψ within injured region are shown. In all graphs data are presented as mean ± SD, *p < 0.05; **p < 0.01; ***p < 0.001.

The functional phenotype of ECs is not constant and can be influenced by microenvironment factors.^[^ ^14^ ^]^ Hence, we performed CellphoneDB analysis to investigate the interplay between MYC^+^ ECs and hepatic immune cells. Importantly, both scRNA‐seq and ST analysis of WT‐APAP mice revealed a significant interaction among EC7, Mψ5, and monocytes (Figure S11A–C, Supporting Information). To further elucidate the anatomical basis of the cellular interactions with EC7, we used ST data to classify EC7‐localized spots as intra‐spots (representing the closest interaction), inter‐spots (representing secondary proximity), or distant spots (indicating minimal interaction) (Figure S11D, Supporting Information). Considering the spatial proximity between cells with enriched interactive signals, we designated these regions (intra‐ and inter‐spots) as expansion units, with EC7 situated within the intra‐spots and engaged in interactions with the surrounding inter‐spots. In most of the expansion units, EC7 cells were found to be closely associated with Mψ5 and monocytes, indicating their intimate interactions (**Figure** **3**A). Furthermore, the presence of a three‐cell interaction was enriched in the *Mas1* ^−/−^‐APAP group and this observation was confirmed through mIHC (Figure 3B and Figure S11E,F, Supporting Information). These findings suggest the potential involvement of monocytes in the phenotypic transition of ECs in *Mas1* ^−/−^‐APAP mice. Therefore, clodronate liposomes were used to indiscriminately deplete Kupffer cells and monocytes in *Mas1* ^−/−^‐APAP and WT‐APAP mice,^[^ ^29^ ^]^ resulting in a significantly mitigated liver injury accompanied by a reduced number of hepatic MYC^+^ ECs (Figure 3C and Figure S12A–G, Supporting Information). We subsequently used cenicriviroc (a dual CCR2/CCR5 antagonist) to selectively suppress monocyte infiltration into the liver in the WT‐APAP and *Mas1* ^−/−^‐APAP groups.^[^ ^30^ ^]^ Cenicriviroc not only significantly ameliorated the AILI phenotype, but also reduced the phenotypic variations between the two groups (Figure 3D). These findings demonstrated the substantial contribution of monocytes to the disease phenotype in *Mas1* ^−/−^‐APAP mice.

We subsequently used paired ligand‐receptor (L‐R) analyses to investigate three‐cell interactions. The findings suggested that EC7 and Mψ5 mediated the recruitment of monocytes into the liver through the *Ccl2*‐*Ccr2* axis, while monocytes interacted with EC7 via *Tnf*‐*Tnfr1* and *Vegfa*‐*Kdr* axes. The above L‐R pairs were particularly enriched in the *Mas1* ^−/−^‐APAP group (Figure 3E). The expression of *Kdr* (VEGFR2) was significantly upregulated in EC7, and their proliferation rate was markedly increased, as demonstrated by Ki‐67 staining on mIHC (Figure S12H,I, Supporting Information). CCR2^+^ monocytes were also enriched in the necrotic areas (Figure S12J, Supporting Information), suggesting their association with the proliferation of VEGFR2^+^MYC^+^ ECs. Next, the administration of cediranib (a potent inhibitor of VEGFR) in *Mas1* ^−/−^‐APAP mice resulted in a significant amelioration of liver injury and a reduction in the hepatic MYC^+^ EC population while exhibiting no impact on APAP metabolism (Figure 3F,G and Figure S12K–M, Supporting Information). Additionally, the dynamic expression of TNF and NF‐κB signaling pathways in monocytes was upregulated along the pseudotime trajectory (Figure S13A–E, Supporting Information), indicating an enhanced interaction between monocytes and EC7 through the TNF‐TNFR1 axis. Furthermore, we investigated whether the emergence of EC7 was induced by TNF‐α derived from monocytes. To mimic an in vivo microenvironment, primary LSECs were incubated with bone marrow‐derived macrophage‐CM (BMDM‐CM). Notably, BMDM‐CM demonstrated a remarkable capacity to induce the emergence of MYC^+^CD63^+^ LSECs, which exhibited high glycolytic activity through the expression of both PFKFB3 and PKM (Figure 3H–J). Compared to BMDM‐CM, the administration of recombinant TNF‐α resulted in a comparable upregulation of PFKFB3^+^PKM^+^MYC^+^CD63^+^ LSECs (Figure 3K–M and Figure S13F, Supporting Information). Importantly, mIHC analysis revealed a high degree of spatial proximity among MMP12^+^ Mψ, CCR2^+^ monocytes, and MYC^+^ ECs in necrotic areas, particularly in the *Mas1* ^−/−^‐APAP group (Figure 3N).

Taken together, our findings suggest that TNF‐α derived from monocytes induces the emergence of MYC^+^CD63^+^ ECs, which subsequently sustain a pro‐inflammatory microenvironment characterized by hyperactivated glycolysis in AILI.

### 2.4 Myeloid‐Mas Deficiency Maintains a Highly Pro‐Inflammatory and Glycolytic Microenvironment in Mouse AILI

**Figure 4:**
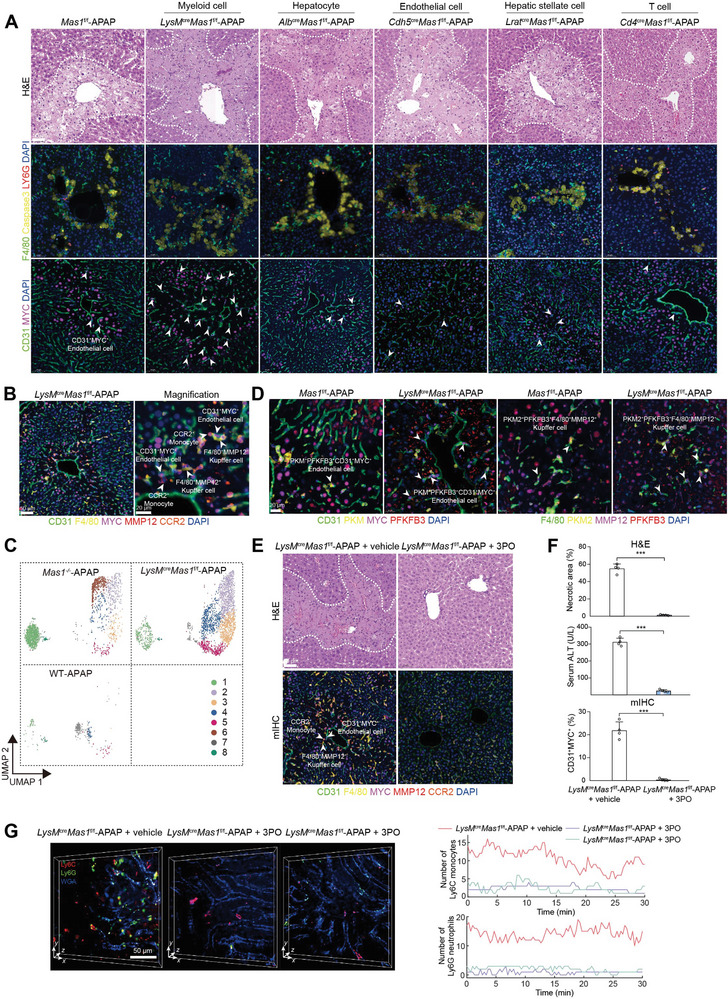
Myeloid Mas modulates AILI through the highly pro‐inflammatory and glycolytic microenvironment. A) *Mas1* ^f/f^, *LysM* ^cre^ *Mas1* ^f/f^, *Alb* ^cre^ *Mas1* ^f/f^, *Cdh5* ^cre^ *Mas1* ^f/f^, *Lrat* ^cre^ *Mas1* ^f/f^ and *Cd4* ^cre^ *Mas1* ^f/f^ mice were administrated with APAP for 24 h (*n* = 6 mice per group). Representative stainings of H&E and mIHC are shown. Scale bar: 50 µm. B) mIHC of CD31^+^MYC^+^ ECs, F4/80^+^MMP12^+^ Mψ, and CCR2^+^ monocytes are shown. Scale bar: 50 µm and 20 µm. C) The UMAP plot showing the subpopulation of Mψ. D) mIHC of CD31^+^MYC^+^ ECs, F4/80^+^MMP12^+^ Mψ, and key glycolytic enzymes (PKM and PFKFB3). Scale bar: 20 µm. E) *LysM* ^cre^ *Mas1* ^f/f^ mice were pre‐administrated with or without 3PO before APAP challenge (*n* = 4 per group). Representative stainings of H&E and mIHC are shown. Scale bar: 50 µm. F) Quantification of necrotic area for H&E as shown in E (*n* = 4 mice per group; two‐sided Student's t‐test, *p* = 9.53 × 10^−7^), serum ALT (*n* = 4 mice per group; two‐sided Student's t‐test, *p* = 3.77 × 10^−7^) and quantification of CD31^+^MYC^+^ ECs for mIHC as shown in E (*n* = 4 mice per group; two‐sided Student's t‐test, *p* = 2.70 × 10^−5^). G) Timelapse data of neutrophils (Ly6G) and monocytes (Ly6C) in the vessels (WGA) of living mouse livers were captured by DAOSLIMIT. Representative intravital images and the temporal traces of their number are shown. *LysM* ^cre^ *Mas1* ^f/f^ mice were pre‐administrated with or without 3PO before APAP challenge. Scale bar: 50 µm. In all graphs, data are presented as mean ± SD, ***p < 0.001.

To further elucidate how Mas modulates the AILI microenvironment, we generated multiple cell‐specific *Mas1* knockout mice including *LysM* ^cre^ *Mas1* ^f/f^, *Alb* ^cre^ *Mas1* ^f/f^, *Cdh5* ^cre^ *Mas1* ^f/f^, *Lrat* ^cre^ *Mas1* ^f/f^, and *Cd4* ^cre^ *Mas1* ^f/f^ mice, that were then challenged with APAP. Notably, the AILI phenotype in *LysM* ^cre^ *Mas1* ^f/f^‐APAP mice was significantly aggravated (**Figure** **4**A, Figure S14A–G, and Video S3, Supporting Information). Moreover, in accordance with *Mas1^−^* ^/−^‐APAP mice, MYC^+^ ECs and MMP12^+^ Mψ exhibited significant enrichment in *LysM* ^cre^ *Mas1* ^f/f^‐APAP mice and were found to be closely associated with CCR2^+^ monocytes in necrotic regions, thereby establishing the microenvironment as previously described (Figure 4B and Figure S15, Supporting Information). These findings demonstrated the significance of myeloid Mas in modulating the AILI microenvironment.

To enhance the comprehensiveness of this study, we administered a higher dose of APAP (600 mg kg^−1^) during the early (3 and 6 h) and late stages (24 h) of AILI. The phenotypic differences between the two groups were statistically insignificant during the early stages (3 and 6 h) of AILI (Figure S14H–J, Supporting Information). The absence of a distinct microenvironment was also confirmed by mIHC staining during the early stages (3 and 6 h) of AILI (Figure S14K, Supporting Information). Notably, the exacerbated disease phenotype and a characteristic microenvironment with enriched MYC^+^ ECs and MMP12^+^ Mψ in *LysM* ^cre^ *Mas1* ^f/f^‐APAP mice were observed in the late stage (24 h) of AILI (Figure S14L‐O, Supporting Information), suggesting that the myeloid‐Mas signaling deficiency exacerbates liver injury in advanced AILI by modulating the inflammatory microenvironment, instead of influencing cell death during the early stages. Considering the critical role of MYC^+^ ECs in the AILI microenvironment, we also used scRNA‐seq to determine whether endothelial Mas deficiency directly induces the emergence of MYC^+^ EC but yielded insignificant findings (data not shown).

Of note, scRNA‐seq analysis of the *LysM* ^cre^ *Mas1* ^f/f^‐APAP group revealed a striking similarity to the *Mas1^−^* ^/−^‐APAP group, in terms of EC, Mψ, and monocytes (Figure S16A,B, Supporting Information). The EC cluster 2/4/6 observed in *LysM* ^cre^ *Mas1* ^f/f^‐APAP mice exhibited a strong positive correlation with EC7 in *Mas1^−^* ^/−^‐APAP mice (Figure 4C and Figure S16C–E, Supporting Information). Consistently, mIHC analysis revealed a significant enrichment of MYC^+^CD63^+^ ECs in the necrotic regions of *LysM* ^cre^ *Mas1* ^f/f^‐APAP mice (Figure S16F,G, Supporting Information). Moreover, flow cytometry revealed a significant increase in the number of CD63^+^ ECs in the *LysM* ^cre^ *Mas1* ^f/f^‐APAP group (Figure S16H, Supporting Information). Moreover, the MYC inhibitor significantly ameliorated the AILI phenotype in *LysM* ^cre^ *Mas1* ^f/f^‐APAP mice with a substantial reduction in hepatic monocytes (Figure S16I, Supporting Information).

Consistent with our previous findings, these MYC^+^ ECs exhibited significantly elevated expression of VEGFR2 and were found near monocytes (Figure S17A,B, Supporting Information). The inhibition of VEGFR2 significantly ameliorated liver injury, accompanied by a marked reduction in MYC^+^ EC and MMP12^+^ Mψ (Figure S17C–E, Supporting Information). Subsequently, we hypothesized that these MYC^+^ ECs might also elicit glycolysis in neighboring Mψ. For this aim, we performed scRNA‐seq analysis of Mψ clusters. As expected, the Mψ clusters observed in *LysM* ^cre^ *Mas1* ^f/f^‐APAP mice exhibited remarkable similarity to those found in *Mas1^−^* ^/−^‐APAP mice and clusters 1/2 in *LysM* ^cre^ *Mas1* ^f/f^‐APAP mice demonstrated a strong correlation with Mψ5 (MMP12^+^) in *Mas1^−^* ^/−^‐APAP mice (Figure S18A–C, Supporting Information). MMP12^+^ Mψ were also observed in necrotic areas, adjacent to MYC^+^ ECs in *LysM* ^cre^ *Mas1* ^f/f^‐APAP mice (Figure S18D,E, Supporting Information). Additionally, mIHC, flow cytometry, and DAOSLIMIT analyses revealed a significant augmentation of Mψ exhibiting a mixed M1/M2 phenotype in *LysM* ^cre^ *Mas1* ^f/f^‐APAP mice (Figure S18F–H, and Video S2, Supporting Information). Importantly, scMetabolism analysis revealed significantly elevated glycolytic activity in EC (clusters 2/4/6) and Mψ (clusters 1/2), which was further confirmed by mIHC (Figure 4D and Figure S19, Supporting Information). Furthermore, the inhibition of glycolysis effectively ameliorated the AILI phenotype observed in *LysM* ^cre^ *Mas1* ^f/f^‐APAP mice, thereby highlighting the crucial role of glycolysis within the AILI microenvironment (Figure 4E–G and Video S4, Supporting Information).

Collectively, myeloid Mas modulates mouse AILI by influencing the highly pro‐inflammatory and glycolytic microenvironments.

### 2.5 Myeloid Mas Modulates the AILI Microenvironment Through the NF‐κB/TNF‐α Pathway

**Figure 5:**
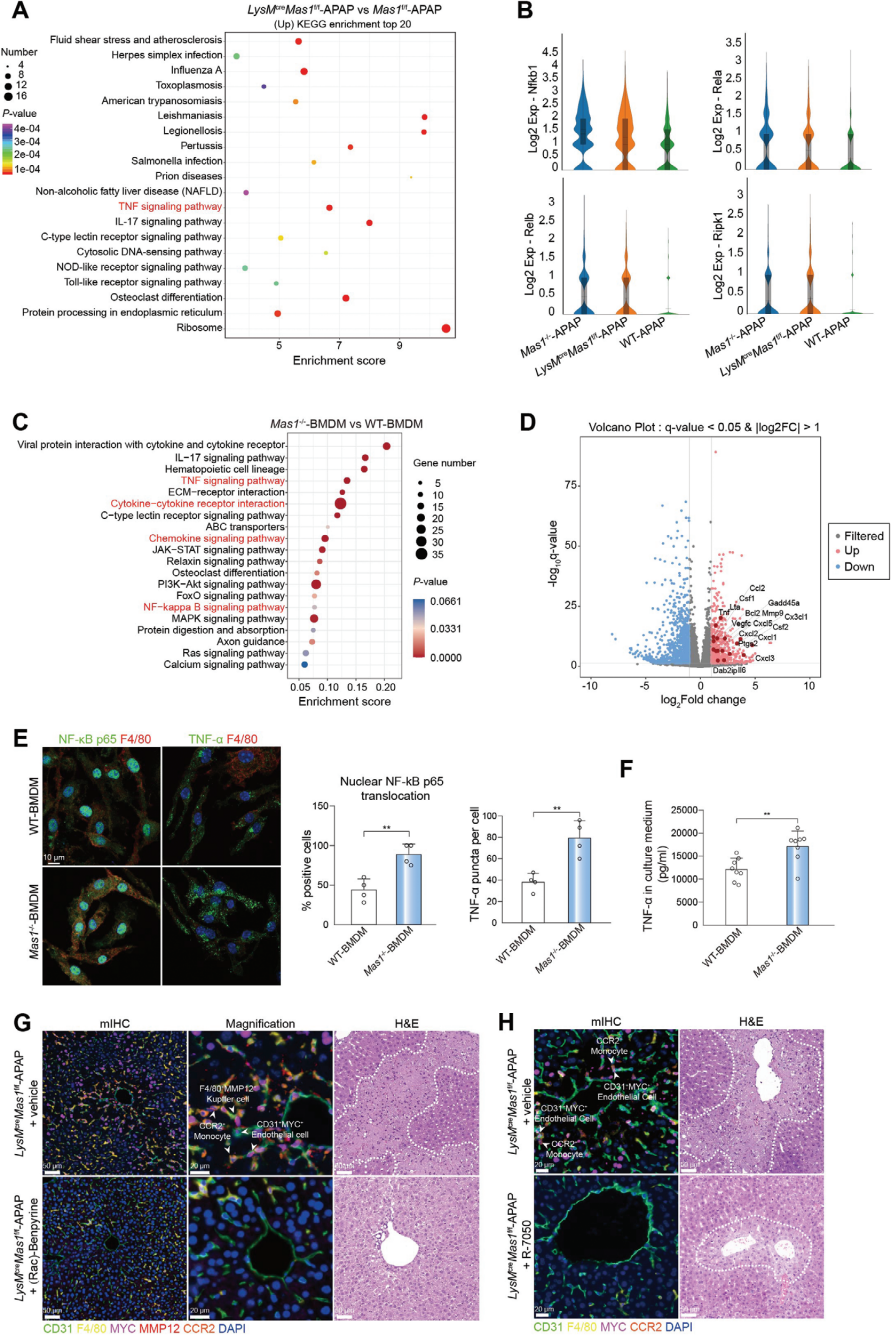
Myeloid Mas modulates AILI microenvironment via the NF‐κB/TNF‐α pathway. A) Kyoto Encyclopedia of Genes and Genomes (KEGG) pathway enrichment analysis of DEGs in *LysM* ^cre^ *Mas1* ^f/f^‐APAP versus *Mas1* ^f/f^‐APAP groups for monocytes. The top twenty pathways are presented. B) Violin plots showing the key genes of TNF signaling pathway and NF‐kappa B signaling pathway for monocytes between groups. In C‐D, BMDMs from WT and *Mas1* ^−/‐^ mice were stimulated with LPS (100 ng ml^−1^) for 12 h to drive pro‐inflammatory activation. Activated BMDMs were collected for bulk RNA‐seq (*n* = 5 or 6 samples per group). C) KEGG pathway enrichment analysis of DEGs in *Mas1* ^−/−^ versus WT BMDMs. The top twenty pathways are presented. D) Volcano plot showing the DEGs in *Mas1* ^−/−^ versus WT BMDMs for TNF signaling pathway and NF‐kappa B signaling pathway. E) BMDMs from WT and *Mas1* ^−/‐^ mice were stimulated with LPS (100 ng ml^−1^) for 12 h to drive pro‐inflammatory activation. Representative IF images and the statistical results of nuclear translocation of p65 (*n* = 4 samples per group; two‐sided Student's t‐test, *p* = 3.44 × 10^−3^) and TNF‐α (*n* = 4 samples per group; two‐sided Student's t‐test, *p* = 4.07 × 10^−3^) are shown. Scale bar: 10 µm. F) BMDMs from WT and *Mas1* ^−/−^ mice were stimulated with LPS (100 ng ml^−1^) for 12 h to drive proinflammatory activation. The TNF‐α of the CM were tested (*n* = 8 samples per group; two‐sided Student's t‐test, *p* = 4.14 × 10^−3^). G) *LysM* ^cre^ *Mas1* ^f/f^ mice were pre‐administrated with or without (Rac)‐Benpyrine (30 mg kg^−1^, ig) before APAP challenge (*n* = 4 mice per group). Representative stainings of H&E and mIHC are shown. Scale bar: 50 µm and 20 µm. H) *LysM* ^cre^ *Mas1* ^f/f^ mice were pre‐administrated with or without R‐7050 (10 mg kg^−1^, ip) for 2 h before APAP challenge (*n* = 4 mice per group). Representative stainings of H&E and mIHC are shown. Scale bar: 50 µm and 20 µm. In all graphs, data are presented as mean ± SD, **p < 0.01.

To uncover how myeloid Mas modulates the AILI microenvironment, we initially conducted scRNA‐seq analysis on monocytes, which revealed significant upregulation of the TNF pathway and its associated genes in the *LysM* ^cre^ *Mas1* ^f/f^‐APAP group (**Figure** **5**A,B). Besides, bulk RNA‐seq analysis of BMDM revealed that Mas deficiency could lead to a significant upregulation of NF‐κB and TNF signaling pathways, as evidenced by the differentially expressed genes (DEGs) depicted in the volcano plot (Figure 5C,D). Immunofluorescence (IF), enzyme‐linked immunosorbent assay (ELISA), and western blot (WB) were used to confirm the above findings (Figure 5E,F, and Figure S20A, Supporting Information). Consistently, mIHC revealed the close spatial relationship between TNF‐α^+^CCR2^+^ monocytes and TNFR1^+^MYC^+^ ECs in necrotic regions (Figure S20B,C, Supporting Information). Moreover, the administration of AVE0991 in vitro was found to significantly down‐regulate NF‐κB and TNF signaling pathways, as well as suppress TNF‐α production in BMDM, as evidenced by bulk RNA‐seq analysis (Figure S21A–D, Supporting Information). To determine clinical relevance, human peripheral blood monocytes (CD14^+^) were isolated for bulk RNA‐seq analysis. Importantly, AVE0991 exhibited a significant inhibitory effect on the NF‐κB and TNF signaling pathways in human monocytes (Figure S21E,F, Supporting Information). Subsequently, the in vivo function of TNFα in *LysM* ^cre^ *Mas1* ^f/f^‐APAP mice was modulated using (Rac)‐Benpyrine (a TNF‐α inhibitor) and R‐7050 (a TNF‐α receptor antagonist). Remarkably, both (Rac)‐Benpyrine and R‐7050 exhibited significant hepatoprotective effects by attenuating liver injury through depletion of the characteristic AILI microenvironment, including MYC^+^ ECs, MMP12^+^ Mψ and CCR2^+^ monocytes (Figure 5G,H and Figure S21G–P, Supporting Information). Taken together, myeloid Mas modulates the AILI microenvironment via the NF‐κB/TNF‐α pathway.

### 2.6 The AILI Microenvironment visualized by DAOSLIMIT Exhibits a Strong Correlation With Advanced Human DILI

**Figure 6:**
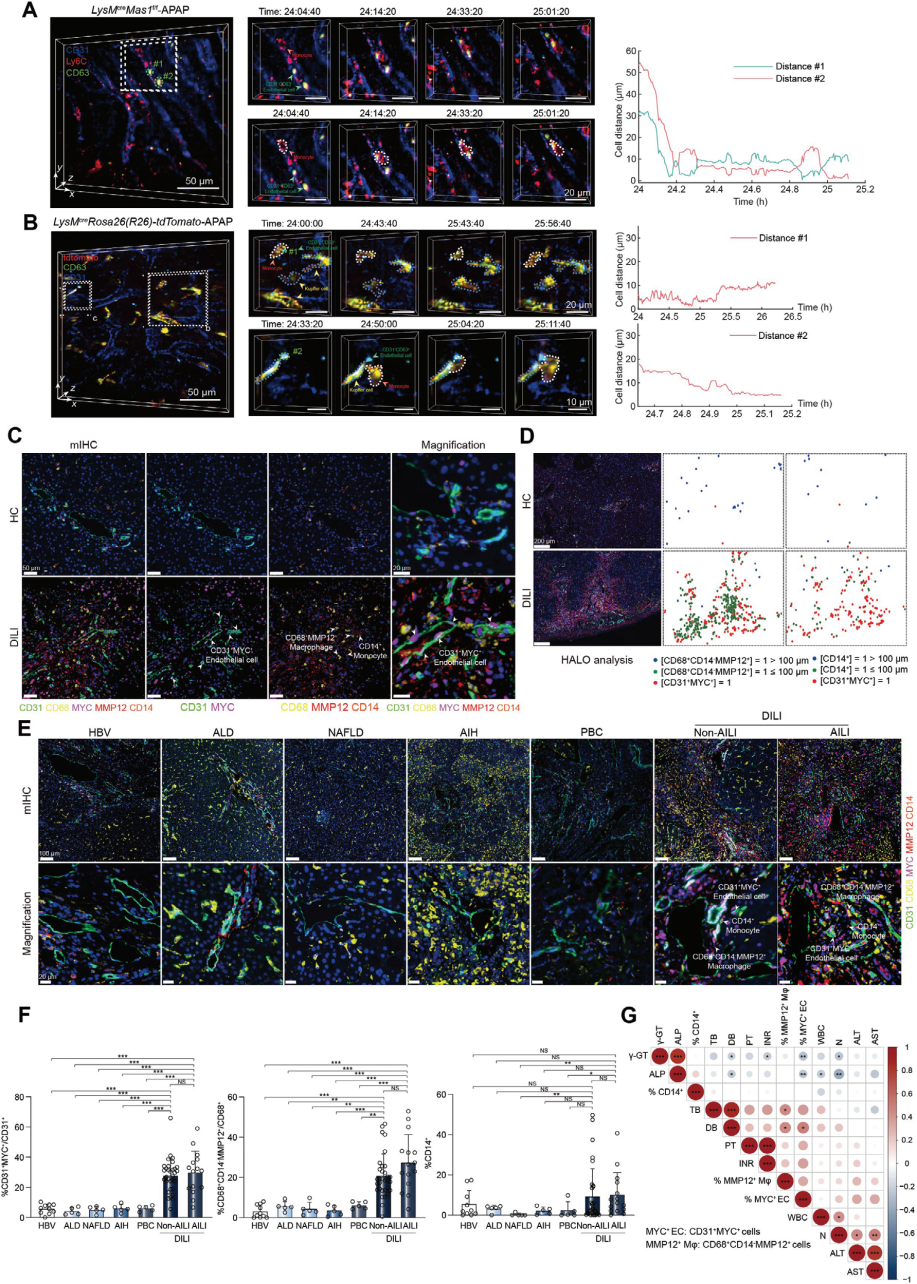
The characteristic AILI microenvironment visualized by DAOSLIMIT in living mice correlates well with human DILI progression. A‐B) Timelapse data captured by DAOSLIMIT showing the interactions between monocytes and CD63^+^ ECs in *LysM* ^cre^ *Mas1* ^f/f^‐APAP mouse (A) and *LysM* ^cre^ *Rosa26(R26)‐tdTomato*‐APAP mouse (B). The curves of cell distances are shown on the right. Scale bar: 50 µm. C) mIHC of CD31^+^MYC^+^ ECs, CD68^+^CD14^−^MMP12^+^ Mψ and CD14^+^ monocytes in the livers of healthy individuals and patients with DILI (*n* = 14 healthy and 47 with DILI). Scale bar: 50 µm and 20 µm. D) Spatial localization of CD31^+^MYC^+^ ECs, CD68^+^CD14^−^MMP12^+^ Mψ and CD14^+^ monocytes within injured region (*n* = 14 healthy and 47 with DILI). Scale bar: 200 µm. E) mIHC of CD31^+^MYC^+^ ECs, CD68^+^CD14^−^MMP12^+^ Mψ and CD14^+^ monocytes in the livers of patients with chronic hepatitis B (CHB), alcoholic liver disease (ALD), nonalcoholic fatty liver disease (NAFLD), autoimmune hepatitis (AIH), primary biliary cholangitis (PBC), Non‐AILI and AILI (*n* = 10 with CHB, 5 with ALD, 5 with NAFLD, 5 with AIH, 5 with PBC, 32 with Non‐AILI and 15 with AILI). Scale bar: 100 µm and 20 µm. F) Quantification of CD31^+^MYC^+^ ECs, CD68^+^CD14^−^MMP12^+^ Mψ and CD14^+^ monocytes for mIHC as shown in E (*n* = 10 with CHB, 5 with ALD, 5 with NAFLD, 5 with AIH, 5 with PBC, 32 with Non‐AILI and 15 with AILI; two‐sided Student's t‐test and two‐sided Mann‐Whitney U test; *p* = 5.00 × 10^−6^, 5.30 × 10^−4^, 5.30 × 10^−4^, 6.24 × 10^−4^, 6.24 × 10^−4^, 1.00 × 10^−5^, 5.00 × 10^−6^, 8.00 × 10^−6^, 1.20 × 10^−5^, 1.30 × 10^−5^, 5.84 × 10^−1^ from left to right for quantification of CD31^+^MYC^+^ ECs; *p* = 9.00 × 10^−6^, 1.01 × 10^−3^, 1.01 × 10^−3^, 8.62 × 10^−4^, 1.18 × 10^−3^, 8.20 × 10^−5^, 2.80 × 10^−5^, 1.70 × 10^−5^, 1.00 × 10^−5^, 3.70 × 10^−5^, 9.13 × 10^−2^ from left to right for quantification of CD68^+^CD14^−^MMP12^+^ Mψ; *p* = 6.79 × 10^−1^, 8.24 × 10^−1^, 6.72 × 10^−3^, 4.77 × 10^−1^, 1.10 × 10^−1^, 2.22 × 10^−1^, 1.90 × 10^−1^, 5.20 × 10^−3^, 8.87 × 10^−2^, 4.46 × 10^−2^, 2.18 × 10^−1^ from left to right for quantification of CD14^+^ monocytes). G) Correlation analysis between CD31^+^MYC^+^ ECs, CD68^+^CD14^−^MMP12^+^ Mψ, CD14^+^ monocytes and DILI pathology parameters (*n* =  47 patients; Pearson correlation; color and circle size indicate correlation). In all graphs data are presented as mean ± SD, *p < 0.05; **p < 0.01; ***p < 0.001; NS, non‐significant.

Cells exhibit different behaviors and exert biological functions across the wide temporal dimension, due to the changing microenvironment and continuous interactions with diverse cells.^[^ ^31^, ^32^ ^]^ Studying the exquisite intercellular behaviors and coordinated functions in multicellular organisms necessitates advanced microscopy with high‐resolution and high‐speed traits, especially in mammals.^[^ ^33^ ^]^ DAOSLIMIT, a recently developed intravital microscopy, fills up the niche in high‐resolution, high‐speed volumetric observations with minimized photon budgets and permits the study of highly motile intercellular interactions of multiple mammalian cells and organelles during their physiological processes.^[^ ^34^, ^35^ ^]^ Thus, in this study, we used DAOSLIMIT to record the real‐time dynamic intercellular interactions within the hepatic tissue of live mice. Extensive evidence of monocytes‐CD63^+^ ECs crosstalk was observed in *LysM* ^cre^ *Mas1* ^f/f^‐APAP mice (**Figure** **6**A and Figure S22A, Supporting Information). Specifically, monocytes exhibiting persistent interactions with CD63^+^ ECs were not removed by blood flow (Videos S5 and S6, Supporting Information). The *LysM* ^cre^Rosa26(R26)‐tdTomato mice were generated to validate the crosstalk between myeloid cells and CD63^+^ ECs in mouse AILI. DAOSLIMIT revealed intimate and dynamic interactions within the AILI microenvironment among CD63^+^ ECs, monocytes, and Kupffer cells (Figure 6B and Video S7, Supporting Information). The clinical relevance of the myeloid‐Mas‐imprinted AILI microenvironment, characterized in mouse models, was further investigated using mIHC to confirm its presence in human diseases. Notably, a highly similar microenvironment consisting of CD31^+^MYC^+^ ECs, CD68^+^CD14^−^MMP12^+^ Mψ, and CD14^+^ monocytes was found to be enriched in DILI patients compared to healthy controls (HCs) (Figure 6C,D). Additionally, in the context of human DILI, CD31^+^MYC^+^ ECs and CD68^+^MMP12^+^ Mψ exhibited significantly elevated levels of key glycolytic enzymes (Figure S22B, Supporting Information); Furthermore, TNFα^+^NF‐κB^+^CD14^+^ monocytes were found to be closely associated with TNFR1^+^CD31^+^MYC^+^ ECs (Figure S22C,D, Supporting Information). Moreover, the localization pattern of CD63+ ECs exhibited a high degree of consistency with that of CD31^+^CD146^+^MYC^+^ ECs (Figure S22E, Supporting Information). Furthermore, mIHC analysis revealed a significantly elevated presence of hepatic MYC^+^ ECs and MMP12^+^ Mψ in cases of human DILI (including AILI and non‐AILI groups) compared to other liver diseases, highlighting the disease‐specific nature of the microenvironment (Figure 6E,F). Moreover, the statistical analysis revealed a significant positive correlation between hepatic CD31^+^MYC^+^ ECs, CD68^+^CD14^−^MMP12^+^ Mψ, and clinical severity parameters of DILI (Figure 6G). Furthermore, the human cell frequency data of the microenvironment were graphed in 3D space, illustrating the distinct mode of DILI compared with other hepatic disorders (Figure S23A, Supporting Information). The k‐means algorithm was also applied to conduct unsupervised clustering, resulting in a prediction accuracy of 83.33% (Figure S23B, Supporting Information). This demonstrates the potential of identifying advanced human DILI based on their characteristic microenvironments.

## 3 Discussion

As the leading cause of mortality due to ALF, AILI imposes a significant public health burden.^[^ ^1^ ^]^ The pathogenic microenvironment plays a crucial role in the progression of advanced AILI. However, there is a significant lack of knowledge regarding the dynamic interplay between various cell types and the activation of complex signaling pathways within the AILI microenvironment.

This study employed a multidimensional approach, including scRNA‐seq, ST, and hour‐long intravital imaging, to characterize the “microenvironment” of mouse AILI models established 24 h after APAP exposure. The deficiency of myeloid‐Mas leads to a significant up‐regulation of the “pro‐inflammatory” TNF and NF‐κB signaling pathways in liver‐infiltrating monocytes, subsequently resulting in the release of TNF‐α and inducing phenotypic reprogramming along with the emergence of MYC^+^CD63^+^ ECs. Further, these ECs can generate copious glycolytic metabolites, particularly lactate, to augment glycolysis in neighboring Mψ and promote their polarization toward a mixed M1/M2‐like phenotype. Additionally, MYC^+^ ECs and MMP12^+^ Mψ recruit a greater number of monocytes to sustain the “characteristic” highly pro‐inflammatory and glycolytic microenvironment. Moreover, the in vivo data established a causal relationship between the activation of myeloid‐Mas and both the attenuated phenotype of mouse AILI and reduced hepatic monocyte count. Notably, the most intuitive intravital evidence of potential cellular crosstalk among CD63^+^ ECs, Ly6C^+^ monocytes, and Kupffer cells within the mouse AILI environment was obtained from the DAOSLIMIT platform. To the best of our knowledge, this is the first study to elucidate the immunometabolic landscape driven by myeloid‐Mas signaling in mouse AILI.

Mas is expressed ubiquitously in human and mouse tissues and is particularly abundant in myeloid cells. By generating multiple cell‐specific *Mas1*‐knockout mice and conducting subsequent scRNA‐seq analyses, we demonstrated the crucial role of myeloid‐Mas. The small‐molecule Mas agonist, AVE0991, exhibits beneficial effects on AILI mice in vivo at various dosages and time points exerting its influence on hepatocyte lipophagy and fatty acid oxidation as previously documented in our research.^[^ ^11^ ^]^ Although AVE0991 did not demonstrate off‐target effects on Mas, it has the potential to target multiple cell types to determine the expression patterns of Mas. The current study provides in vivo and in vitro evidence of the enhanced effect of AVE0991 on the anti‐inflammatory phenotype of myeloid cells. The involvement of Mas in mouse AILI goes beyond its impact on early hepatocyte death; it also influences the pathogenic, pro‐inflammatory microenvironment during the advanced stages (as demonstrated in this study). Therefore, AVE0991 has the potential to target distinct cell populations at different stages of AILI, thereby significantly ameliorating the disease phenotype in AILI mice. Although NAC can alleviate liver injury caused by APAP overdose in the clinic by alleviating oxidative stress, multiple studies strongly support the feasibility of obtaining additional benefits in the treatment of drug‐induced hepatotoxicity through the modulation of hepatocyte‐extrinsic immune mechanisms.^[^ ^36^, ^37^ ^]^ After demonstrating that Mas could be an attractive target for promoting the resolution of inflammation, it may be interesting to integrate NAC with Mas as a combination strategy for antioxidant and anti‐inflammatory activities in the future. A sophisticated combination therapy targeting both inflammation and oxidative damage may prove to be a promising avenue for addressing AILI as well as other liver injuries and diseases. Additionally, considering the central role of Mas in cell injury, metabolism, and inflammatory responses,^[^ ^6^ ^]^ the precise targeting of AVE0991 to specific cells and tissues is a crucial avenue for future investigations. This approach has immense potential to augment or prolong the protective effects mediated by Mas activation in various liver diseases, not just AILI.

After APAP overdose, there is dose‐dependent damage, and the contribution of innate immune cells to APAP toxicity and inflammatory responses has been demonstrated to be dose‐dependent.^[^ ^38^, ^39^ ^]^ Among these, the mechanisms triggered following the administration of 600 mg kg^−1^ APAP are more closely associated with events that contribute to ALF in patients. However, for the exacerbated phenotype and extremely high mortality rate observed in the *Mas1* ^−/−^‐APAP group, we finally chose to pursue further research using APAP at dosage of 300 mg kg^−1^ rather than 600 mg kg^−1^, as previously described studies.^[^ ^40^, ^41^, ^42^, ^43^ ^]^ Although specific inflammatory and repair responses are observed with high‐dose APAP exposure,^[^ ^38^, ^39^ ^]^ the phenotypic exacerbation resulting from *Mas1* knockout can induce these reactions, even at lower dosage levels. This hypothesis was confirmed through our analysis of liver failure samples associated with DILI in humans who were also recipients of transplanted liver. These results have revealed a liver microenvironment that closely resembles the observed characteristics in *Mas1* ^−/−^‐APAP‐300 mice, thereby highlighting the clinical relevance with human liver failure. To enhance the comprehensiveness of our study, we also include data from higher doses of APAP (600 mg kg^−1^) during the early (3 and 6 h) and late stages (24 h) of AILI. Consistently, the myeloid‐Mas signaling deficiency exacerbates high‐dose AILI by modulating the inflammatory microenvironment rather than affecting early cell death. Moreover, the identification of MYC^+^CD63^+^ ECs within the characteristic microenvironment closely aligns with the findings of a recent study that collectively demonstrated the presence of MYC, a common transcriptional program governing monocyte infiltration in ALF. This also validates our findings in this study, which could potentially extend to high‐dose‐induced mouse AILI and even hold significant implications for future therapeutic interventions targeting human ALF.

Angiotensin (Ang)‐(1‐7) activates Mas to exert a protective role in injurious, inflammatory, and metabolic disorders. The beneficial effects of Mas activation have been experimentally documented in various liver diseases other than AILI, including steatohepatitis and hepatic fibrosis. The clinical relevance of Mas is beyond doubt since Ang converting enzyme inhibitors has become one of the most successful therapeutic strategies in patients with hypertension. In this study, the microenvironment identified in *LysM* ^cre^ *Mas1* ^f/f^‐APAP mice can also be observed in human ALF samples associated with DILI, encompassing both AILI and non‐AILI cases, suggesting the broad clinical significance of our study findings. The prediction model, constructed based on our data regarding the number of microenvironmental cell subsets (MYC^+^ ECs, MMP12^+^ macrophages, and monocytes) obtained through mIHC staining, exhibits an accuracy rate of 83.33% in the 3D space. This indicates its ability to effectively discriminate between DILI and non‐DILI liver diseases. However, upon analyzing the characteristics of human liver samples involved in this study, it becomes evident that disease severity, specifically whether acute liver failure has occurred rather than the etiology of liver injury, is the pivotal factor contributing to the emergence of the distinctive microenvironment. In the future, it may be feasible to establish a correlation with all‐cause human ALF. An in‐depth exploration will augment our understanding of the mechanisms underlying ALF and facilitate the identification of potential therapeutic targets.

The pivotal role that metabolism plays in cellular function has been increasingly understood recently. Here, glycolysis was demonstrated to be the key metabolic pathway for maintaining the pathogenic, pro‐inflammatory microenvironment using data from *Cdh5* ^cre^ *Pkm* ^f/+^ mice and systemic administration of 3PO (partially affecting APAP metabolism). These findings imply that modulation of the metabolic state of specific cellular populations represents a potential molecular pathway that governs the pro‐inflammatory microenvironment. Therefore, elucidating the regulatory mechanism of AVE0991 on cellular glucose metabolism within the AILI microenvironment is also a crucial avenue for clinical translation.

Last but not least, this study presented a methodological framework that can serve as a valuable template for future research on microenvironments. scRNA‐seq and ST are revolutionary techniques that promote the study of cell composition, physiology, and cell‐to‐cell interactions in complex microenvironments.^[^ ^44^ ^]^ However, cellular functions and behaviors constitute an intricate microscopic world in which the logistics, plasticity, interactions, and migration of cells and organelles play vital roles in various physiological phenomena at high speeds over the long term,^[^ ^31^ ^]^ beyond the applicability of scRNA‐seq and ST technologies, as well as traditional intravital microscopes. For example, the composition of immune cells in mice undergoes significant changes over time following injury.^[^ ^45^ ^]^ the analysis of static samples cannot reveal the biological mystery concealed in the temporal dimension. By incorporating this paradigm with DAOSLIMIT, a novel intravital microscope that fulfills the unmet requirement for hour‐long, high‐speed 3D subcellular observations in vivo,^[^ ^34^ ^]^ we demonstrated that evaluating the behavior of immune cells in situ is an effective way to uncover complicated immune processes and disease pathogenesis. In the livers of live AILI mice, we observed persistent cellular crosstalk between CD63^+^ ECs and Ly6C^+^ monocytes, which remained unaffected by blood flow, providing valuable insights for subsequent investigations into the microenvironment.

Collectively, myeloid Mas plays a pivotal role in maintaining the unique microenvironment of AILI. These findings significantly enhance our understanding of the progression of AILI and provide multiple cellular and molecular mechanisms for designing novel therapeutic interventions.

## 4 Experimental Section

### Experimental Animals

Male WT C57BL/6J mice aged 6–8 weeks were purchased from SLAC Laboratory Animal Company (Shanghai, China). *Mas1* ^−/−^, *Mas1* ^f/f^, *Pkm* ^f/f^, *Alb*‐cre, *LysM*‐cre, *Lrat*‐cre and *Cd4*‐cre mice were purchased from Cyagen Biosciences Inc (Suzhou, China). *Cdh5*‐cre and *Rosa26*‐LSL‐tdTomato mice were purchased from Shanghai Model Organisms Center, Inc (Shanghai, China). The hepatocyte‐specific *Mas1* knockout (*Alb* ^cre^ *Mas1* ^f/f^) mice were generated by crossing *Mas1* ^f/f^ with *Alb*‐cre mice. The myeloid cell‐specific *Mas1* knockout (*LysM* ^cre^ *Mas1* ^f/f^) mice were generated by crossing *Mas1* ^f/f^ with *LysM*‐cre mice. The endothelial cell‐specific *Mas1* knockout (*Cdh5* ^cre^ *Mas1* ^f/f^) mice were generated by crossing *Mas1* ^f/f^ with *Cdh5*‐cre mice. The hepatic stellate cell‐specific *Mas1* knockout (*Lrat* ^cre^ *Mas1* ^f/f^) mice were generated by crossing *Mas1* ^f/f^ with *Lrat*‐cre mice. The T cell‐specific *Mas1* knockout (*Cd4* ^cre^ *Mas1* ^f/f^) mice were generated by crossing *Mas1* ^f/f^ with *Cd4*‐cre mice. The endothelial cell‐specific *Pkm* knockout (*Cdh5* ^cre^ *Pkm* ^f/+^) mice were generated by crossing *Pkm* ^f/f^ with *Cdh5*‐cre mice. The myeloid cell tracer (*LysM* ^cre^ *Rosa26(R26)*‐tdTomato) mice were generated by crossing *Rosa26*‐LSL‐tdTomato with *LysM*‐cre mice. To develop drug‐induced hepatotoxicity, mice were given intraperitoneal injections of APAP (300 mg kg^−1^) (Cayman) for 24 h, following overnight fasting. Other doses were also tried to determine the optimum parameters of APAP challenge in mice. Unless specified, AVE0991 was administrated prophylactically 2 h before APAP treatment. For further details regarding the materials and methods used, please refer to Table S1 (Supporting Information). All mice were maintained in a specific pathogen‐free facility. Blood and liver samples were collected at indicated time points for further analysis. The experimental protocols were approved by the Animal Ethics Commission of Shanghai Tongji Hospital, Tongji University School of Medicine (2021‐DW‐007).

### 10x Genomics scRNA‐Seq

Single cells from each sample were independently processed into single‐cell suspensions and library generations on a 10× Genomics system. The freshly prepared single‐cell suspension was adjusted to a cell concentration of 700–1200 cells µL^−1^, and the machine and library construction were carried out according to the operation manual of 10× Genomics Chromium Next GEM Single Cell 3ʹ Reagent Kits v3.1 (Catalog No. 1000268). The constructed library was subjected to high‐throughput sequencing using the Illumina Nova 6000 PE150 platform.

### scRNA‐seq Data Preprocessing

The Cell Ranger software pipeline (version 5.0.0) provided by 10×Genomics was used to demultiplex cellular barcodes, map reads to the genome and transcriptome using the STAR aligner, and down‐sample reads as required to generate normalized aggregate data across samples, producing a matrix of gene counts versus cells. We processed the unique molecular identifier (UMI) count matrix using the R package Seurat (version 3.1.1).^[^ ^46^ ^]^ To remove low‐quality cells and likely multiplet captures, which was a major concern in microdroplet‐based experiments, a criterion was applied to filter out cells with UMI gene^−1^ number out of the limit of mean value +/‐ 2 fold of standard deviations by assuming a Gaussian distribution of each cells' UMI gene^−1^ number. Following a visual inspection of the distribution of cells by the fraction of mitochondrial genes expressed, low‐quality cells were further discarded where > 20% of the counts belonged to mitochondrial genes. Additionally, the DoubletFinder package (version 2.0.2) was applied to identify potential doublet.^[^ ^47^ ^]^ After applying these QC criteria, single cells were subjected to downstream analyses. Library size normalization was performed with the NormalizeData function in Seurat to obtain the normalized count. Specifically, the global‐scaling normalization method “LogNormalize” normalized the gene expression measurements for each cell by the total expression, multiplied by a scaling factor (10000 by default), and the results were log‐transformed.

Top variable genes across single cells were identified using the method described in Macosko et al.^[^ ^48^ ^]^ The most variable genes were selected using the FindVariableGenes function (mean. function = FastExpMean, dispersion. function = FastLogVMR) in Seurat. To remove the batch effects in scRNA‐seq, the mutual nearest neighbors presented by Haghverdi et al was performed with the R package Batchelor.^[^ ^49^ ^]^ Graph‐based clustering was performed to cluster cells according to their gene expression profile using the FindClusters function in Seurat. Cells were visualized using a 2D t‐distributed stochastic neighbor embedding algorithm with the RunTSNE function in Seurat. We used the FindAllMarkers function (test.use = bimod) in Seurat to identify marker genes of each cluster. For a given cluster, FindAllMarkers identified positive markers compared with all other cells. Then, the R package SingleR was used,^[^ ^50^ ^]^ a novel computational method for unbiased cell type recognition of scRNA‐seq, with the reference transcriptomic datasets “Mouse Primary Cell Atlas” to infer the cell of origin of each of the single cells independently and identify cell types.^[^ ^51^ ^]^

DEGs were identified using the FindMarkers function (test.use = MAST) in Seurat. *p*‐Value < 0.05 and |log_2_foldchange| > 0.58 was set as the threshold for significantly differential expression. Gene Ontology (GO) enrichment and KEGG pathway enrichment analysis of DEGs were respectively performed using R based on the hypergeometric distribution. The sequencing and bioinformatics analysis were performed by OE Biotech Co., Ltd. (Shanghai, China).

### Pseudotime Analysis

The developmental pseudotime was determined with the Monocle2 package.^[^ ^52^ ^]^ The raw count was first converted from the Seurat object into CellDataSet object with the importCDS function in Monocle. The differential GeneTest function of the Monocle2 package was used to select ordering genes (*q*val < 0.01) which were likely to be informative in the ordering of cells along the pseudotime trajectory. The dimensional reduction clustering analysis was performed with the reduceDimension function, followed by trajectory inference with the orderCells function using default parameters. Gene expression was plotted with the plot_genes_in_pseudotime function to track changes over pseudo‐time.

### Cell‐Cell Communication Analysis

CellPhoneDB (v2.0) was used to identify biologically relevant L‐R interactions from scRNAseq data.^[^ ^53^ ^]^ A ligand or a receptor was defined as “expressed” in a particular cell type if 10% of the cells of that type had non‐zero read counts for the ligand/receptor encoding gene. Statistical significance was then assessed by randomly shuffling the cluster labels of all cells and repeating the above steps, which generated a null distribution for each LR pair in each pairwise comparison between the two cell types. After running 1000 permutations, *p*‐values were calculated with the normal distribution curve generated from the permuted LR pair interaction scores. To define networks of cell‐cell communication, any two cell types were linked where the ligand was expressed in the former cell type and the receptor in the latter. R packages Igraph and Circlize were used to display the cell‐cell communication networks.

### GSVA

To perform the GSVA, the GSEABase package (version 1.44.0) was used to load the gene set file which was downloaded and processed from the KEGG database (https://www.kegg.jp/). To assign pathway activity estimates to individual cells, GSVA was applied with the standard settings,^[^ ^54^ ^]^ as implemented in the GSVA package (version 1.30.0). The differences in pathway activities scored per cell were calculated with LIMMA package (version 3.38.3).

### Tissue Preparation, Cryosectioning, Fixation, Staining and Brightfield Imaging for ST

The tissues obtained from mouse livers were cut into 4–5 mm^3^ pieces and treated with cleanroom wipers. After removing blood stains on the tissue surface, resh tissue was embedded with OCT and then snap frozen at −80 °C. Cryosections were cut at 10 µm thickness and mounted onto the ST arrays. Next, the tissue was dehydrated with isopropanol for 1 min followed by staining with H&E. Slides were mounted in 80% glycerol, and brightfield images were taken on 3D HISTECH Pannoramic MIDI FL, whole‐slide scanner at 40× resolution.

### ST Barcoded Microarray Slide Information

Library preparation slides were purchased from the ST team (https://www.10xgenomics.com/). Each of the spots printed onto the array was 55 µm in diameter and 100 µm from center to center, covering an area of 6.5 × 6.5 mm^2^. Each slide includes 4 Capture Areas, each with ≈5000 unique gene expression spots.

### Tissue Optimization

Tissue sections were placed onto corresponding Capture Areas on the Visium Spatial Tissue Optimization Slide. These sections were fixed, stained, and permeabilized for different lengths of time. mRNA released during permeabilization binds to capture probes on the slide. cDNA was generated using fluorescently labeled nucleotides to visualize synthesized cDNA. Finally, the tissue was enzymatically removed, leaving fluorescently labeled cDNA that might be visualized using fluorescence microscopy to select the optimal permeabilization time. i) Tissue was cryosectioned and placed on Capture Areas on the Visium Spatial Tissue Optimization slide. Tissue sections were then fixed with methanol and stained with H&E. Hematoxylin stains the nuclei of mammalian cells, while Eosin stains the extracellular matrix and cytoplasm. Stained tissue sections were imaged for comparison with final fluorescent images. ii) Tissue sections were permeabilized for varying amounts of time. Each Capture Area captured poly adenylated mRNA from the attached tissue section. A Master Mix containing reverse transcription (RT) reagents and fluorescently labeled nucleotides were added on top of the tissue sections, resulting in fluorescently labeled cDNA. iii) Tissue was enzymatically removed, leaving behind fluorescent cDNA covalently linked to oligonucleotides on the Visium Spatial Tissue Optimization slide. Fluorescent cDNA was visualized under fluorescence imaging conditions and verified using the Visium Imaging Test Slide. H&E and fluorescence images were compared. The permeabilization time that results in maximum fluorescence signal with the lowest signal diffusion was optimal. If the signal was the same at two‐time points, the longer permeabilization time was considered optimal.

### On‐slide Tissue Permeabilization, cDNA Synthesis, Library Construction and Sequencing

Tissue sections placed on these Capture Areas were fixed and stained, permeabilized, and cellular mRNA was captured by the primers on the gene expression spots. All the cDNA generated from mRNA captured by primers on a specific spot share a common Spatial Barcode. Libraries were generated from the cDNA and sequenced and the Spatial Barcodes were used to associate the reads back to the tissue section images for spatial gene expression mapping. i) A Permeabilization Enzyme was used to permeabilize the fixed and stained tissue sections on the slide. The poly‐adenylated mRNA released from the overlying cells was captured by the primers on the spots. RT Master Mix containing reverse transcription reagents was added to the permeabilized tissue sections. Incubation with the reagents produces spatially barcoded, full‐length cDNA from poly‐adenylated mRNA on the slide. ii) Second Strand Mix was added to the tissue sections on the slide to initiate second strand synthesis. This was followed by denaturation and transfer of the cDNA from each Capture Area to a corresponding tube for amplification and library construction. iii) After the transfer of cDNA from the slide, spatially barcoded, full‐length cDNA was amplified via Polymerase Chain Reaction (PCR) to generate sufficient mass for library construction. iv. Enzymatic fragmentation and size selection were used to optimize the cDNA amplicon size. P5, P7, i7, and i5 sample indexes, and TruSeq Read 2 (read 2 primer sequence) were added via End Repair, A‐tailing, Adaptor Ligation, and PCR. The final libraries contain the P5 and P7 primers used in Illumina amplification.

Visium Spatial Gene Expression libraries comprise standard Illumina paired‐end constructs that were flanked with P5/P7, necessary for binding to the Illumina flow cell. TruSeq Read 1 was used for priming and sequencing the 16 bp Spatial Barcode and 12 bp UMI, and TruSeq Read 2 was used for priming and sequencing the cDNA insert. The two 10 bp sample indexes were sequenced in the i5 and i7 read respectively. Calculating sequencing depth requires estimating the approximate Capture Area (%) covered by tissue. This might be performed visually or by using the Visium Manual Alignment Wizard in Loupe Browser for a more accurate measurement. See the examples below for estimating the coverage area visually. Minimum 50 000 read pairs per tissue‐covered spot on Capture Area.

### 4.1 Visium Data Preprocessing

Raw FASTQ files and histology images were processed by sample with the SpaceRanger software version 1.2.0, which uses STAR for genome alignment, against the hg38 reference genome. We processed the UMI count matrix using the R package Seurat and normalized the data by SCTransform to account for variance in sequencing depth across data points.^[^ ^46^ ^]^ Variance in molecular counts per spot could be substantial for spatial datasets, particularly if there were differences in cell density across the tissue. This variance in molecular counts across spots was not just technical in nature but also was dependent on the tissue anatomy. Therefore, all the spots were kept.

We used RCTD and MIA to integrate 10X Visium with scRNA‐seq data to infer the location of cell types and states within a complex tissue.^[^ ^13^, ^15^ ^]^

### RCTD

The RCTD algorithm in the spacexr package was used to resolve cell types from a single spot containing a mixture of cell types, infer the cell type proportions with a maximum‐likelihood estimation, and project them onto a spatial map of cell types. In brief, a “single‐cell reference object” was created from scRNA‐seq data with the Reference function. For each sample, ST data were loaded separately into a “SpatialRNA object” using the SpatialRNA function. Next, an “RCTD object” was created with default parameters for each sample using the single‐cell reference object and SpatialRNA object as inputs. RCTD function with doublet_mode set to “full”, allows for arbitrary number of cell types per spot.

### Intravital Imaging of Mammalian Livers Using DAOSLIMIT

To investigate the subcellular immune and inflammatory responses in living mice following multiple liver injury models, DAOSLIMIT was applied that was a newly developed optical instrument and implemented at Tsinghua University (Beijing, China) for intravital high‐speed, high‐resolution observations across hour‐long durations.^[^ ^34^, ^35^ ^]^

The mice (C57BL/6J, male, 6–8 weeks) and transgenic mice (C57BL/6J, male, 6–8 weeks) were medically modeled as indicated in figures and then used for DAOSLIMIT imaging. For the experimental preparations in Figure 4G, Figure S1E, S14G and Videos S1, S3 and S4 (Supporting Information), WGA (5 µg, W11261, Thermo Fisher), Ly6C (3 µg, 128007, Biolegend), Ly6G (3 µg, 127610, Biolegend) and phosphate buffer saline (60 µL, PBS) were injected into mice through the tail vein. For the experimental preparations in Figure 2H, Figure S18H and Video S2 (Supporting Information), CD31 (3 µg, 102423, Biolegend), CD80 (5 µg, 104705, Biolegend), F4/80 (1 µg, 123109, Biolegend), CD163 (2 µg, 155305, Biolegend) and PBS (60 µL) were injected into mice through the tail vein. For the experimental preparations in Figure 1H, 6A, Figure S22A and Videos S5 and S6 (Supporting Information), CD31 (5 µg, 102416, Biolegend), Ly6C (10 µg, NB100‐65413AF594, Novus), CD63 (4 µg, 143920, Biolegend) and PBS (60 µL) were injected into mice through the tail vein. For the experimental preparations in Figure 6B and Video S7 (Supporting Information), CD31 (5 µg, 102416, Biolegend), CD63 (4 µg, 143920, Biolegend), and PBS (60 µL) were injected into the transgenic mice through the tail vein. 10–15 min after the intravenous injection, each mouse was anesthetized with Avertin (350 mg kg^−1^, i.p.) and dissected to expose its liver on a customized sample holder with a 170‐µm‐thick coverslip for intravital imaging. During DAOSLIMIT imaging, a 37 °C body temperature maintenance instrument was used to maintain the mice in their native physiological processes. The ethical approval was procured from the Institutional Animal Care and Use Committee of Tsinghua University.

For data acquisition, *sLFdriver* software was used to acquire DAOSLIMIT data in randomly selected regions of living livers.^[^ ^35^ ^]^ In this project, DAOSLIMIT adopted 3 × 3 scanning mode and was configurated with a 63×/1.4 NA oil‐immersion objective covering the field of view of ≈200 µm × 200 µm × 15 µm. Multi‐color fluorescent photons were excited by rapidly switching the lasers with wavelengths of 405, 488, 561, and 640 nm during the acquisition. The power intensity of lasers was set to 1.6 mW mm^−2^ in 405 nm, 1.2 mW mm^−2^ in 488 nm, 1.0 mW mm^−2^ in 561 nm and 2.4 mW mm^−2^ in 640 nm with the exposure time of 2 s and the interval time of 18 s or 28 s, to avoid photodamage and phototoxicity. For 3D image reconstruction, phase‐space deconvolution was applied to the time‐lapse spatial‐angular data after pixel realignments to generate high‐resolution volumes over time.^[^ ^55^, ^56^ ^]^ Three NVIDIA GeForce RTX 3090 graphics processing units (GPUs) were used to accelerate the reconstruction.

Data analysis and renderings were conducted with our customized MATLAB scripts (MathWorks, MATLAB 2018b), FIJI, and Amira (Thermo Fisher Scientific, Amira 2021).^[^ ^57^ ^]^ The 3D rendering and maximum intensity projection (MIP) of DAOSLIMIT data were carried out by Amira software. The cell identification and counting were accomplished automatically frame by frame on the channel of immune cells using the Particle Analysis plugins in FIJI. The segmentation of monocytes was done with binarization and threshold interpolation, and then centroid distances between monocytes and CD63^+^ ECs were calculated using our customized MATLAB script.

### K‐means Clustering Algorithm

For quantification of % CD31^+^MYC^+^/CD31^+^, % CD14^+^ and % CD68^+^CD14^−^MMP12^+^/CD68^+^, 3–16 randomly selected fields per patient were averaged (*n* = 47 patients with DILI, *n* = 5 patients with AIH, *n* = 5 patients with PBC, *n* = 10 patients with HBV and *n* = 5 patients with ALD). We first plotted the data from all patients in a 3D space, where the three axes represent % CD31^+^MYC^+^/CD31^+^, % CD14^+^, and % CD68^+^CD14^−^MMP12^+^/CD68^+^, respectively. Then, the k‐means algorithm was applied to conduct an unsupervised clustering,^[^ ^58^ ^]^ in which the number of clusters was set to 2. The accuracy was calculated as the ratio of the number of correctly predicted samples to the total sample number. The clustering was carried out with our customized Python scripts (Python 3.11.2).

### mIHC

mIHC was performed as previously described.^[^ ^11^ ^]^ Briefly, the 4‐µm‐thick formalin‐fixed, paraffin‐embedded whole tissue sections were stained with standard, primary antibodies sequentially and paired with TSA 7‐color kit (D110071‐50T, WiSee Bio), then by staining with DAPI. For example, deparaffinized slides were incubated with an anti‐MMP12 antibody (#MA5‐32011, Invitrogen) for 30 min and then treated with anti‐rabbit horseradish peroxidase‐conjugated secondary antibody (#A10011‐60, Yuanxibio) for 10 min. IF labeling was developed for a strictly observed 10 min using TSA 620 per the manufacturer's direction. Slides were washed in buffer and then transferred to preheated citrate solution (90 °C) before being heat‐treated using a microwave set at 20% of maximum power for 15 min. Slides were cooled in the previous solution to room temperature. Between all steps, the slides were washed with buffer. The same process was repeated for the following antibodies/fluorescent dyes, in order: anti‐CD31 (#77699, CST)/TSA 520, anti‐MYC (#32072, abcam)/TSA 670, anti‐CCR2 (#ab273050, Abcam)/TSA 440, anti‐F4/80 (#70076, CST)/TSA 570. Each slide was then treated with 2 drops of DAPI, washed in distilled water, and manually coverslipped. Slides were air‐dried and mounted with an Anti‐fade mounting medium, and pictures were taken with the Aperio Versa 8 tissue imaging system (Leica). Images were analyzed using Indica Halo software.

### Human Samples

Liver samples were collected from patients with DILI who underwent liver transplantation (*n* = 41) at Shanghai Renji Hospital of Shanghai Jiao Tong University (China), and who underwent liver biopsy (*n* = 6) at Shanghai Tongji Hospital of Tongji University (China); and HCs (*n* = 14) who were liver donors. Liver samples from patients with AIH (*n* = 5), PBC (*n* = 5), HBV (*n* = 10), NAFLD (*n* = 5), and ALD (*n* = 5) were all collected from Shanghai Renji Hospital of Shanghai Jiao Tong University (China). The informed consent was obtained from each subject. The study was carried out under the principles of the Declaration of Helsinki and approved by the research ethics boards of Shanghai Renji Hospital and Shanghai Tongji Hospital (KY2021‐063‐B and 2021‐008‐SK). Demographic and clinical features of the enrolled subjects are shown in Table S2 (Supporting Information).

### RNA‐seq Analysis

Bulk RNA‐seq analysis was performed as previously described.^[^ ^11^ ^]^ Briefly, total RNA was extracted using the TRIzol reagent (Invitrogen), with purity and quantification evaluated by NanoDrop 2000 spectrophotometer (Thermo Scientific, USA). The library products were constructed and sequenced on an Illumina HiSeq X Ten platform. FPKM of each gene was calculated using Cufflinks, and the read counts of each gene were obtained by HTSeq‐count. Differential expression analysis was performed using the DESeq (2012) R package. *P* value < 0.05 was set as the threshold for significantly differential expression. Hierarchical cluster analysis of DEGs was performed to demonstrate the expression pattern of genes. GO term and KEGG pathway enrichment analysis of DEGs were performed respectively using R based on the hypergeometric distribution. The transcriptome sequencing and analysis were conducted by OE Biotech (Shanghai, China).

### Flow Cytometry

For flow cytometry analysis, the liver tissue was excised and ground into single‐cell suspension after digestion with collagenase IV and pronase E at 37 °C for 30 min. Then the single‐cell suspension was further purified by using density gradient centrifugation. Collected cells were washed, centrifuged, and resuspended in FACS buffer. Then the cells incubated in FACS buffer (100 µL) containing Fc block (2 µL, 553141, BD Biosciences) for 15 min at room temperature, and then stained with BV510 L/D (564406, BD Biosciences), APC‐Cy7 CD45 (557659, BD Biosciences), PE‐Cy7 CD11b (552850, BD Biosciences), BV421 CD31 (102423, BioLegend), FITC CD63 (143919, BioLegend), PE F4/80 (565410, BD Biosciences), BV421 Ly‐6C (562727, BD Biosciences), FITC CD86 (105005, BioLegend) for 30 min at 4 °C. For staining intracellular antigens, after washing, cells were fixed and permeabilized using a Fixation/Permeabilization Kit (554714, BD Biosciences), and then stained with AF647 CD206 (565250, BD Biosciences) for 30 min at 4 °C. Then the cells were washed with FACS buffer (1 mL), centrifuged at 300 g for 5 min at 4 °C, and resuspended in FACS buffer (0.5 mL) and analyzed using Celesta Cell Analyzer (BD Biosciences) and FlowJo (TreeStar). The compensation and gates were set using isotype controls, single staining, and full staining.

### Isolation of Primary Liver Cells

Primary liver cells were isolated by in situ perfusion of the liver as described previously.^[^ ^11^ ^]^ Then the cell suspension was filtered through a 70 µm cell strainer, and non‐hepatocytes were gathered by using density gradient centrifugation. Primary Kupffer cells were further isolated using Anti‐F4/80‐MicroBeads (130‐110‐443, Miltenyi Biotec). IF staining of F4/80 was used to identify Kupffer cells and the Kupffer cells were cultured in high‐glucose dulbecco's modified eagle medium (DMEM) solution containing 10% fetal bovine serum (FBS) with 100 U mL^−1^ penicillin G and 100 U mL^−1^ streptomycin sulfate at 37 °C with 5% CO_2._ Primary LSECs were isolated using Anti‐CD146‐MicroBeads (130‐092‐007, Miltenyi Biotec). IF staining of CD31 was used to identify LSECs, and LSECs were cultured in mouse LSEC complete medium (CM‐M040, Procell Life Science & Technology Co., Ltd.) at 37 °C with 5% CO_2_ on collagen I‐coated plates.

### BMDMs Isolation and Treatment

Freshly isolated femurs and tibias from 8‐week‐old male WT or *Mas1* ^−/−^ mice were flushed with DMEM. The bone marrow was flushed out with ice‐cold DMEM (10 mL) using a sterile syringe with a needle. The bone marrow suspension was filtered through a 70 µm cell strainer, and then centrifugated at 500 g for 10 min at 4 °C. The acquired cells were plated and cultured in DMEM, supplemented with 10% FBS, 100 U mL^−1^ penicillin G, 100 U mL^−1^ streptomycin sulfate and 100 ng mL^−1^ recombinant mouse M‐CSF (PeproTech) for 7 days to induce Mψ differentiation. On day 7, mature BMDMs were collected for further experiments. For the stimulation of BMDMs, 100 ng mL^−1^ LPS was used for 12 hours to drive proinflammatory activation. Activated BMDMs were collected for RNA‐seq, WB, and IF analyses.

### Preparation of LSEC‐CM

Primary LSECs were isolated from *Mas1* ^−/−^‐APAP mice and cultured in mouse LSEC complete medium (CM‐M040, Procell Life Science & Technology Co., Ltd.) at 37 °C with 5% CO_2_ on collagen I‐coated plates. Supernatants were collected on day 2. CM was transferred to a 50 mL BD Falcon tube and centrifuged at 1500 r.p.m. for 10 min to remove cellular debris. The CM was sterile filtered using a 0.22‐µm filter and stored in −80 °C atmosphere before being co‐cultured with Kupffer cells. The mouse LSEC complete medium not exposed to cultured cells was also prepared as above and served as the control medium.

### Preparation of BMDM‐CM

Mature BMDMs were stimulated with 100 ng mL^−1^ LPS for 12 h, then the CM was aspirated, and the cells were rinsed thoroughly and cultured with fresh growth medium for an additional 24 h. CM was transferred, centrifuged, sterile filtered (0.22 µm filter), and stored in −80 °C atmosphere before co‐culture with LSECs. BMDM‐CM was equivalently diluted in mouse LSEC complete medium. BMDM‐free medium with the same composition and conditions served as the control medium.

### Serum Biochemistry

Serum ALT activities were measured using a commercial kit (Nanjing Jiancheng, China) according to the manufacturer's instructions.

### Elisa Measurement

TNFα levels in culture supernatants of BMDMs were measured by using a commercial mouse TNFα ELISA kit (Abs520010, Absin) according to the manufacturer's instructions.

### Human Blood Samples

Blood samples were drawn from healthy donors recruited at Taihe Hospital (Changsha, China). The study was approved by the Institutional Review Board of the Taihe Hospital and was carried out according to local guidelines and regulations (JH‐WBC‐20220217). The healthy donors went through the following screening panel and were found to be negative for sterile detection (bacterial, fungi, and mycoplasma) and exogenous virus detection (hepatitis B surface antigen, hepatitis C virus, human immunodeficiency virus, and treponema pallidum). Before donor inclusion, written informed consent was obtained. CD14^+^ monocytes were selected via immunomagnetic separation from peripheral blood mononuclear cells (PBMCs). After isolation, the CD14^+^ monocytes were tested for purity and viability by flow cytometry. The CD14^+^ monocytes were resuspended in Dulbecco's phosphate‐buffered saline with 0.5% bovine serum albumin and 2 mm ethylenediaminetetraacetic acid, and stored at the temperature of 2–8 °C for further experiments. Briefly, ficoll (5 mL) was added to a 15 mL centrifuge tube, then the blood sample was slowly added to the upper layer of the separation solution, followed by the full centrifugation at 600 g for 18 min. After centrifugation, the middle layer lymphocytes were extracted into another centrifuge tube, then magnetic‐activated cell sorting (MACS) buffer was added to wash once and then centrifuged at 300 g for 10 min. After centrifugation, the supernatant was removed, and the cell pellet was resuspended to 10 mL. According to the counting results, 1 × 10^8^ PBMCs were taken for magnetic bead sorting. The 1 × 10^8^ PBMCs were centrifuged at 300 g for 10 min. After centrifugation, the supernatant was removed, and the cell pellet was resuspended with MACS buffer (800 uL) and then added CD14 magnetic beads (200 uL, Miltenyi, 130‐050‐201). The mixture was incubated at 4 °C for 15 min. After incubation, MACS buffer was added to 14 mL and centrifuged at 300 g for 10 min. After centrifugation, the supernatant was removed and the cell pellet was resuspended with MACS buffer (500 uL). Then the LS separation column was put on the magnetic stand and buffer (3 mL) was added to rinse the separation column. After rinsing, the cell suspension was added to the separation column until exhaustion, and then buffer (3 mL) was added to wash the separation column. After the washing was completed, the washing step was repeated twice. Then the sorting column was removed and buffer (5 mL) was used to press the cells bound on the sorting column into a 15 mL centrifuge tube. 100 uL of cell suspension was taken for counting. CD45 (2 uL) and CD14 (2 uL) antibodies were added to the cell suspension, which was then incubated at 4 °C for 15 min, followed by the addition of PI (2 uL). After mixing, the positive rate was detected by flow cytometry. The qualified CD14^+^ monocytes were stored for further experiments.

### IF

For IF, the BMDM were incubated with NF‐κB p65 (1:400, #8242S, CST), F4/80 (1:50, ab90247, abcam), TNFα (1:100, ab1793, abcam) and DAPI (1:3000, 2879038, Peprotech). Images were acquired using a confocal microscope (Zeiss LSM800).

### WB Analysis

Extracted proteins were quantified as previously described.^[^ ^11^ ^]^ PhosSTOP (Roche) was added to detect the phosphorylated proteins. Proteins were assessed with the following primary antibodies (Abs). Abs to CYP2E1 (55 kDa, ab28146), RIP (75 kDa, ab202985), and TNFα (26 kDa, ab215188) were purchased from Abcam (MA, USA). Abs to NF‐κB p65 (65 kDa, #8242S) and p‐NF‐κB p65 (65 kDa, #3033S) were purchased from CST (MA, USA). The protein levels were normalized using the anti‐β‐actin antibody (45 kDa, #8457) purchased from CST (MA, USA).

### Glycolytic Rate Assay

The Agilent Seahorse XF Glycolytic Rate Assay was an accurate and reliable analytical method for measuring glycolysis in cells. Seahorse XF Glycolytic Rate Assay provides accurate measurements of glycolytic rates for basal conditions and compensatory glycolysis following mitochondrial inhibition. The calculated rates account for the contribution of CO_2_ to extracellular acidification derived from mitochondrial/TCA cycle activity and were directly comparable to lactate accumulation data. Seahorse XF Analyzers directly measure the real‐time extracellular acidification rate (ECAR) and oxygen consumption rate (OCR) of cells, which were the indicators of the two major energy‐producing pathways: glycolysis and oxidative phosphorylation. Most cells possess the ability to switch between these two pathways, thereby adapting to changes in their environment. To measure glycolytic rates, Seahorse XF Glycolytic Rate Assay utilizes both ECAR and OCR measurements to determine the glycolytic proton efflux rate (glycoPER) of the cells. The assay workflow was as follows: first, cells were incubated in the Seahorse XF Glycolytic Rate Assay Medium containing substrates such as glucose, glutamine, and pyruvate, as well as HEPES buffer, and basal rates were recorded over three measurement periods. Next, Rot/AA (Rotenone plus Antimycin A, inhibitors of mitochondrial electron transport chain) were injected to inhibit mitochondrial oxygen consumption (and therefore CO_2_‐derived protons). The second injection was 2‐deoxy‐D‐glucose, a glucose analog that inhibits glycolysis through competitive binding of glucose hexokinase, the first enzyme in the glycolytic pathway. The resulting decrease in PER provides qualitative confirmation that the PER produced prior to the injection was primarily due to glycolysis.

### Prediction Model for Human DILI using Microenvironmental Cell Frequency Data

As shown in Figure S23 (Supporting Information), (*% CD14^+^, % CD31^+^MYC^+^/CD31^+^, % CD68^+^CD14^−^MMP12^+^/CD68^+^*) was assumed to represent the coordinates in the 3D space. The K‐means clustering centroid of DILI group was (32.36, 26.74, 11.72) and the centroid of non‐DILI group was (9.08, 6.68, 3.43). The data closer to the centroid would be automatically clustered into that group. And in other formats, the interface plane was described as the formula of *% CD14^+^* +2.81×*% CD31^+^MYC^+^/CD31^+^* +2.42×*CD68^+^CD14^−^MMP12^+^/CD68^+^* = 106.12. The prediction model could be represented as:

The patient was with DILI, if his/her microenvironmental cell frequency data meets the condition of % CD14^+^+2.81×% CD31^+^MYC^+^/CD31^+^+2.42×CD68^+^CD14^−^MMP12^+^/CD68^+^>106.12,

The patient was not with DILI, if his/her microenvironmental cell frequency data meets the condition of % CD14^+^+2.81×% CD31^+^MYC^+^/CD31^+^+2.42×CD68^+^CD14^−^MMP12^+^/CD68^+^<106.12.

The model had been verified with an accuracy of 83.33%.

### Statistical Analysis

Experimental data were presented as mean ± SD. Statistical analyses were performed by using Prism GraphPad version 7.0 and SPSS version 22.0. Data normality was determined using the Histogram and Shapiro‐Wilk tests. For comparison between the two groups, normally distributed data was analyzed by a two‐sided Student t‐test; otherwise, data was analyzed by a two‐sided Mann‐Whitney U test. For comparison between multiple groups, One‐way ANOVA with Tukey's test was generally applied. Detailed sample size and *P* value were shown in the corresponding figure legends. Statistical significance was considered as *p* < 0.05.

## Conflict of Interest

The authors declare no conflict of interest.

## Author Contributions

S.C., Z.L., Y.Z., and L.X. contributed equally to this work. Conception and design: S.C., J.L., F.W., and C.Y. Acquisition of data: S.C., Z.L., Y.Z., L.X., S.Z., H.J., B.Y., C.L., S.Z., Z.W., S.L. and M.J. Analysis and interpretation of data: S.C., Z.L., and Y.Z. Drafting of the article: S.C., Z.L., J.L. Administrative, technical, or material support: C.L., Z.L. Study supervision: C.Y., F.W., and J.L.

## Supporting information

Supporting Information

Supplemental Video 1

Supplemental Video 2

Supplemental Video 3

Supplemental Video 4

Supplemental Video 5

Supplemental Video 6

Supplemental Video 7

## Data Availability

The data that support the findings of this study are available from the corresponding author upon reasonable request.
