# Supplementary material for: Myeloid‐Mas Signaling Modulates Pathogenic Crosstalk among MYC+CD63+ Endothelial Cells, MMP12+ Macrophages, and Monocytes in Acetaminophen‐Induced Liver Injury
Source: Adv Sci (Weinh). 2024 Feb 13;11(16):2306066. doi: 10.1002/advs.202306066 (PMC11040347; doi:10.1002/advs.202306066)
Supplement: Supplementary file 1 — Supporting Information [file ADVS-11-2306066-s008.pdf]

ADVANCED SCIENCE
Open Access

Supporting Information

for *Adv. Sci.*, DOI 10.1002/advs.202306066

Myeloid-Mas Signaling Modulates Pathogenic Crosstalk among MYC^+CD63^+ Endothelial Cells, $MMP12^+$ Macrophages, and Monocytes in Acetaminophen-Induced Liver Injury

Shuai Chen, Zhi Lu, Yudong Zhao, Lu Xia, Chun Liu, Siqing Zuo, Manchang Jin, Haoyu Jia, Shanshan Li, Shuo Zhang, Bo Yang, Zhijing Wang, Jing Li, Fei Wang* and Changqing Yang**

Myeloid-Mas Signaling Modulates Pathogenic Crosstalk among MYC⁺CD63⁺ Endothelial Cells, MMP12⁺ Macrophages and Monocytes in Acetaminophen-Induced Liver Injury

Shuai Chen, Zhi Lu, Yudong Zhao, Lu Xia, Chun Liu, Siqing Zuo, Manchang Jin, Haoyu Jia, Shanshan Li, Shuo Zhang, Bo Yang, Zhijing Wang, Jing Li, Fei Wang*, Changqing Yang**

Table of contents

Supplementary Figures and Figure legends....3

Figure S1....3
Figure S2....5
Figure S3....7
Figure S4....9
Figure S5....10
Figure S6....11
Figure S7....12
Figure S8....13
Figure S9....15
Figure S10....18
Figure S11....20
Figure S12....22
Figure S13....24
Figure S14....25
Figure S15....29
Figure S16....30
Figure S17....32
Figure S18....34
Figure S19....35
Figure S20....36

Figure S21..38
Figure S22..41
Figure S23..44

Supplementary Tables..45

Table S1...45

Table S2...46

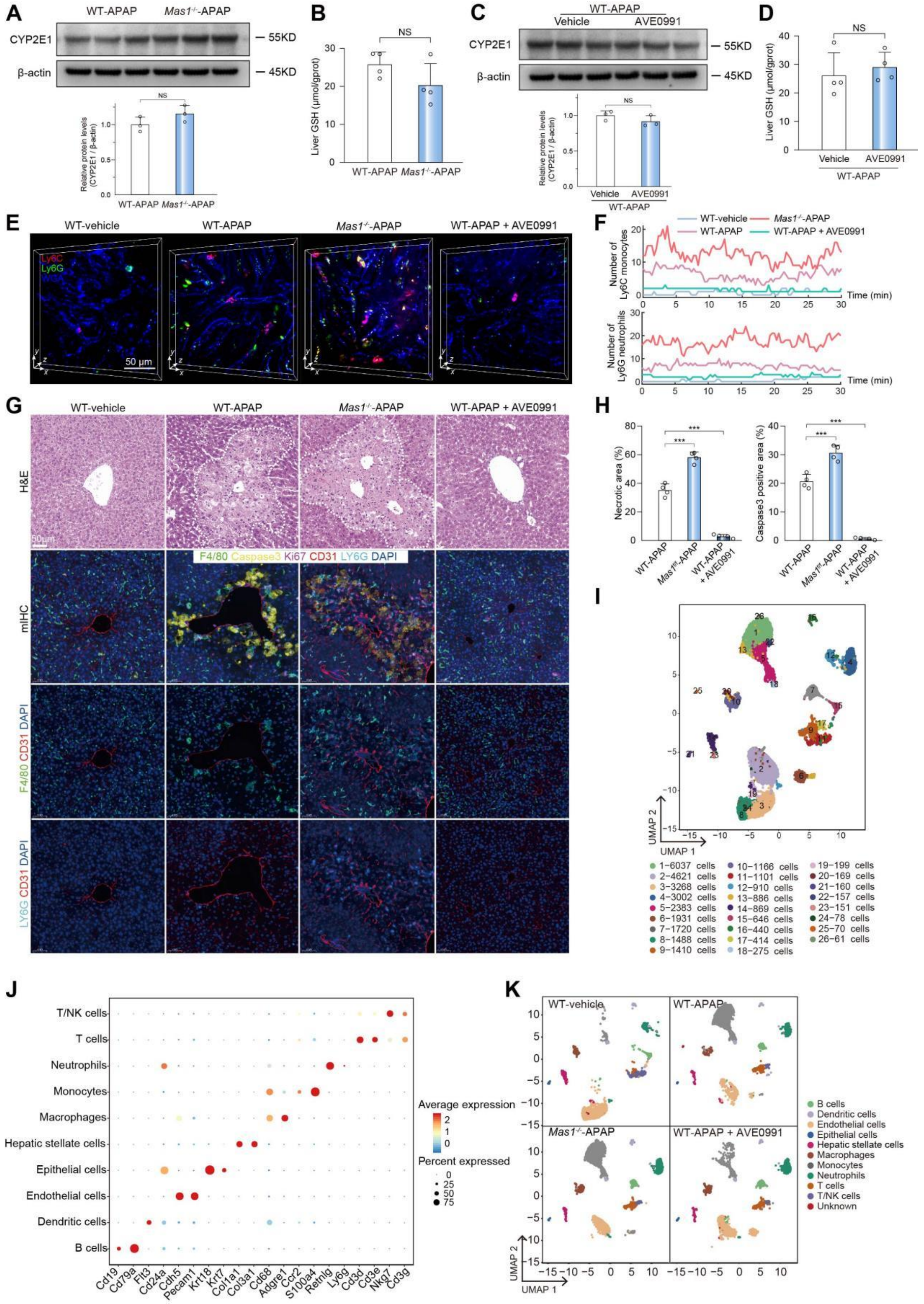

Figure S1. Mas modulates cellular infiltration and resident population response during AILI. A) WT and *Mas1*$^{-/-}$ mice were challenged with APAP for 24 h. Representative liver immunoblots and quantification of CYP2E1 and β-actin (*n* = 3 mice per group; two-sided Student's t-test; *P* = 1.68x10^{-1}). B) WT

and *Mas1*$^{-/-}$ mice were challenged with APAP for 24 h. Hepatic levels of GSH (n = 4 mice per group; two-sided Student's t-test; P = 1.47×10^{-1}). C) Mice were pre-treated with or without AVE0991 for 2 h before APAP challenge for 24 h. Representative liver immunoblots and quantification of CYP2E1 and β-actin (n = 3 mice per group; two-sided Student's t-test; P = 2.36×10^{-1}). D) Mice were pre-treated with or without AVE0991 for 2 h before APAP challenge for 24 h. Hepatic levels of GSH (n = 4 mice per group; two-sided Student's t-test; P = 5.60×10^{-1}). In E-K, mice were pre-treated with or without AVE0991 for 2 h before APAP or saline challenge for 24 h (n = 4 mice per group). E-F) Timelapse data of neutrophils (Ly6G) and monocytes (Ly6C) in the vessels (WGA) of living mouse livers were captured by DAOSLIMIT. Representative intravital images and the temporal traces of their number are shown. Scale bar: 50 μm. G) Representative stainings of H&E and mIHC. Scale bar: 50 μm. H) Quantification of necrotic area for H&E as shown in G (n = 4 mice per group; One-way ANOVA with Tukey's test, P = 1.70×10^{-5} and 9.90×10^{-7} from left to right). Quantification of caspase3 positive area for mIHC as shown in G (n = 4 mice per group; One-way ANOVA with Tukey's test, P = 2.47×10^{-4} and 8.34×10^{-7} from left to right). I) The UMAP plot of 33,612 single cells from liver samples. J) Dot plot showing the expression levels of marker genes in each cell type. K) The UMAP plot showing the differences of intrahepatic main cell types between different groups. In all graphs data are presented as mean ± SD, ***P < 0.001, NS, not significant. APAP, acetaminophen; H&E, hematoxylin-eosin; mIHC, multiplex immunohistochemistry; UMAP, uniform manifold approximation and projection.

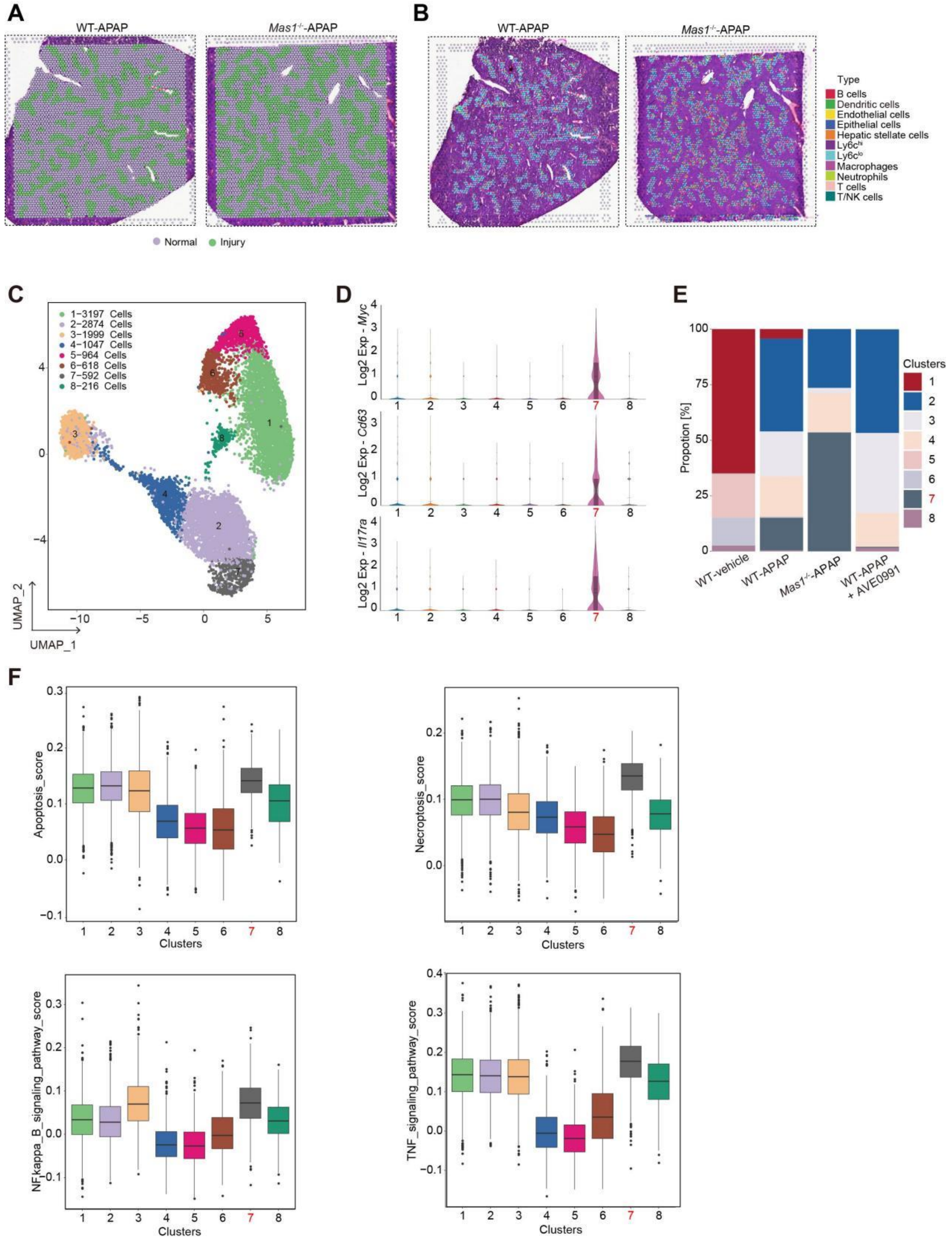

Figure S2. The spatial features of intrahepatic main cell types and the characteristics of the pro-inflammatory EC subgroup. A) ST-defined regions including injured regions and normal regions. B) The Robust Cell Type Decomposition (RCTD) algorithm in the spacexr package was used to resolve cell types from a single spot containing a mixture of cell types. C) The UMAP plot of 11,507 single cells from ECs. D) Violin plots showing marker genes of

EC7 cluster. E) Histogram showing the proportion of each cluster between different groups. F) Box plots showing the score of indicated pathway for EC clusters. APAP, acetaminophen; UMAP, uniform manifold approximation and projection; Exp, expression.

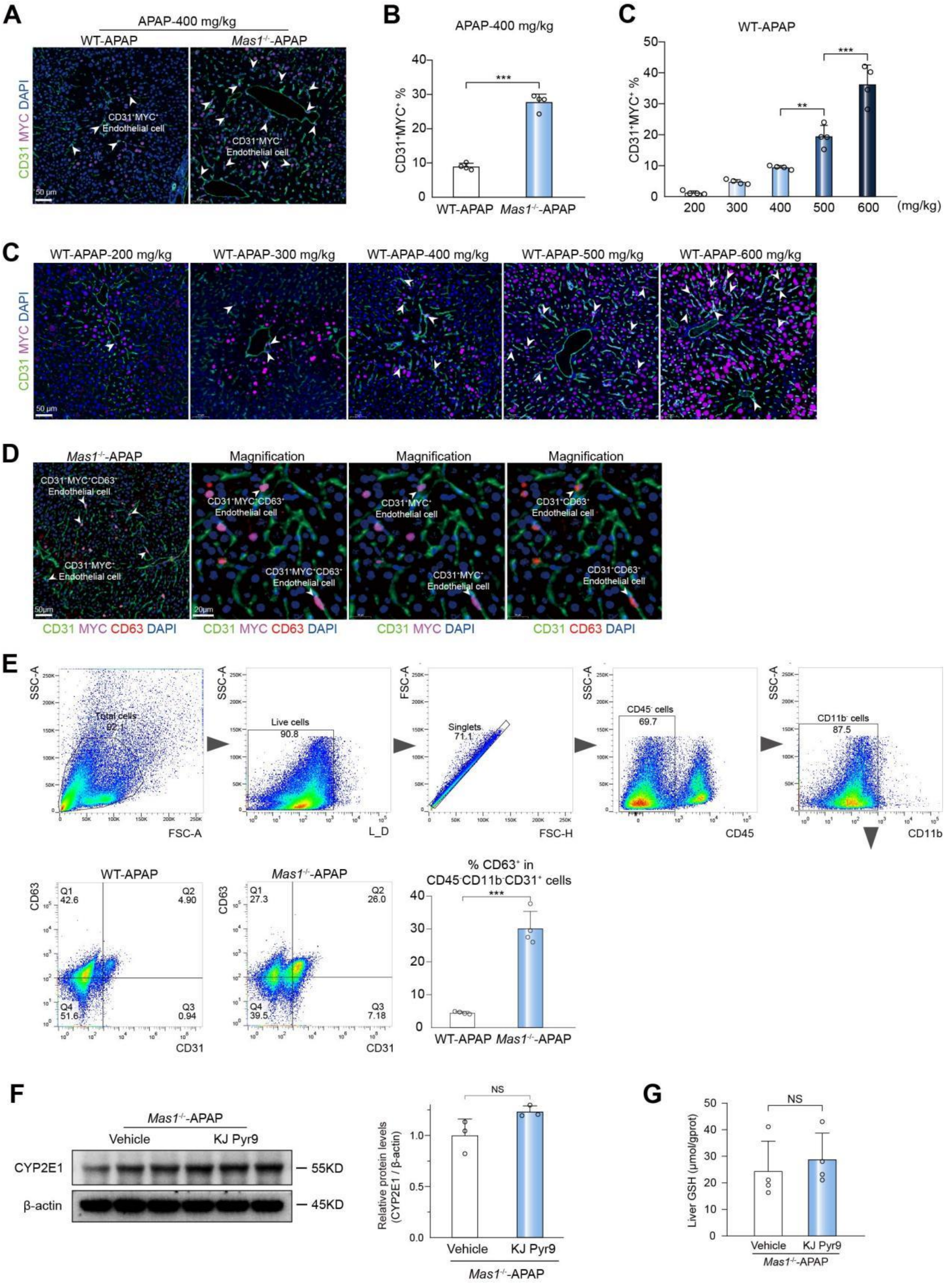

Figure S3. Quantitative changes of the pro-inflammatory EC subgroup during AILI. A) WT and *Mas1*$^{-/-}$ mice were challenged with APAP (400 mg kg^{-1}) for 24 h (n = 4 mice per group). mIHC of CD31^{+}MYC^{+} ECs are shown. Scale bar: 50 μm. B) Quantification of CD31^{+}MYC^{+} ECs for mIHC as shown in A (n = 4 mice per group; two-sided Student's t-test, P = 6.00x10^{-5}). C) WT mice were

challenged with different dose of APAP (200 mg kg^{-1}, 300 mg kg^{-1}, 400 mg kg^{-1}, 500 mg kg^{-1} and 600 mg kg^{-1}) for 24 h (*n* = 4 mice per group). mIHC of $CD31^{+}MYC^{+}$ ECs are shown. Scale bar: 50 μm. Quantification of $CD31^{+}MYC^{+}$ ECs for mIHC are shown (*n* = 4 mice per group; One-way ANOVA with Tukey's test, P = 5.71x10^{-1}, 2.94x10^{-1}, 4.44x10^{-3} and 2.20x10^{-5} from left to right). D) *Mas1*$^{-/-}$ mice were challenged with APAP for 24 h (*n* = 4 mice per group). mIHC of $CD31^{+}MYC^{+}CD63^{+}$ ECs are shown. Scale bar: 50 μm and 20 μm. E) WT and *Mas1*$^{-/-}$ mice were challenged with APAP for 24 h (*n* = 4 mice per group). Representative flow cytometric plots of hepatic $CD45^{-}CD11b^{-}CD31^{+}$ cells showed the difference of $CD63^{+}$ fractions between groups. Quantification of $CD63^{+}$ fractions are shown (*n* = 4 mice per group; two-sided Student's t-test, P = 6.40x10^{-5}). F) Representative liver immunoblots and quantification of CYP2E1 and β-actin (*n* = 3 mice per group; two-sided Student's t-test, P = 7.65x10^{-2}). G) Hepatic levels of GSH (*n* = 4 mice per group, two-sided Student's t-test, P = 5.79x10^{-1}). In all graphs data are presented as mean ± SD, NS, not significant; **P < 0.01; ***P < 0.001. APAP, acetaminophen; mIHC, multiplex immunohistochemistry.

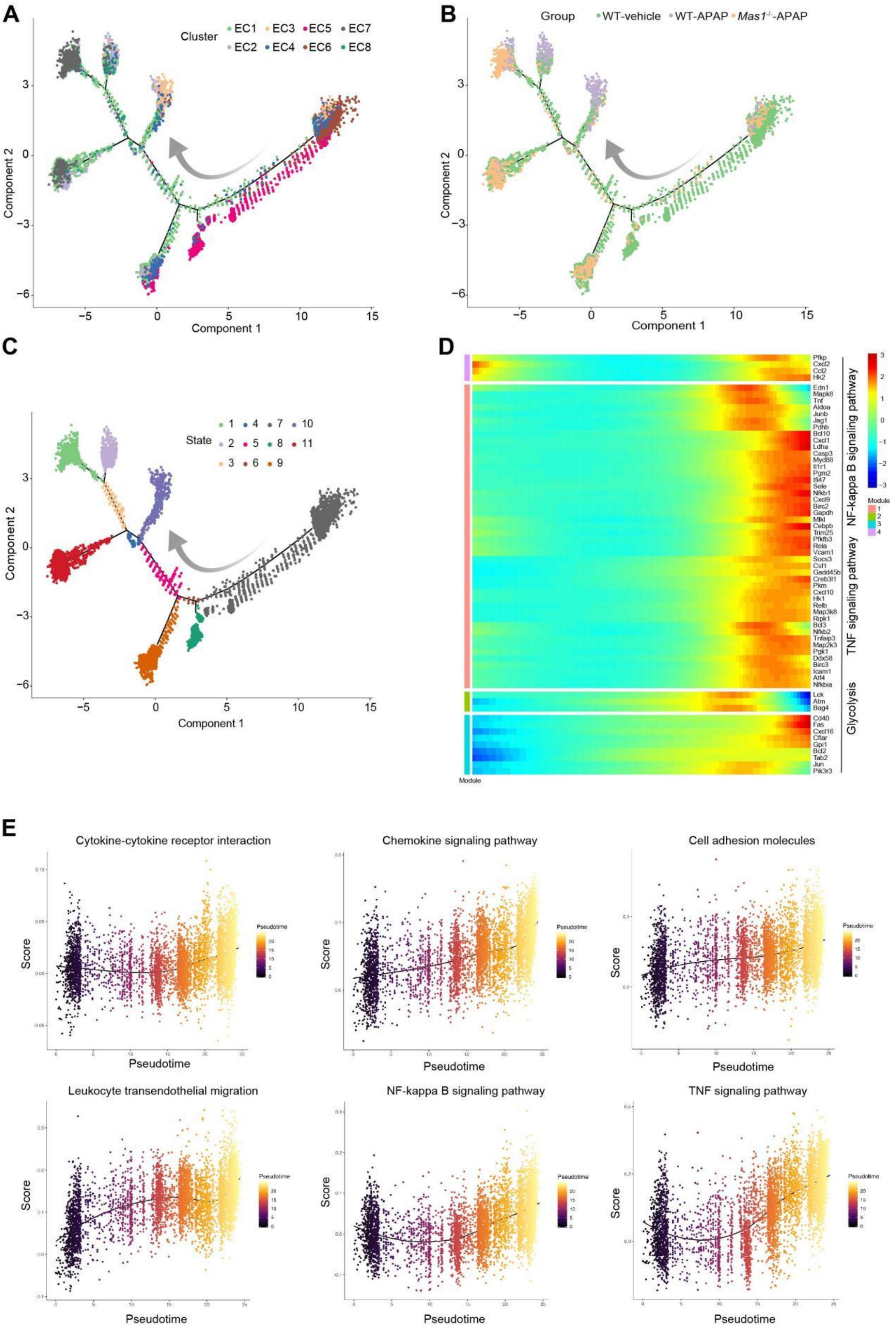

Figure S4. Pseudotime trajectory of EC clusters in AILI. A-C) Monocle analyses showing the development of ECs. D) Heatmap showing expression of representative identified genes potentially associated with Glycolysis, TNF

signaling pathway and NF-kappa B signaling pathway. Color key from blue to red indicates relative expression levels from low to high. E) Smoothed expression curves of representative pathways over pseudotime. EC, endothelial cell; APAP, acetaminophen; TNF, tumor necrosis factor; NF-κB, nuclear factor kappa B.

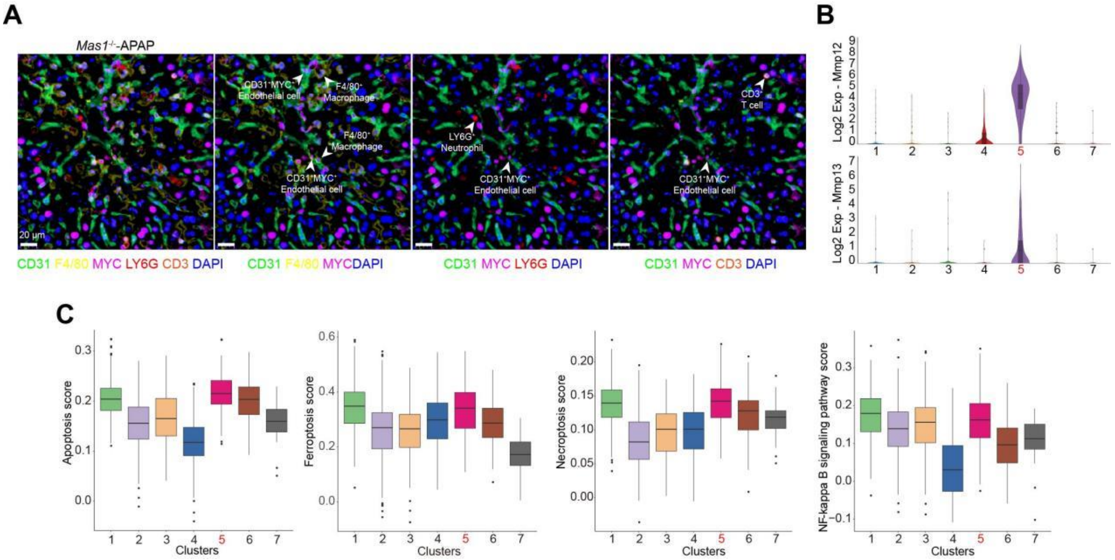

Figure S5. The characteristics of the pro-inflammatory Mψ subgroup. A) mIHC of CD31$^+$MYC$^+$ ECs, Mψ (F4/80$^+$), neutrophil (Ly6G$^+$) and T cells (CD3$^+$). Scale bar: 20 μm. B) Violin plots showing the expression of marker genes for Mψ5. C) Box plots showing the score of indicated pathways for Mψ clusters. APAP, acetaminophen.

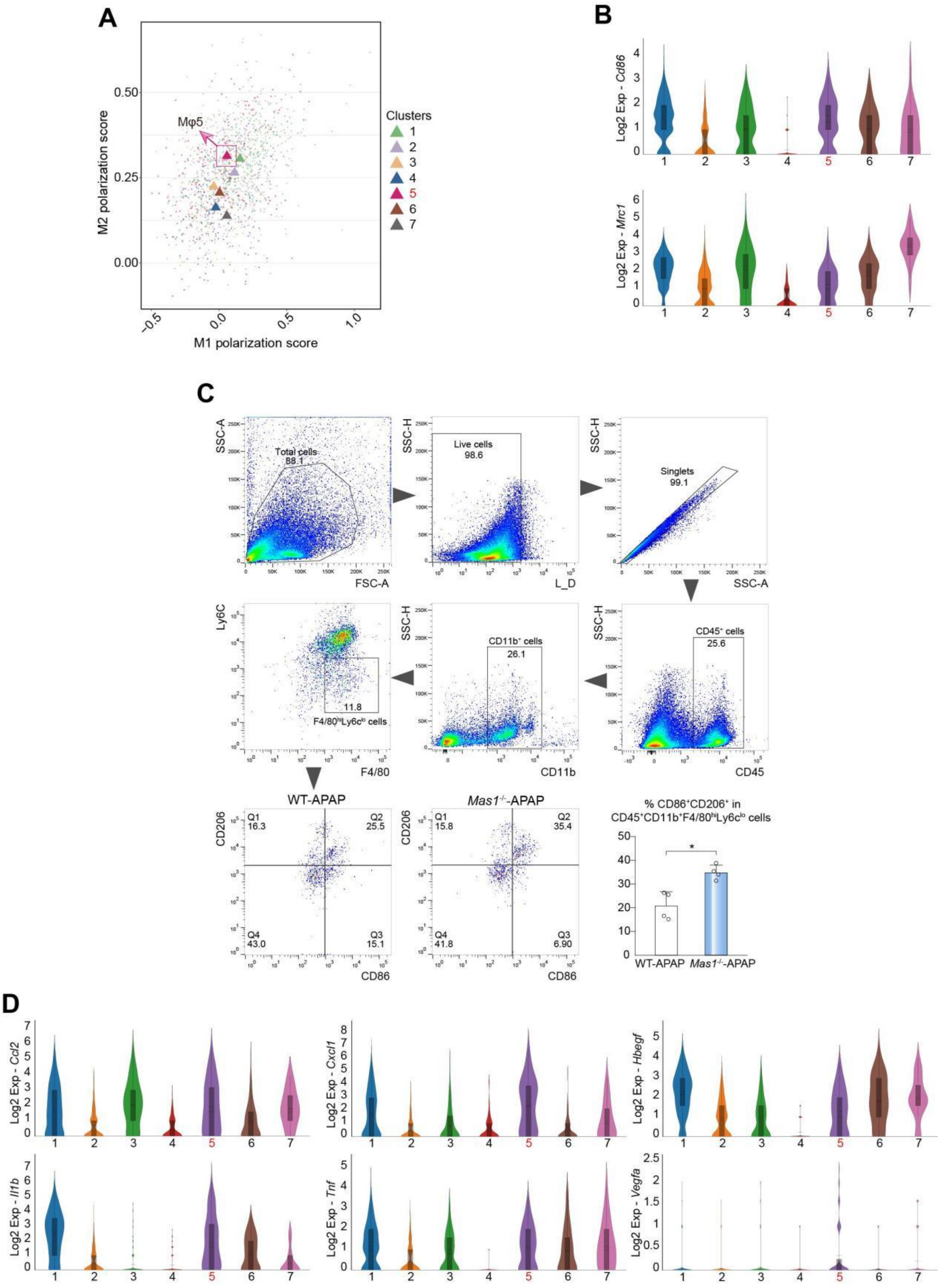

Figure S6. Annotation of the pro-inflammatory Mψ subgroup. A) Scatterplots showing the M1 and M2 scores for each color-coded Mψ subgroup. B) Violin plots showing the marker genes of Mψ polarization for Mψ clusters. C) Representative flow cytometric plots of hepatic CD45$^+$CD11b$^+$F4/80^{high}Ly6C^{low} cells showing CD86$^+$CD206$^+$ fractions between groups (n = 4 mice per group;

two-sided Student’s t-test, P = 9.83x10^{-3}). D) Violin plots showing the key genes of proinflammatory factor and angiogenic factor for Mψ clusters. In all graphs data are presented as mean ± SD, *P < 0.05. Mψ, macrophages; Exp, expression; APAP, acetaminophen.

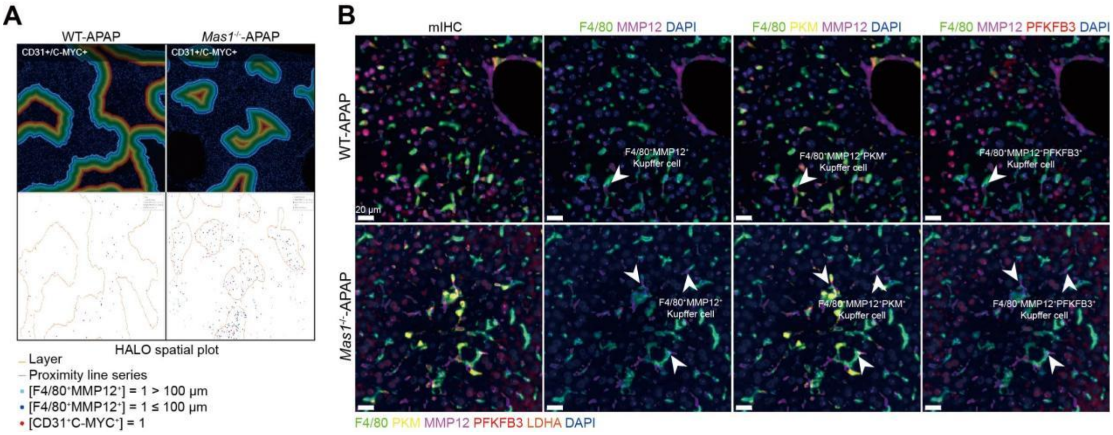

Figure S7. EC-secreting lactate induces Mψ glycolysis. A) Spatial localization of CD31$^+$MYC$^+$ ECs and F4/80$^+$MMP12$^+$ Mψ within injured regions. B) mIHC of F4/80$^+$MMP12$^+$ Mψ and key glycolytic enzymes (PFKFB3 and PKM). Scale bar: 20 μm. APAP, acetaminophen; mIHC, multiplex immunohistochemistry.

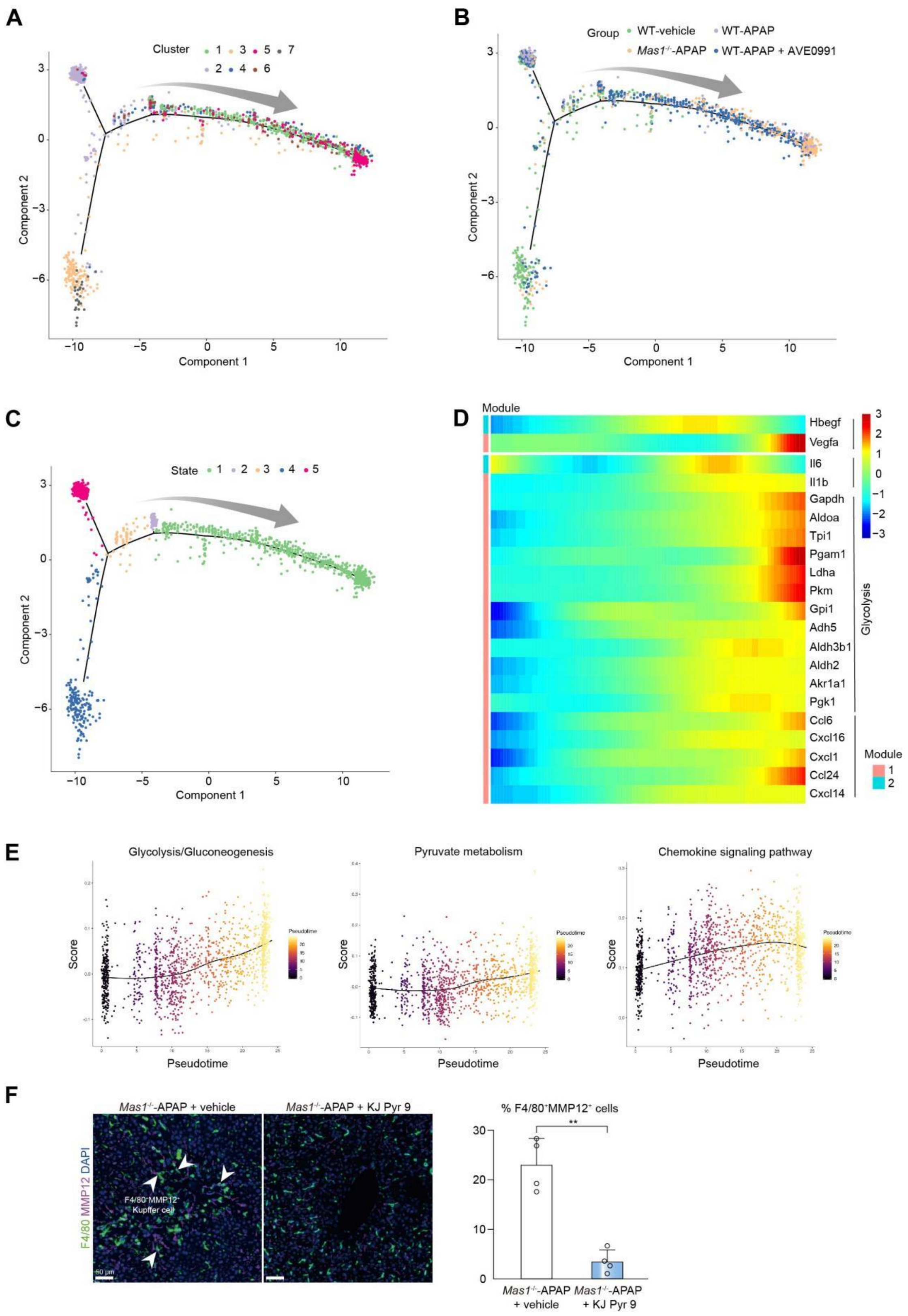

Figure S8. Pseudotime trajectory of Mψ clusters in AILI. A-C) Monocle analyses showing the development of Mψ. D) Heatmap showing expression of representative identified genes potentially associated with glycolysis. Color key from blue to red indicates relative expression levels from low to high. E)

Smoothed expression curves of representative pathways over pseudotime. F) *Mas1*$^{-/-}$ mice were administrated with or without KJ pyr9 2 h after APAP challenge (n = 4 mice per group). mIHC of F4/80^{+}MMP12^{+} Mψ and quantification of F4/80^{+}MMP12^{+} Mψ are shown (two-sided Student's t-test, P = 2.41x10^{-3}). Scale bar: 50 μm. In all graphs data are presented as mean ± SD, **P < 0.01. APAP, acetaminophen; mIHC, multiplex immunohistochemistry.

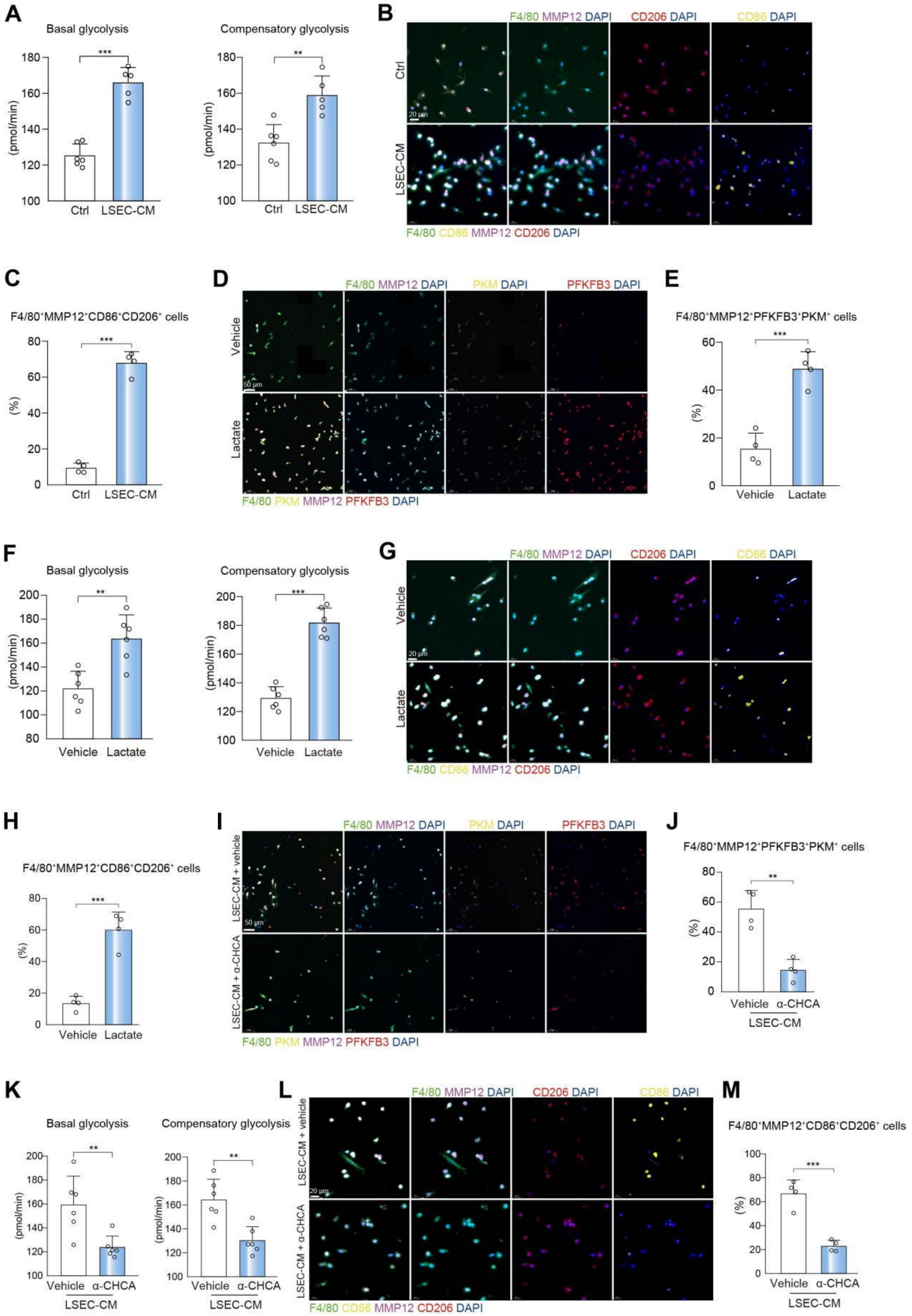

Figure S9. EC-secreting lactate induces Mψ glycolysis and polarization. A) Glycolytic rate assay showing glycolytic function of mouse primary kupffer cells treated with or without LSEC-CM for 24 h. (n = 5 or 6 samples per group; two-sided Student's t-test, P = 7.00x10^{-6} and 2.21x10^{-3} from left to right). B)

Multiplex fluorescence of F4/80$^+$MMP12$^+$ kupffer cells and the markers of Mψ polarization (CD206 and CD86). Scale bar: 20 μm. The mouse primary kupffer cells were treated with or without LSEC-CM for 24 h. C) Quantification of F4/80$^+$MMP12$^+$CD86$^+$CD206$^+$ kupffer cells for multiplex fluorescence as shown in B (n = 4 samples per group; two-sided Student's t-test, P = 3.00x10^{-6}). D) Multiplex fluorescence of F4/80$^+$MMP12$^+$ kupffer cells and key glycolytic enzymes (PKM and PFKFB3). Scale bar: 50 μm. The mouse primary kupffer cells were treated with or without Lactate (10 mM) for 24 h. E) Quantification of F4/80$^+$MMP12$^+$PFKFB3$^+$PKM$^+$ kupffer cells for multiplex fluorescence as shown in D (n = 4 samples per group; two-sided Student's t-test, P = 4.55x10^{-4}). F) Glycolytic rate assay showing glycolytic function of mouse primary kupffer cells treated with or without lactate (10 mM) for 24 h. (n = 6 samples per group; two-sided Student's t-test, P = 2.03x10^{-3} and 2.00x10^{-6} from left to right). G) Multiplex fluorescence of F4/80$^+$MMP12$^+$ kupffer cells and the markers of Mψ polarization (CD206 and CD86). Scale bar: 20 μm. The mouse primary kupffer cells were treated with or without lactate (10 mM) for 24 h. H) Quantification of F4/80$^+$MMP12$^+$CD86$^+$CD206$^+$ kupffer cells for multiplex fluorescence as shown in G (n = 4 samples per group; two-sided Student's t-test, P = 2.42x10^{-4}). I) Multiplex fluorescence of F4/80$^+$MMP12$^+$ kupffer cells and key glycolytic enzymes (PKM and PFKFB3). Scale bar: 50 μm. The mouse primary kupffer cells were treated with LSEC-CM for 24 h, with or without α-CHCA (5 mM) treatment. J) Quantification of F4/80$^+$MMP12$^+$PFKFB3$^+$PKM$^+$ kupffer cells for multiplex fluorescence as shown in I (n = 4 samples per group; two-sided Student's t-test, P = 1.16x10^{-3}). K) Glycolytic rate assay showing glycolytic function of mouse primary kupffer cells treated with LSEC-CM for 24 h, with or without α-CHCA (5 mM) treatment. (n = 6 samples per group; two-sided Student's t-test, P = 6.79x10^{-3} and 2.29x10^{-3} from left to right). L) Multiplex fluorescence of F4/80$^+$MMP12$^+$ kupffer cells and the markers of Mψ polarization (CD206 and CD86). Scale bar: 20 μm. The mouse primary kupffer cells were treated with LSEC-CM for 24 h, with or without α-CHCA (5 mM)

treatment. M) Quantification of $F4/80^+MMP12^+CD86^+CD206^+$ kupffer cells for multiplex fluorescence as shown in L (*n* = 4 samples per group; two-sided Student's t-test, $P = 3.91 \times 10^{-4}$). In all graphs data are presented as mean ± SD, $^{**}P < 0.01$; $^{***}P < 0.001$. LSEC, liver sinusoidal endothelial cell; CM, conditioned medium; α-CHCA, α-cyano-4-hydroxycinnamic acid.

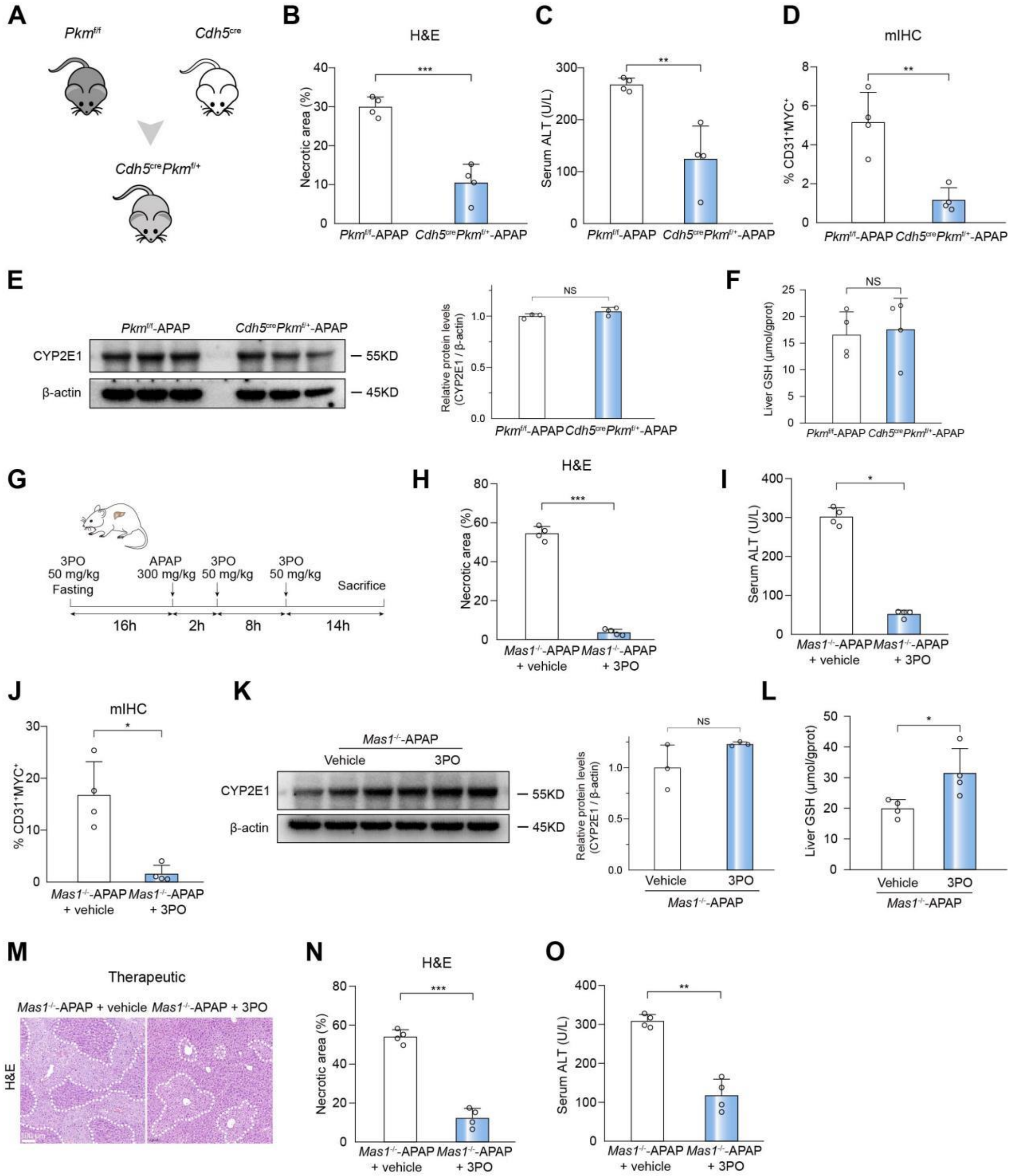

Figure S10. Inhibition of glycolysis alleviates AILI. A) The endothelial cell-specific *Pkm* knockout (*Cdh5*^{cre}*Pkm*$^{f/+}$) mice were generated by crossing *Pkm*$^{f/f}$ with *Cdh5*^{cre} mice. B) Quantification of necrotic area for H&E as shown in Figure 2L (n = 4 mice per group; two-sided Student's t-test, P = 3.56x10^{-4}). C) Serum ALT, a measure of hepatic injury (n = 4 mice per group; two-sided Student's t-test, P = 4.46x10^{-3}). D) Quantification of CD31^{+}MYC^{+} ECs for mIHC as shown in Figure 2L (n = 4 mice per group; two-sided Student's t-test, P = 2.92x10^{-3}). E) Representative liver immunoblots and quantification of CYP2E1 and β-actin (n = 3 mice per group; two-sided Student's t-test, P = 1.64x10^{-1}). F)

Hepatic levels of GSH (n = 4 mice per group, two-sided Student's t-test, P = 7.92x10^{-1}). G) *Mas1*$^{-/-}$ mice were administrated with or without 3PO under APAP challenge as indicated (n = 4 mice per group). H) Quantification of necrotic area for H&E as shown in Figure 2M (n = 4 mice per group; two-sided Student's t-test, P = 2.00x10^{-7}). I) Serum ALT, a measure of hepatic injury (n = 4 mice per group; two-sided Mann-Whitney U test, P = 2.86x10^{-2}). J) Quantification of CD31^{+}MYC^{+} ECs for mIHC as shown in Figure 2M (n = 4 mice per group; two-sided Mann-Whitney U test, P = 2.86x10^{-2}). K) Representative liver immunoblots and quantification of CYP2E1 and β-actin (n = 3 mice per group; two-sided Student's t-test, P = 1.46x10^{-1}). L) Hepatic levels of GSH (n = 4 mice per group, two-sided Student's t-test, P = 3.46x10^{-2}). M) Representative H&E staining. Scale bar: 100 μm. *Mas1*$^{-/-}$ mice were therapeutically administrated with or without 3PO 2 h after APAP challenge (n = 4 mice per group). N) Quantification of necrotic area for H&E as shown in M (n = 4 mice per group; two-sided Student's t-test, P = 9.00x10^{-6}). O) Serum ALT, a measure of hepatic injury (n = 4 mice per group; two-sided Student's t-test, P = 1.09x10^{-3}). In all graphs data are presented as mean ± SD, *P < 0.05; **P < 0.01; ***P < 0.001, NS, not significant. APAP, acetaminophen; ALT, alaninetransaminase; mIHC, multiplex immunohistochemistry; H&E, hematoxylin-eosin.

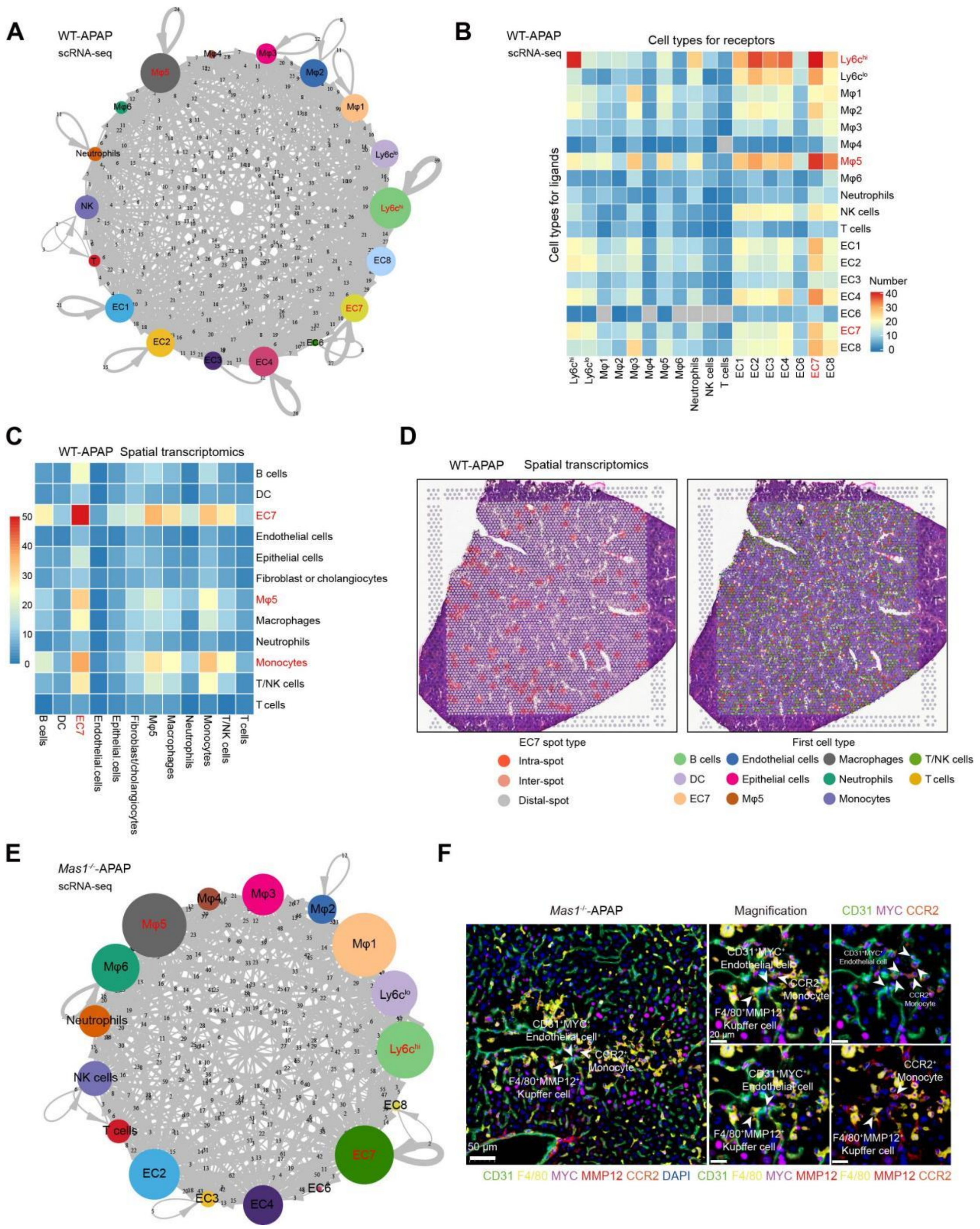

Figure S11. The crosstalk between monocyte and CD31$^+$MYC$^+$CD63$^+$ EC subgroup. A) CellPhoneDB analysis showing the cell–cell interactions for scRNA-seq. B) CellPhoneDB analysis showing the cell–cell interactions for scRNA-seq. C) CellPhoneDB analysis showing the cell–cell interactions for ST. D) Schematic diagram indicating the intra-spots, inter-spots and distal-spots for ST. Intra-spots, EC7-localized spots; Inter-spots, EC7-surrounded spots; Distal-spots, other distant spots. Spatial feature plots showing the first cell type

in EC7 expansion units. E) CellPhoneDB analysis showing the cell–cell interactions for scRNA-seq. F) mIHC of $CD31^{+}MYC^{+}$ ECs, $F4/80^{+}MMP12^{+}$ Mψ and $CCR2^{+}$ monocytes are shown. Scale bar: 50 μm and 20 μm. APAP, acetaminophen; scRNA-seq, single-cell RNA sequencing; Mψ, macrophages; EC, endothelial cell.

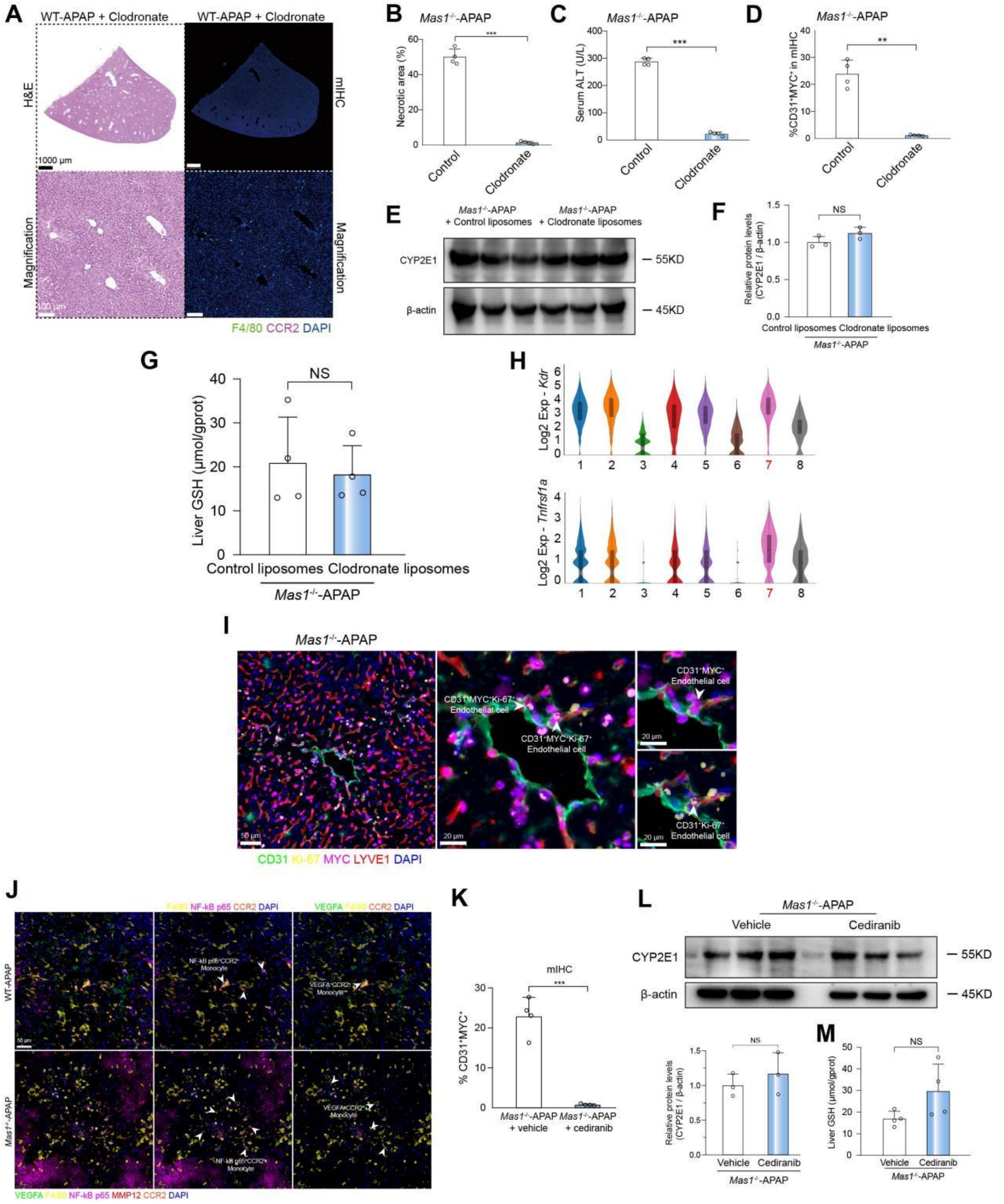

Figure S12. *Mas1* deficiency induces a pro-inflammatory response in monocytes, and pro-inflammatory monocytes induce CD31$^+$MYC$^+$CD63$^+$ EC subgroup proliferation. A) WT mice were pre-administrated with clodronate liposomes for 24 h before APAP challenge (*n* = 4 mice per group). Representative stainings of H&E and mIHC are shown. Scale bar: 1000 μm and 100 μm. B) Quantification of necrotic area for H&E as shown in Figure 3C (*n* = 4 mice per group; two-sided Student's t-test, *P* = 6.43x10^{-7}). C) Serum ALT, a measure of hepatic injury (*n* = 4 mice per group; two-sided Student's t-test, *P*

= 1.00×10^{-6}). D) Quantification of $CD31^+MYC^+$ ECs for mIHC as shown in Figure 3C (n = 4 mice per group; two-sided Student's t-test, $P = 2.88 \times 10^{-3}$). E) Representative liver immunoblots of CYP2E1 and β-actin. F) Quantification of CYP2E1 and β-actin (two-sided Student's t-test, $P = 1.23 \times 10^{-1}$). G) Hepatic levels of GSH (n = 4 mice per group, two-sided Student's t-test, $P = 6.88 \times 10^{-1}$). H) Violin plots showing the indicated genes of EC clusters. I) mIHC of the proliferative $CD31^+MYC^+Ki\text{-}67^+$ ECs. Scale bar: 50 μm and 20 μm. J) WT mice and *Mas1*$^{-/-}$ mice were administrated with APAP for 24 h (n = 4 mice per group). mIHC of NF-κB $p65^+VEGFA^+CCR2^+$ monocytes. Scale bar: 50 μm. K) Quantification of $CD31^+MYC^+$ ECs for mIHC as shown in Figure 3F (n = 4 mice per group; two-sided Student's t-test, $P = 9.30 \times 10^{-5}$). L) Representative liver immunoblots and quantification of CYP2E1 and β-actin (n = 3 mice per group; two-sided Student's t-test, $P = 4.48 \times 10^{-1}$). M) Hepatic levels of GSH (n = 4 mice per group, two-sided Student's t-test, $P = 1.34 \times 10^{-1}$). In all graphs data are presented as mean ± SD, $^{**}P < 0.01$; $^{***}P < 0.001$; NS, not significant. APAP, acetaminophen; H&E, hematoxylin-eosin; mIHC, multiplex immunohistochemistry; ALT, alaninetransaminase; Exp, expression.

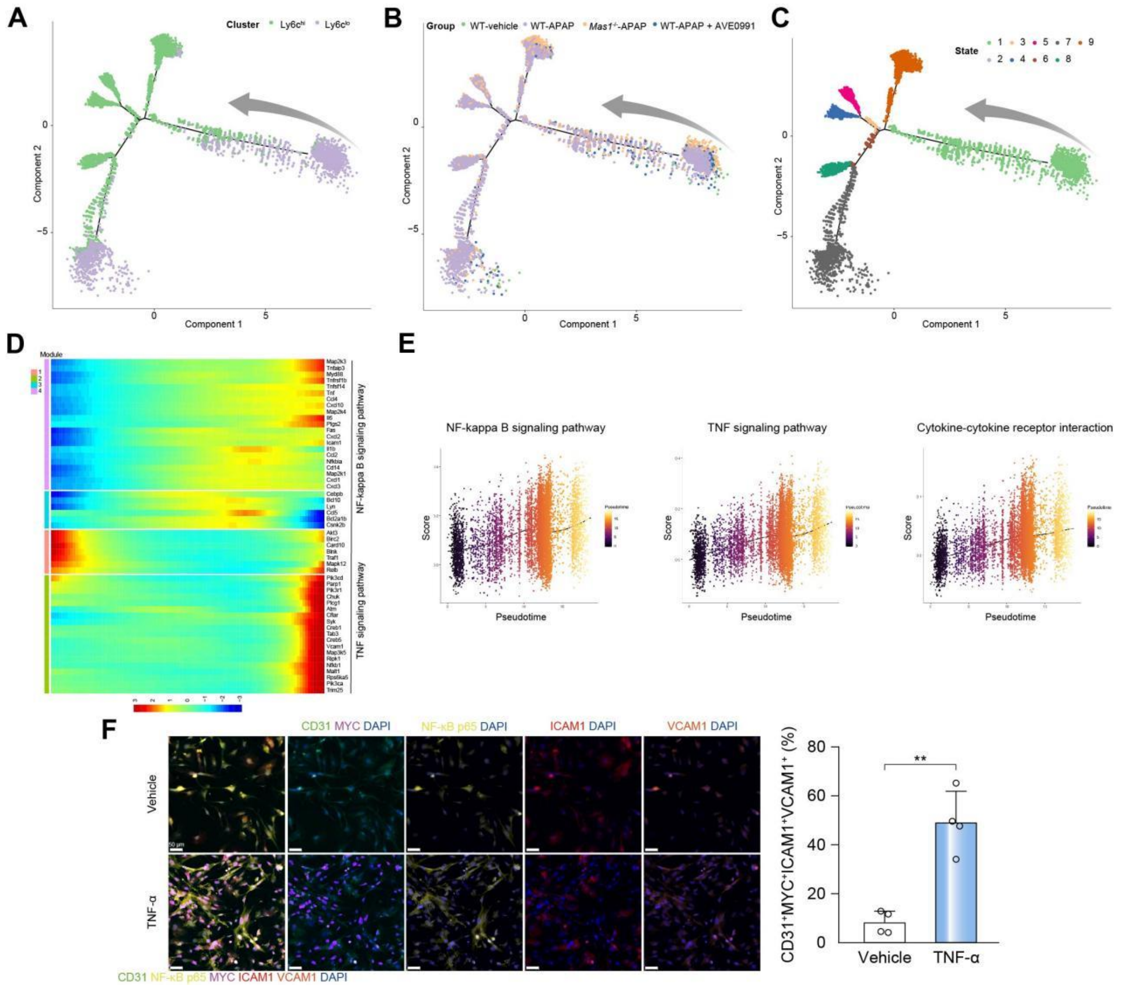

Figure S13. Pseudotime trajectory of monocytes clusters in AILI. A-C) Monocle analyses showing the development of monocytes. D) Heatmap showing expression of representative identified genes potentially associated with TNF signaling pathway and NF-kappa B signaling pathway. Color key from blue to red indicates relative expression levels from low to high. E) Smoothed expression curves of representative pathways over pseudotime. F) Multiplex fluorescence of $CD31^{+}MYC^{+}ICAM1^{+}VCAM1^{+}$ LSECs. Scale bar: 50 μm. The mouse primary LSECs were treated with or without TNF-α (20 ng ml^{-1}) for 24 h. Quantification of $CD31^{+}MYC^{+}ICAM1^{+}VCAM1^{+}$ LSECs for multiplex fluorescence are shown (*n* = 4 samples per group; two-sided Student's t-test, $P = 9.39 \times 10^{-4}$). In all graphs data are presented as mean ± SD, $^{**}P < 0.01$. TNF-α, tumor necrosis factor-α; APAP, acetaminophen; NF-kappa B, NF-κB, nuclear factor kappa B.

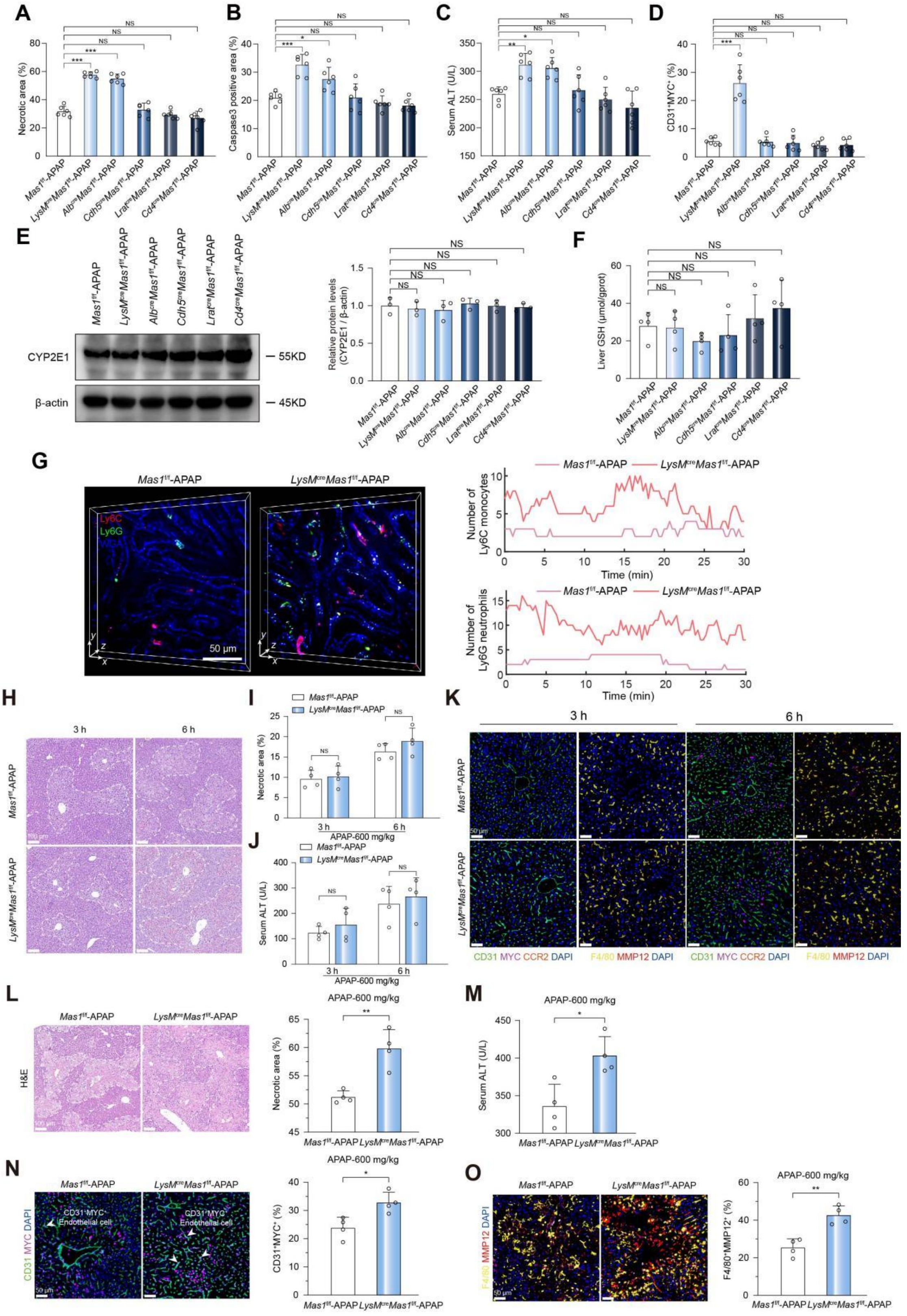

Figure S14. Myeloid *Mas1* deficiency exacerbates AILI. A) Quantification of necrotic area for H&E as shown in Figure 4A (n = 6 mice per group; One-way ANOVA with Tukey's test, P = 6.61x10^{-12}, 9.34x10^{-11}, 9.93x10^{-1}, 8.93x10^{-1} and 2.70x10^{-1} from left to right). B) Quantification of caspase3 positive area for

mIHC as shown in Figure 4A (n = 6 mice per group; One-way ANOVA with Tukey's test, $P = 3.10 \times 10^{-5}$, 2.87×10^{-2}, 1.00, 9.38×10^{-1} and 7.26×10^{-1} from left to right). C) Serum ALT, a measure of hepatic injury (n = 6 mice per group; One-way ANOVA with Tukey's test, $P = 4.75 \times 10^{-3}$, 1.49×10^{-2}, 9.95×10^{-1}, 9.74×10^{-1} and 4.32×10^{-1} from left to right). D) Quantification of $CD31^+MYC^+$ ECs for mIHC as shown in Figure 4A (n = 6 mice per group; One-way ANOVA with Tukey's test, $P = 6.33 \times 10^{-11}$, 1.00, 9.99×10^{-1}, 9.69×10^{-1} and 9.79×10^{-1} from left to right). E) Representative liver immunoblots and quantification of CYP2E1 and β-actin (n = 3 mice per group; two-sided Student's t-test, P = 6.49×10^{-1}, 5.82×10^{-1}, 7.20×10^{-1}, 9.81×10^{-1} and 7.79×10^{-1} from left to right). F) Hepatic levels of GSH (n = 4 mice per group; two-sided Student's t-test, P = 8.80×10^{-1}, 1.07×10^{-1}, 4.86×10^{-1}, 5.96×10^{-1} and 2.98×10^{-1} from left to right). G) Timelapse data of neutrophils (Ly6G) and monocytes (Ly6C) in the vessels (WGA) of living mouse livers were captured by DAOSLIMIT. Representative intravital images and the temporal traces of their number are shown. *Mas1*$^{f/f}$ and *LysM*^{cre}*Mas1*$^{f/f}$ mice were challenged with APAP for 24 h. Scale bar: 50 μm. In H-K, *Mas1*$^{f/f}$ and *LysM*^{cre}*Mas1*$^{f/f}$ mice were administrated with APAP (600 mg kg^{-1}) for 3 h or 6 h (n = 4 mice per group). (H) Representative stainings of H&E are shown. Scale bar: 100 μm. (I) Quantification of H&E are shown (n = 4 mice per group; two-sided Student's t-test, $P = 7.27 \times 10^{-1}$ and 2.28×10^{-1} from left to right). (J) Serum ALT (n = 4 mice per group; two-sided Student's t-test, P = 4.15×10^{-1} and 5.91×10^{-1} from left to right). (K) Representative stainings of $CD31^+MYC^+$ ECs and $F4/80^+MMP12^+$ Mψ for mIHC (n = 4 mice per group). Scale bar: 50 μm. In L-O, *Mas1*$^{f/f}$ and *LysM*^{cre}*Mas1*$^{f/f}$ mice were administrated with APAP (600 mg kg^{-1}) for 24 h (n = 4 mice per group). (L) Representative stainings and quantification of H&E are shown (n = 4 mice per group; two-sided Student's t-test, $P = 2.80 \times 10^{-3}$). Scale bar: 100 μm. (M) Serum ALT (n = 4 mice per group; two-sided Student's t-test, $P = 1.31 \times 10^{-2}$). (N) Representative stainings and quantification of $CD31^+MYC^+$ ECs for mIHC (n = 4 mice per group; two-sided Student's t-test, $P = 1.49 \times 10^{-2}$). Scale bar: 50 μm.

(O) Representative stainings and quantification of F4/80^{+}MMP12^{+} Mψ for mIHC (*n* = 4 mice per group; two-sided Student's t-test, *P* = 2.29x10^{-3}). Scale bar: 50 μm. In all graphs data are presented as mean ± SD, *P < 0.05; **P < 0.01; ***P < 0.001; NS, non-significant. APAP, acetaminophen; mIHC, multiplex immunohistochemistry; ALT, alaninetransaminase.

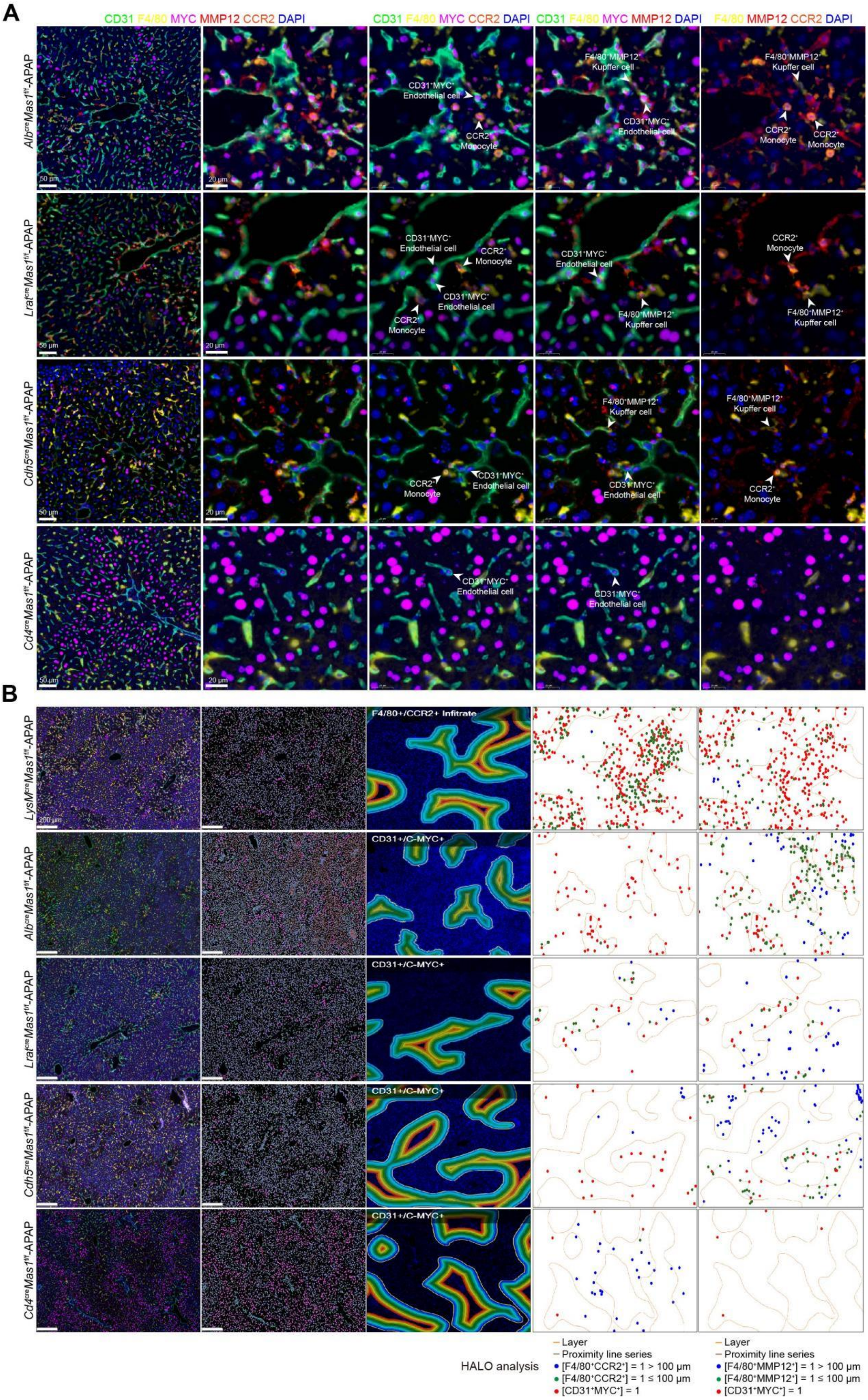
A
CD31 F4/80 MYC MMP12 CCR2 DAPI
CD31 F4/80 MYC CCR2 DAPI
CD31 F4/80 MYC MMP12 DAPI
F4/80 MMP12 CCR2 DAPI
Alb^cre Mas1^f/f-APAP
Lrat^cre Mas1^f/f-APAP
Cdh5^cre Mas1^f/f-APAP
Cd4^cre Mas1^f/f-APAP
CD31⁺MYC⁺ Endothelial cell
CCR2⁺ Monocyte
F4/80⁺MMP12⁺ Kupffer cell
50 μm
20 μm
B
LysM^cre Mas1^f/f-APAP
200 μm
F4/80+/CCR2+ Infitrate
CD31+/C-MYC+
HALO analysis
Layer
Proximity line series
[F4/80⁺CCR2⁺] = 1 > 100 μm
[F4/80⁺CCR2⁺] = 1 ≤ 100 μm
[CD31⁺MYC⁺] = 1
[F4/80⁺MMP12⁺] = 1 > 100 μm
[F4/80⁺MMP12⁺] = 1 ≤ 100 μm

Figure S15. Myeloid *Mas1* deficiency results in pro-inflammatory microenvironment in AILI. A-B) mIHC of $CD31^+MYC^+$ ECs, $F4/80^+MMP12^+$ Mψ and $CCR2^+$ monocytes are shown. Scale bar: 50 μm and 20 μm. Spatial localization of $CD31^+MYC^+$ ECs, $CCR2^+$ monocytes and $F4/80^+MMP12^+$ Mψ within injured region are shown. Scale bar: 200 μm. APAP, acetaminophen.

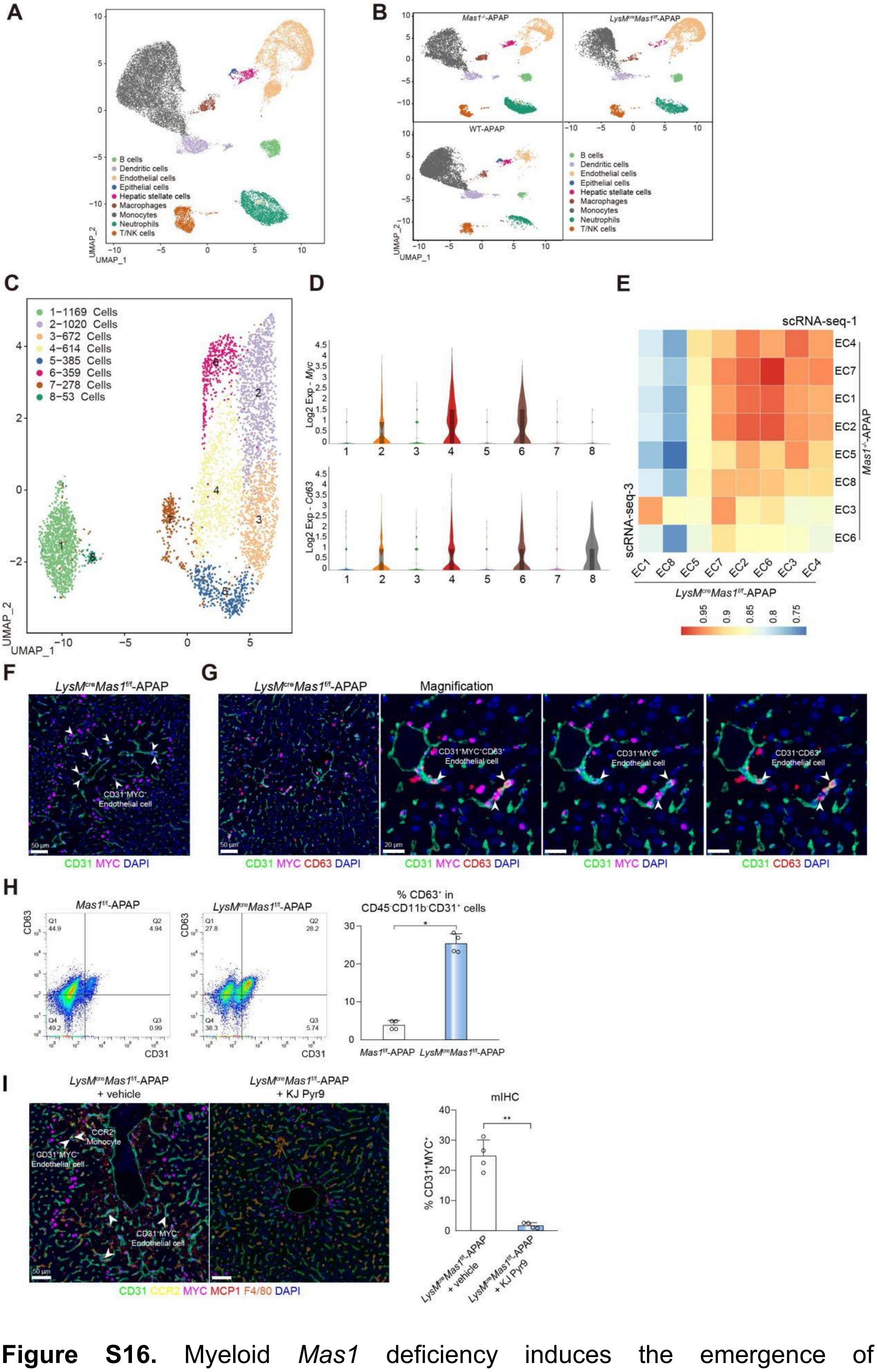

Figure S16. Myeloid *Mas1* deficiency induces the emergence of CD31$^+$MYC$^+$CD63$^+$ ECs in AILI. A) The UMAP plot of intrahepatic main cell

types. B) The UMAP plot showing the differences of intrahepatic main cell types between different groups. C) The UMAP plot of 4,550 single cells from ECs. D) Violin plots showing the marker genes of EC clusters. E) Correlation analysis of the scRNA-seq. F) mIHC of the $CD31^+MYC^+$ ECs. Scale bar: 50 μm. G) *LysM*^{cre}*Mas1*$^{f/f}$ mice were challenged with APAP for 24 h (n = 4 mice per group). mIHC of $CD31^+MYC^+CD63^+$ ECs are shown. Scale bar: 50 μm and 20 μm. H) *Mas1*$^{f/f}$ and *LysM*^{cre}*Mas1*$^{f/f}$ mice were challenged with APAP for 24 h (n = 4 mice per group). Representative flow cytometric plots of hepatic $CD45^-CD11b^-CD31^+$ cells showed $CD63^+$ fractions between groups. Quantification of $CD63^+$ fractions are shown (n = 4 mice per group; two-sided Mann-Whitney U test, P = 2.86x10^{-2}). I) *LysM*^{cre}*Mas1*$^{f/f}$ mice were administrated with or without KJ pyr9 2 h after APAP challenge (n = 4 mice per group). mIHC of $CD31^+MYC^+$ ECs and $CCR2^+$ monocytes are shown. Scale bar: 50 μm. Quantification of the $CD31^+MYC^+$ ECs for mIHC are shown (n = 4 mice per group; two-sided Student's t-test, P = 2.48x10^{-3}). In all graphs data are presented as mean ± SD, *P < 0.05; **P < 0.01. UMAP, uniform manifold approximation and projection; Exp, expression; scRNA-seq, single-cell RNA sequencing; EC, endothelial cell; APAP, acetaminophen.

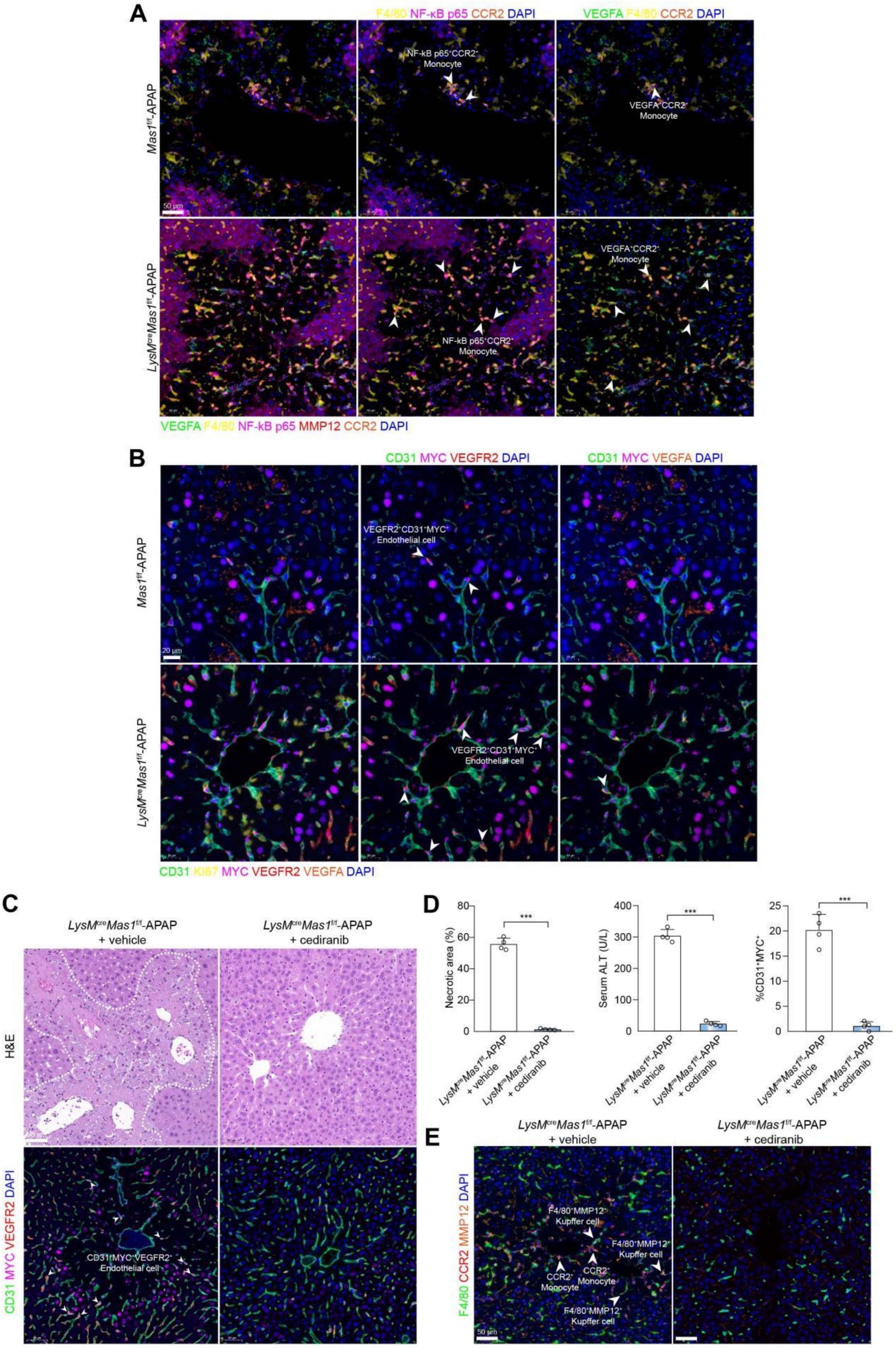

Figure S17. Pro-inflammatory monocytes induce the CD31$^+$MYC$^+$CD63$^+$ EC Subgroup proliferation. In A-B, *Mas1*$^{f/f}$ and *LysM*^{cre}*Mas1*$^{f/f}$ mice were

administrated with APAP for 24 h (n = 4 mice per group). A) mIHC of NF-κB p65$^+$VEGFA$^+$CCR2$^+$ monocytes. Scale bar: 50 μm. B) mIHC of VEGFR2$^+$CD31$^+$MYC$^+$ ECs. Scale bar: 20 μm. In C-E, *LysM*^{cre}*Mas1*$^{f/f}$ mice were pre-administrated with or without cediranib for 2 h before APAP challenge (n = 4 mice per group). C) Representative stainings of H&E and mIHC are shown. Scale bar: 50 μm. D) Quantification of necrotic area for H&E as shown in C (n = 4 mice per group; two-sided Student's t-test, $P = 7.10 \times 10^{-5}$). Serum ALT, a measure of hepatic injury (n = 4 mice per group; two-sided Student's t-test, $P = 2.16 \times 10^{-7}$). Quantification of CD31$^+$MYC$^+$ ECs for mIHC as shown in C (n = 4 mice per group; two-sided Student's t-test, $P = 2.20 \times 10^{-5}$). E) mIHC of F4/80$^+$MMP12$^+$ Mψ and CCR2$^+$ monocytes. Scale bar: 50 μm. In all graphs data are presented as mean ± SD, ***$P < 0.001$. APAP, acetaminophen; NF-κB, nuclear factor kappa B; H&E, hematoxylin-eosin; ALT, alaninetransaminase; mIHC, multiplex immunohistochemistry.

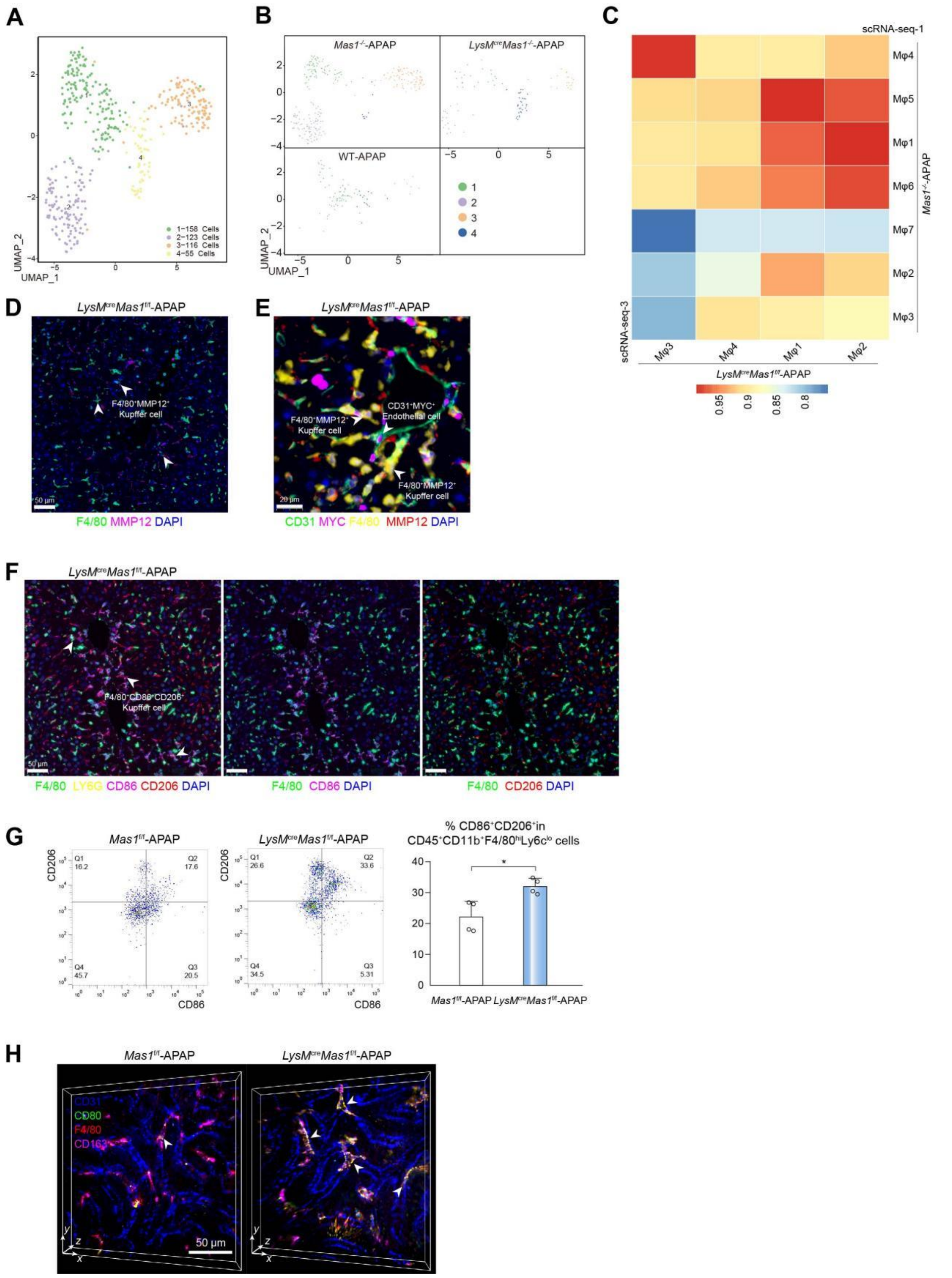

Figure S18. EC-secreting lactate induces Mψ polarization. A) The UMAP plot showing the subpopulations of Mψ. B) The UMAP plot showing the differences of Mψ subpopulations between different groups. C) Correlation analysis of the scRNA-seq. In D-F, *LysM*^{cre}*Mas1*$^{f/f}$ mice were administrated with APAP for 24 h (n = 4 mice per group). D) mIHC of the F4/80$^+$MMP12$^+$ Mψ is shown. Scale bar:

50 μm. E) mIHC of the CD31⁺MYC⁺ ECs and F4/80⁺MMP12⁺ Mψ. Scale bar: 20 μm. F) mIHC of the Mψ polarization (F4/80⁺CD86⁺CD206⁺ Mψ). Scale bar: 50 μm. G) Representative flow cytometric plots of hepatic $CD45^+CD11b^+F4/80^{high}Ly6C^{low}$ cells showed the $CD86^+CD206^+$ fractions between groups (n = 4 mice per group; two-sided Student's t-test, P = 2.08×10^{-2}). H) Representative intravital images of Mψ polarization by DAOSLIMIT. The white arrows represent the $CD80^+CD163^+$ Mψ. Scale bar: 50 μm. In all graphs data are presented as mean ± SD, *P < 0.05. UMAP, uniform manifold approximation and projection; APAP, acetaminophen; scRNA-seq, single-cell RNA sequencing; Mψ, macrophages.

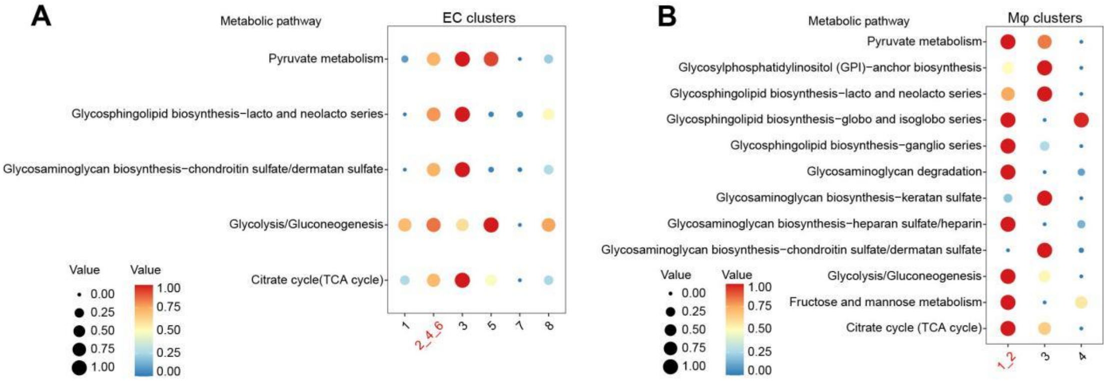

Figure S19. EC-secreting lactate induces Mψ glycolysis. A) Pathway enrichment analysis of highly expressed genes for EC clusters. KEGG gene sets were used to perform the pathway enrichment analysis (Methods). B) Pathway enrichment analysis of highly expressed genes for Mψ clusters. KEGG gene sets were used to perform the pathway enrichment analysis (Methods). In all graphs data are presented as mean ± SD. EC, endothelial cell; Mψ, macrophages.

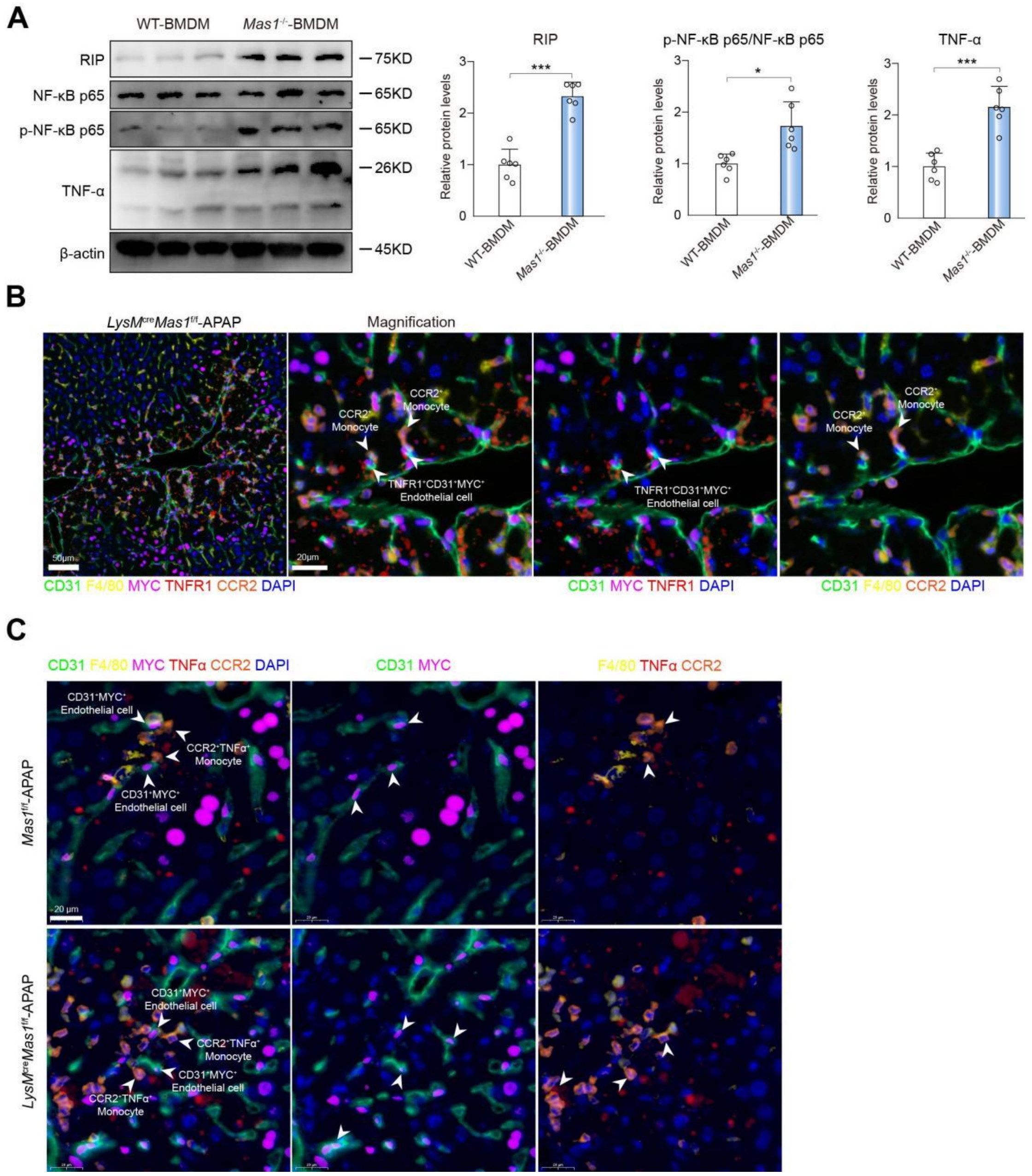

Figure S20. Myeloid *Mas1* deficiency induces the emergence of CD31$^+$MYC$^+$CD63$^+$ ECs in AILI via NF-κB/TNF-α pathway. A) Representative immunoblots with quantification (n = 6 samples per group; two-sided Student's t-test, P = 1.20x10^{-5}, 1.03x10^{-2} and 1.38x10^{-4} from left to right). WT and *Mas1*$^{-/-}$ BMDMs were stimulated with LPS (100 ng ml^{-1}) for 12 h to drive proinflammatory activation. B) mIHC of CD31$^+$MYC$^+$TNFR1$^+$ ECs and CCR2$^+$ monocytes are shown. Scale bar: 50 μm and 20 μm. *LysM*^{cre}*Mas1*$^{f/f}$ mice were challenged with APAP for 24 h (n = 4 mice per group). C) mIHC of CD31$^+$MYC$^+$ ECs and CCR2$^+$TNF-α$^+$ monocytes are shown. Scale bar: 20 μm. *Mas1*$^{f/f}$ and

LysM^{cre}*Mas1*$^{f/f}$ mice were challenged with APAP for 24 h (*n* = 4 mice per group). BMDM, bone marrow derived macrophages; TNF-α, tumor necrosis factor-α; NF-κB, nuclear factor kappa B; APAP, acetaminophen.

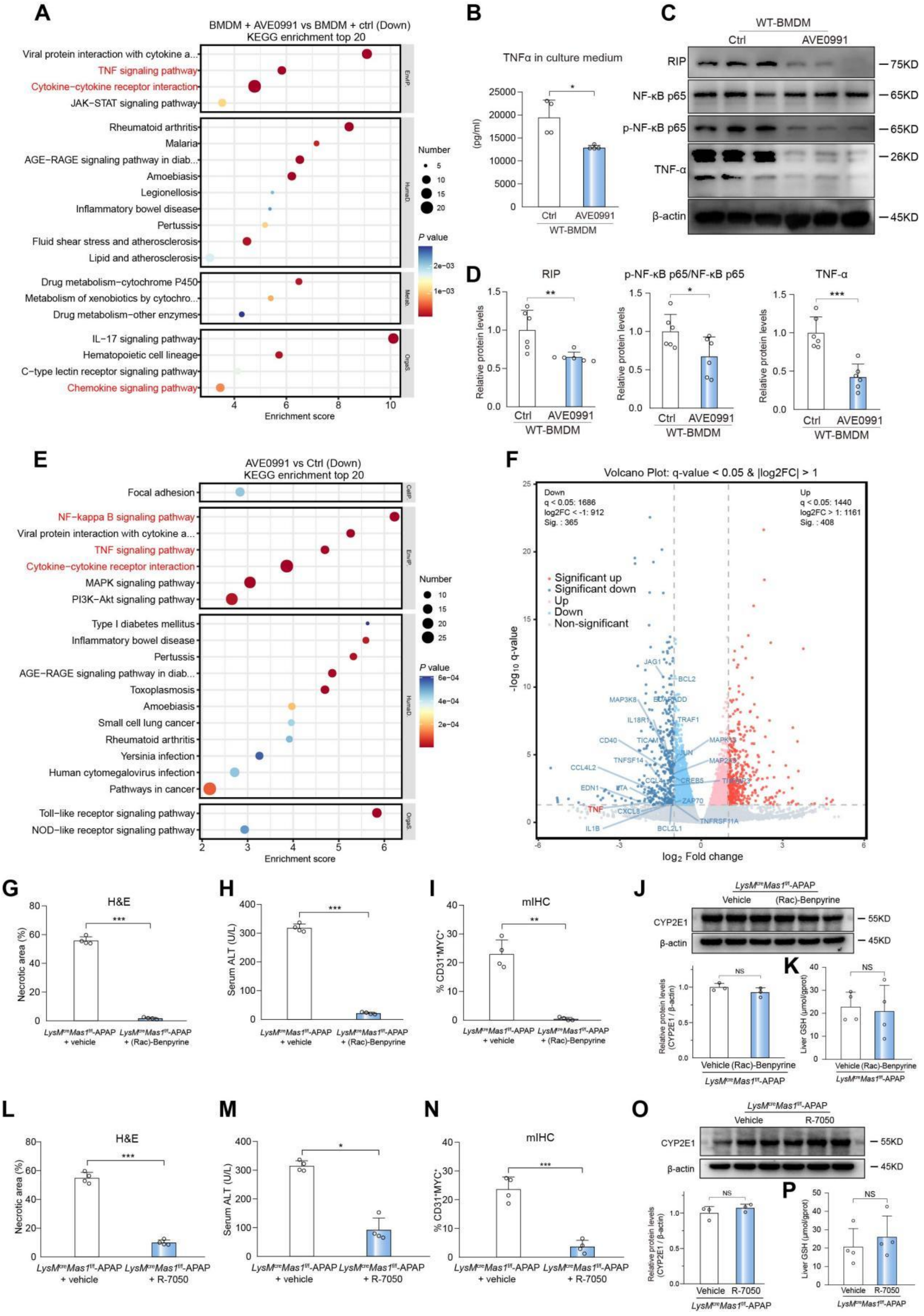

Figure S21. Activation of Mas could significantly inhibit NF-κB/TNF-α pathway. In A-D, BMDMs from WT mice were pre-administrated with or without AVE0991 (5 uM) for 2 h before stimulated with LPS (100 ng ml^{-1}) for 12 h. A) BMDMs were subjected to bulk RNA-seq (*n* = 3 samples per group). KEGG

pathway enrichment analysis show that activation of Mas could significantly down-regulate TNF signaling pathway. B) The TNF-α of the culture medium were tested (n = 4 samples per group; two-sided Student's t-test, P = 3.91x10^{-2}). C-D) Representative immunoblots with quantification (n = 6 samples per group; two-sided Student's t-test and two-sided Mann-Whitney U test, P = 4.33x10^{-3}, 4.08x10^{-2} and 3.68x10^{-4} from left to right). In E-F, monocytes (CD14$^+$ cells) isolated form human peripheral blood were stimulated with LPS (100 ng ml^{-1}) for pro-inflammatory activation with or without AVE0991 (10 uM) administration and then subjected to bulk RNA-seq (n = 3 samples per group). E) KEGG pathway enrichment analysis show that activation of Mas could significantly down-regulate NF-κB and TNF signaling pathway. F) Volcano plot showing the differentially expressed genes for TNF signaling pathway and NF-kappa B signaling pathway. G) Quantification of necrotic area for H&E as shown in Figure 5G (n = 4 mice per group; two-sided Student's t-test, P = 1.68x10^{-8}). H) Serum ALT, a measure of hepatic injury (n = 4 mice per group; two-sided Student's t-test, P = 1.24x10^{-8}). I) Quantification of CD31$^+$MYC$^+$ ECs for mIHC as shown in Figure 5G (n = 4 mice per group; two-sided Student's t-test, P = 2.58x10^{-3}). J) Representative liver immunoblots and quantification of CYP2E1 and β-actin (n = 3 mice per group; two-sided Student's t-test, P = 2.00x10^{-1}). K) Hepatic levels of GSH (n = 4 mice per group, two-sided Student's t-test, P = 7.79x10^{-1}). L) Quantification of necrotic area for H&E as shown in Figure 5H (n = 4 mice per group; two-sided Student's t-test, P = 6.84x10^{-7}). M) Serum ALT, a measure of hepatic injury (n = 4 mice per group; two-sided Mann-Whitney U test, P = 2.86x10^{-2}). N) Quantification of CD31$^+$MYC$^+$ ECs for mIHC as shown in Figure 5H (n = 4 mice per group; two-sided Student's t-test, P = 1.64x10^{-4}). O) Representative liver immunoblots and quantification of CYP2E1 and β-actin (n = 3 mice per group; two-sided Student's t-test, P = 3.01x10^{-1}). P) Hepatic levels of GSH (n = 4 mice per group, two-sided Student's t-test, P = 5.05x10^{-1}). In all graphs data are presented as mean ± SD, *P < 0.05; **P < 0.01; ***P < 0.001, NS, not significant. BMDM,

bone marrow derived macrophages; KEGG, kyoto encyclopedia of genes and genomes; TNF-α, tumor necrosis factor-α; NF-κB, nuclear factor kappa B; FC, fold change; ALT, alaninetransaminase; mIHC, multiplex immunohistochemistry.

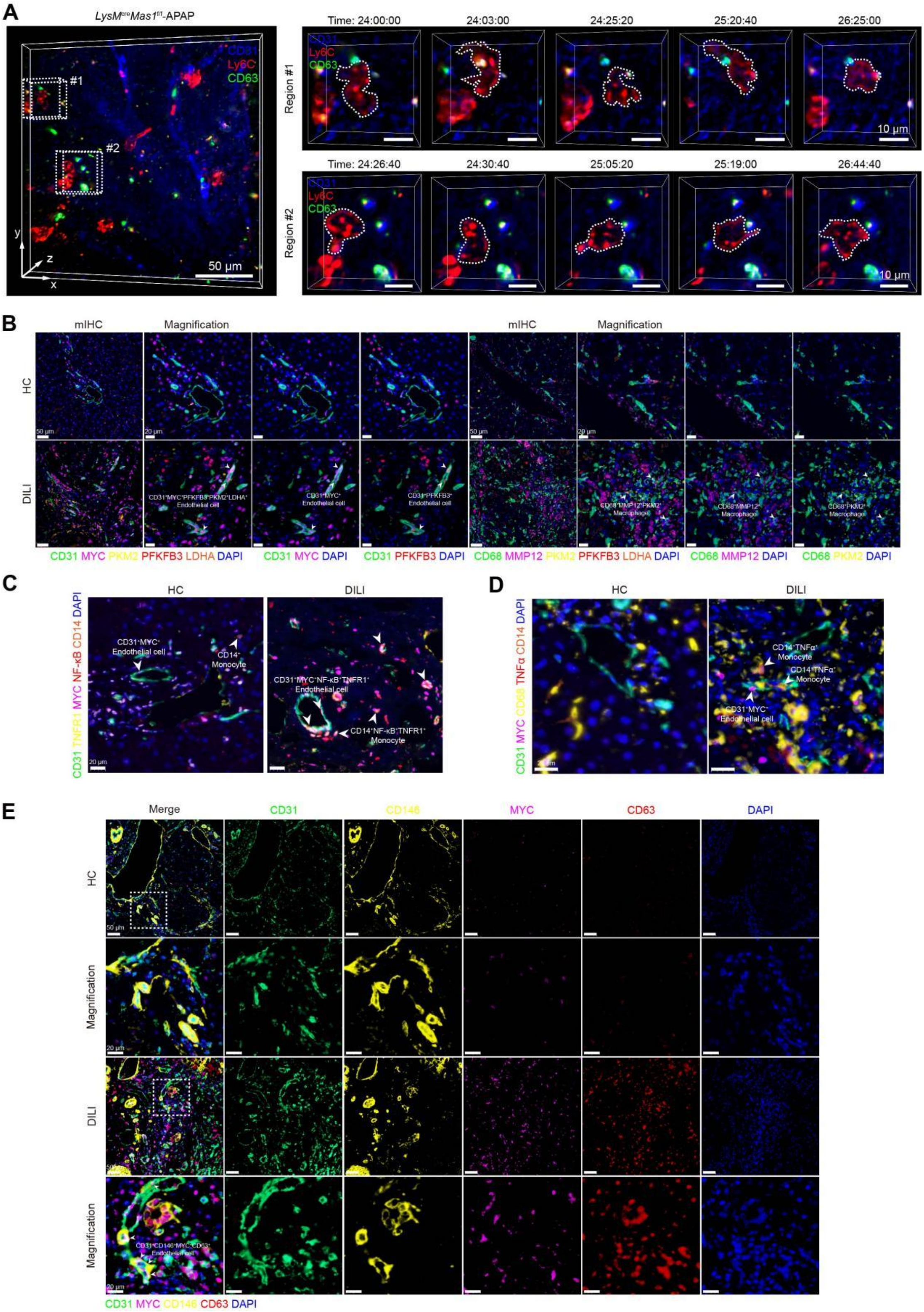

Figure S22. Pro-inflammatory microenvironment were observed by hour-long intravital 3D imaging in mice and enriched in advanced human DILI. A) Timelapse data captured by DAOSLIMIT showing the interactions between monocytes and $CD63^{+}$ ECs in *LysM*^{cre}*Mas1*$^{f/f}$-APAP mouse. Scale bar: 50 μm.

B) mIHC of CD31$^+$MYC$^+$ ECs, CD68$^+$MMP12$^+$ Mψ and key glycolytic enzymes are shown. Scale bar: 50 μm and 20 μm. C) mIHC of CD31$^+$MYC$^+$NF-κB$^+$TNFR1$^+$ ECs and CD14$^+$NF-κB$^+$TNFR1$^+$ monocytes are shown. Scale bar: 20 μm. D) mIHC of CD31$^+$MYC$^+$ ECs and CD14$^+$TNFα$^+$ monocytes are shown. Scale bar: 20 μm. E) mIHC of CD31$^+$MYC$^+$CD146$^+$CD63$^+$ ECs are shown. Scale bar: 50 μm and 20 μm. APAP, acetaminophen; mIHC, multiplex immunohistochemistry; HC, healthy control; DILI, drug-induced liver injury; NF-κB, nuclear factor kappa B; TNF-α, tumor necrosis factor-α.

A

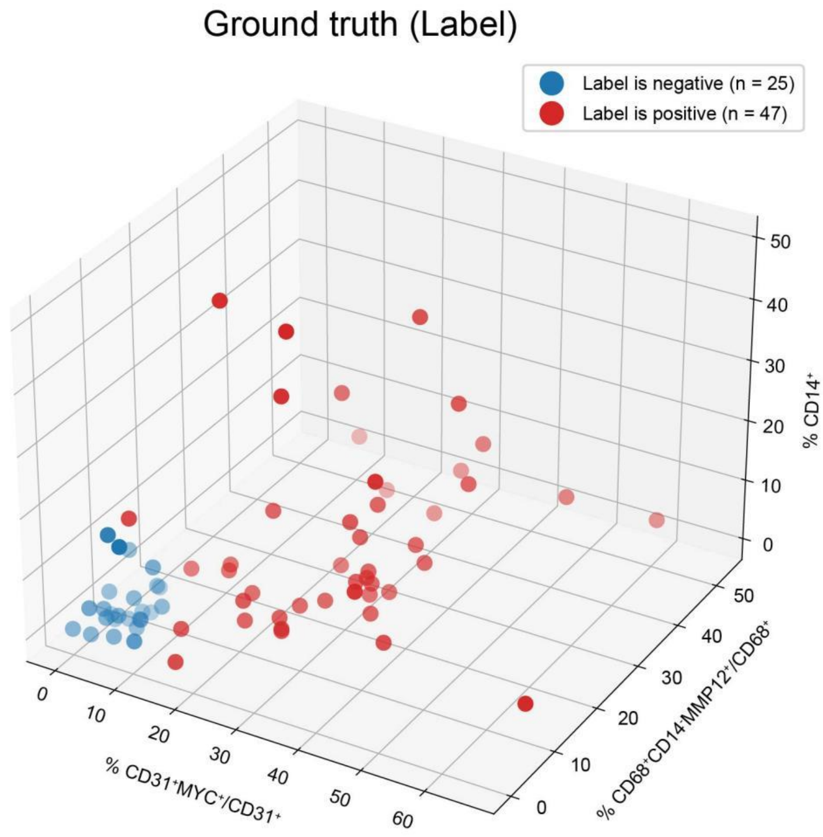
Ground truth (Label)
Label is negative (n = 25)
Label is positive (n = 47)
% CD31⁺MYC⁺/CD31⁺
% CD68⁺CD14⁻MMP12⁺/CD68⁺
% CD14⁺

B

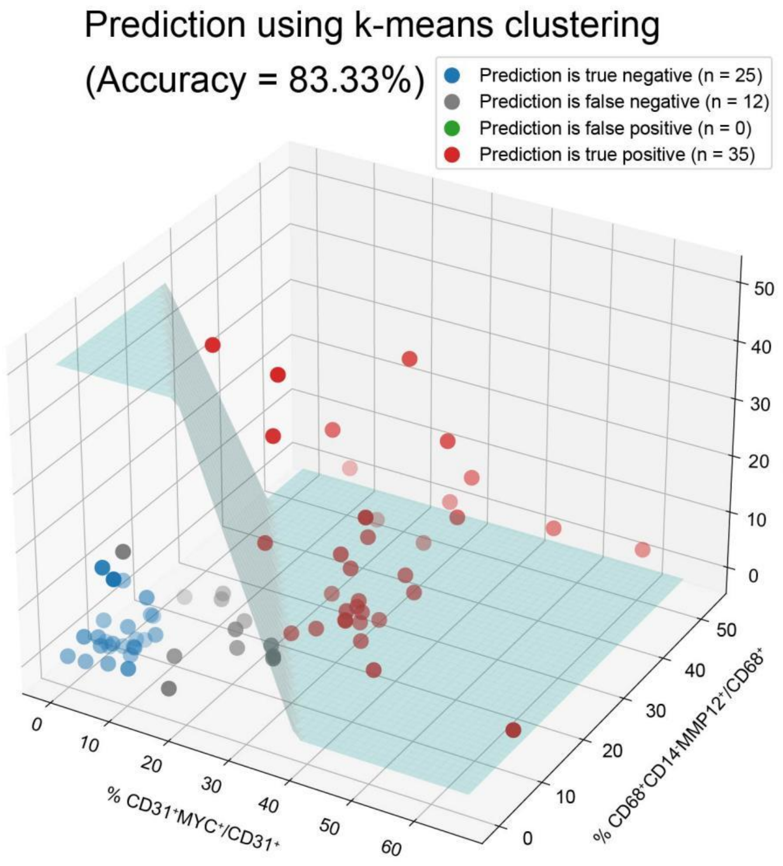
Prediction using k-means clustering
(Accuracy = 83.33%)
Prediction is true negative (n = 25)
Prediction is false negative (n = 12)
Prediction is false positive (n = 0)
Prediction is true positive (n = 35)
% CD31⁺MYC⁺/CD31⁺
% CD68⁺CD14⁻MMP12⁺/CD68⁺
% CD14⁺

Figure S23. Pro-inflammatory microenvironment were enriched in advanced human DILI. A) Microenvironmental cell frequency data of human DILI were plotted in a three-dimensional space (*n* = 10 with CHB, 5 with ALD, 5 with AIH, 5 with PBC and 47 with DILI). B) The k-means algorithm was applied to conduct an unsupervised clustering, and the prediction accuracy rate reached 83.33%. Detailed prediction model can be found in the Experimental Section.

Supplementary tables

Table S1. Detailed information of *in vivo* drug administrations.

Name	Function	Dose	Frequency	Time point[a)]	Mode	Solvent	Control group
AVE0991	Mas receptor agonist	1.16 [nmol g^{-1}]	One time	2 h before	Intraperitoneal	10% DMSO and 90% corn coil	Solvent
KJ Pyr9	MYC inhibitor	25 [mg kg^{-1}]	One time	2 h after	Intraperitoneal	DMSO: Tween 80: 5% dextrose = 1: 1: 8 by volume	Solvent
3PO	PFKFB3 inhibitor	50 [mg kg^{-1}]	Three times	① 16 h before ② 2 h after ③ 10 h after	Intraperitoneal	DMSO: PEG 300: Tween 80: Saline = 1: 4: 0.5: 4.5 by volume	Solvent
Clodronate liposomes	Macrophage depletion	8 [ml kg^{-1}]	One time	24 h before	Intravenous	/	Control liposomes
Cenicriviroc	Dual CCR2/CCR5 antagonist	100 [mg kg^{-1}]	One time	2 h before	Intragastric	DMSO: Tween 80: 5% dextro	Solvent

						se = 1: 1: 8 by volume	
Cediranib	VEGFR inhibitor	6 [mg kg^{-1}]	Three times	① 18 h before ② 2 h before ③ 5 h after	Intragastric	DMSO: Tween 80: 5% dextrose = 1: 1: 8 by volume	Solvent
(Rac)-Benpyrine	TNF-α inhibitor	30 [mg kg^{-1}]	Two times	① 2 h before ② 4 h after	Intragastric	DMSO: PEG 300: Tween 80: Saline = 1: 4: 0.5: 4.5 by volume	Solvent
R-7050	TNFR antagonist	10 [mg kg^{-1}]	One time	2 h before	Intraperitoneal	DMSO: PEG 300: Tween 80: Saline = 1: 4: 0.5: 4.5 by volume	Solvent

[a)] APAP administration was used as reference.

Abbreviations: VEGFR, vascular endothelial growth factor receptor; TNFR, tumor necrosis factor receptor; CCR, chemokine receptor.

Table S2. Demographic and clinical features of patients and HCs providing liver samples.

	DILI (*n* = 47)	HBV (*n* = 10)	ALD (*n* = 5)	NAFLD (*n* = 5)	AIH (*n* = 5)	PBC (*n* = 5)	HC (*n* = 14)
Age [years]	42.3 ± 15.4	49.4 ± 9.4	44.8 ± 10.8	37.8 ± 4.0	50.4 ± 9.2	54.8 ± 7.0	33.6 ± 5.0
Gender [F/M]	23/24	1/9	5/0	2/3	4/1	4/1	9/5
ALT [U L^{-1}]	364.9 ± 461.1	38.0 ± 40.1	39.0 ± 22.8	52.6 ± 29.5	55.2 ± 72.6	39.8 ± 25.5	25.3 ± 15.7
AST [U L^{-1}]	381.0 ± 636.7	53.4 ± 48.1	42.2 ± 20.2	35.8 ± 15.7	98.0 ± 139.2	68.8 ± 41.6	18.0 ± 5.7
ALP [U L^{-1}]	122.4 ± 95.4	135.3 ± 70.5	168.6 ± 71.2	76.6 ± 9.4	85.0 ± 37.0	237.6 ± 169.5	87.4 ± 28.1
γ-GT [U L^{-1}]	78.3 ± 110.5	61.7 ± 52.9	21.4 ± 4.5	31.8 ± 19.3	36.8 ± 21.0	81.6 ± 65.7	28.9 ± 45.4
TBIL [μmol L^{-1}]	376.5 ± 244.9	98.3 ± 186.0	280.9 ± 218.7	6.2 ± 1.7	221.0 ± 201.8	172.9 ± 146.5	9.4 ± 4.1
DBIL [μmol L^{-1}]	246.8 ± 162.3	56.4 ± 124.3	143.1 ± 155.7	2.9 ± 1.4	122.9 ± 119.6	168.5 ± 115.0	2.6 ± 1.3
PT [s]	33.3 ± 32.5	17.1 ± 4.5	25.0 ± 2.4	10.5 ± 0.6	23.6 ± 8.8	17.1 ± 2.6	10.9 ± 0.5

INR	2.8 ± 2.2	1.6 ± 0.5	2.4 ± 0.2	0.9 ± 0.1	2.2 ± 0.9	1.6 ± 0.2	0.9 ± 0.1

Abbreviations: F/M, Female and Male; ALT, alanine transaminase; AST, aspartate aminotransferase; ALP, alkaline phosphatase; γ-GT, γ-glutamyl transferase; TBIL, total bilirubin; DBIL, direct bilirubin; PT, prothrombin time; INR, international normalized ratio.
